# φ-Aromaticity in prismatic {Bi_6_}-based clusters

**DOI:** 10.1038/s41557-022-01099-5

**Published:** 2022-12-22

**Authors:** Benjamin Peerless, Andreas Schmidt, Yannick J. Franzke, Stefanie Dehnen

**Affiliations:** 1 Fachbereich Chemie, Philipps-Universität Marburg, Marburg, Germany; 2 Institute of Nanotechnology, Karlsruhe Institute of Technology (KIT), Karlsruhe, Germany

**Keywords:** Organometallic chemistry, Chemical bonding, Quantum chemistry, Density functional theory

## Abstract

The occurrence of aromaticity in organic molecules is widely accepted, but its occurrence in purely metallic systems is less widespread. Molecules comprising only metal atoms (M) are known to be able to exhibit aromatic behaviour, sustaining ring currents inside an external magnetic field along M–M connection axes (σ-aromaticity) or above and below the plane (π-aromaticity) for cyclic or cage-type compounds. However, all-metal compounds provide an extension of the electrons’ mobility also in other directions. Here, we show that regular {Bi_6_} prisms exhibit a non-localizable molecular orbital of *f*-type symmetry and generate a strong ring current that leads to a behaviour referred to as φ-aromaticity. The experimentally observed heterometallic cluster [{CpRu}_3_Bi_6_]^–^, based on a regular prismatic {Bi_6_} unit, displays aromatic behaviour; according to quantum chemical calculations, the corresponding hypothetical Bi_6_^2−^ prism shows a similar behaviour. By contrast, [{(cod)Ir}_3_Bi_6_] features a distorted Bi_6_ moiety that inhibits φ-aromaticity.

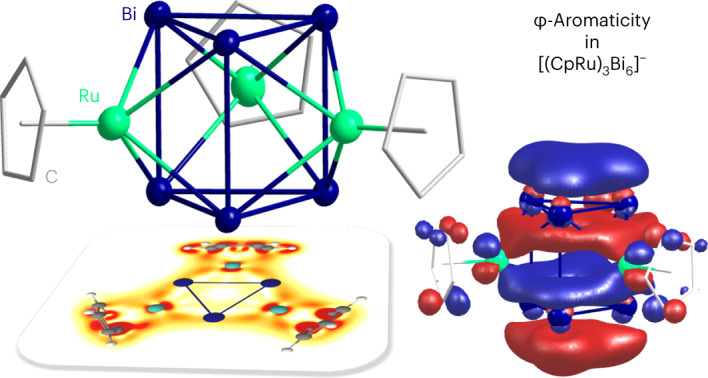

## Abstract (web summary)

Aromaticity is a ubiquitous concept in organic chemistry yet it is less widespread for inorganic species. Now the cluster [(CpRu)_3_Bi_6_]^–^, obtained as part of a soluble salt, has been shown to exhibit aromatic behaviour referred to as φ-type, owing to a highly regular {Bi_6_} substructure causing a non-localizable molecular orbital of $f_{z^3}$-like symmetry.

## Main

Aromaticity is a ubiquitous concept taught to all chemistry students, and yet can arguably be regarded as one of the most difficult to define. Originally, organic aromatic molecules were defined as such if they were cyclic, planar and unsaturated with substantial delocalization of 4*n* + 2 *π*-electrons over the ring^1^. However, aromaticity is not a directly observable quantity, leading to efforts to quantify such a phenomenon being a contentious issue^2–7^. Coupling with the fact that aromaticity has substantial effects on the stability, geometrical structure, reactivity patterns and magnetic properties of such molecules adds to the complexity of the problem. Quantitative measures have been used to encompass these effects to assign aromaticity, such as chemical shifts in nuclear magnetic resonance (NMR) experiments and bond lengths obtained from X-ray diffraction data. One conventional measure used in this vein is the nucleus-independent chemical shift (NICS), a theoretical calculation that takes advantage of the ability of these systems to sustain a ring current in an external magnetic field^8^. However, NICS does not assess the ring current directly, but rather assesses the impact of the ring current on a ghost atom; that is, the NMR shielding constant is calculated for a massless and chargeless atom in the centre of the molecule. For monocyclic molecules, the placement of the ghost atom is straightforward. It is a different matter, however, for more complex current delocalization pathways or multicyclic molecules^9^. Furthermore, NICS also depends on the distance of the ghost atom from the bonds sustaining the ring current, as the shielding effect is driven by the accumulation of electron density and/or its response due to the magnetic field^10^. Owing to the small distance of the ghost atom from the bonds and the associated electron density, this leads to comparably large NICS values for triangular systems. A more flexible approach is to calculate the ring current directly^5^ based on numerical integration of the magnetically induced current density^11,12^. One such approach is the gauge-including magnetically induced current (GIMIC) method^13,14^.

The rules drawn up to assign aromaticity were originally applied to hydrocarbon-based systems (C_*n*_R_*n*_; R = H, organic substituent) and by extension to cyclic molecules containing a heteroatom (C_*n*_R_*n*_X; X = N, P, O, S or other *p*-block element), defining the class of aromatic organic molecules. Additionally, several examples of metallocycles containing transition metals (C_*n*_R_*n*_M; M = transition metal) have been classed as aromatic, and the number of examples continues to grow at an exceptional rate since the first such example, [Os(C_5_H_4_S)(PPh_3_)_2_(CO)] (refs. ^15–17^), an ‘osmabenzene’.

Yet the synthesis of aromatic molecules consisting exclusively of metal atoms is still very much in its infancy, despite the first example, [(RGa)_3_]^2−^ (R = 2,6-dimesitylphenyl), being reported more than two decades ago^18^. This is mostly because metals tend to form either extended structures or discrete polyhedral structures as opposed to the cyclic molecules common to organic chemistry. However, due to the preference for metallic bonding, strong electron delocalization is still apparent in polymetallic molecules, paving the way for interesting and unique bonding models derived to explain experimental observations. The models and associated terminology of ‘all-metal aromaticity’ have caused vivid discussions among the scientific communities^19–21^. The representative course of the ring current along the atom–atom connection axes within cycles or polyhedra invoked the term σ-aromaticity^22–26^, which was used to describe the electronic situation of a number of polymetallic molecules to partner the universally accepted π-aromaticity typical of organic molecules. By extension, theoretical work on ring currents in coinage metal and uranium clusters involving the participation of *d* and *f* orbitals^27,28^, respectively, and hypothetical considerations of *f*-block-based trimetallic cyles^29^ put forward evidence that aromaticity was not reserved for molecular orbitals (MOs) of σ- and π-symmetry, but also for ones of δ- or φ-symmetry, without experimental proof of the latter. The idea of σ-aromaticity especially invited controversial discussion, as it can be viewed as just an alternative to the widely accepted notion of cluster orbitals and the concepts in superatom theory or the Jellium model^30^. Nevertheless, throughout this debate, the salient point to result from the discussion is the fact that polymetallic molecules can exhibit aromatic properties—a remarkable discovery that opens up pathways into new and exciting theoretical and synthetic chemistry that can challenge and enlighten our ideas of bonding and aromaticity.

As group 15 elements are valence isoelectronic to {CH}, the most direct transfer of the aromaticity concept from organics to molecules containing only metal atoms is for poly(antimony) or poly(bismuth) species. Indeed, regarding poly(pnictogen) molecules in general, this has been realized for planar P_5_^−^ and P_6_ bonded to transition metal complexes^31,32^, as well as for pertinent examples of arsenic and antimony systems^33,34^. However, the tendency remains that the heavier elements of group 15 prefer to expand towards higher-dimensionality structures—two-dimensional, as in the element or nanostructured excerpts such as bismuthene^35^—or towards non-planar molecular structures^36^. Yet the latter can still exhibit aromatic properties, as seen in the example of [Th@Bi_12_]^4−^ (ref. ^10^), which shows a substantial π-type ring current in a torus-shaped metal cluster^10,37^. Hence, unlike benzene, where the planar six-membered ring is aromatic and the three-dimensional regular triangular prism structure, prismane^38,39^, is not, a {Bi_6_} prism has potential as an aromatic polyhedral molecule that exceeds the ‘normal’ σ-delocalization towards π-symmetry or other symmetries. Our interest in poly(bismuth) structures has driven us to investigate the synthesis of a {Bi_6_} prism trapped by transition metal fragments that can be used to fine-tune the structural and electronic situation on the {Bi_6_} unit, and this way control its aromatic properties.

An additional motivation for this study stems from the interest in using homoatomic molecular species of heavy *p*-block (semi)metals for the formation of corresponding nanostructures. Elemental bismuth comprises two-dimensional sheets of fused corrugated six-membered rings like grey arsenic and antimony, and is similar to the structure of black phosphorous, whereas highly reactive yet isolable molecular forms P_4_ and As_4_ are known only for the lighter congeners, phosphorus and arsenic^40^. To date, the only molecular bismuth units isolated are polybismuth cations^41–43^ and the Zintl anions Bi_2_^2−^, Bi_4_^2−^, Bi_7_^3−^ and Bi_11_^3−^ (refs. ^44–47^), generated by extraction of various intermetallic solids, such as K_5_Bi_4_ (refs. ^44,45^) and K_5_Ga_2_Bi_4_ (ref. ^48^), and subsequent (partial) oxidation in polar solvents like ethane-1,2-diamine (en), *N*,*N*-dimethylformamide (DMF) and pyridine. The process of oxidation results in the coupling of smaller {Bi_*x*_} units into larger cluster anions, though the mechanisms still remain poorly understood despite recent advancements. Heterometallic clusters containing a majority of bismuth atoms can be prepared in a similar fashion to the homonuclear clusters by addition of a transition metal or lanthanide complex during extraction. However, intermetallic solids containing Bi atoms and (polyanionic) {Bi_*x*_} substructures often have poorly defined structures causing issues with predictability and control, and hence the search for molecular {Bi_*x*_} units continues to be a challenge. Diligent work by research groups worldwide has shown that oxidative coupling reactions yield a plethora of interesting cluster molecules with varied structural motifs, like ozone-like {Bi_3_} (ref. ^49^), {Bi_4_) rings^50^ and a {Bi_6_} chair and (distorted) triangular prism^51,52^. These results led us to design a synthetic strategy to build polyhedral cages by aggregation with Lewis-acidic transition metal complexes with relatively low redox properties and soluble Bi_2_^2−^, so as to trigger a controlled oxidative coupling. Hence, we are moving away from heterogeneous approaches involving ill-defined nominal intermetallic solids and developing a methodology using a homogeneous ‘bottom-up’ approach.

**Fig. 1:**
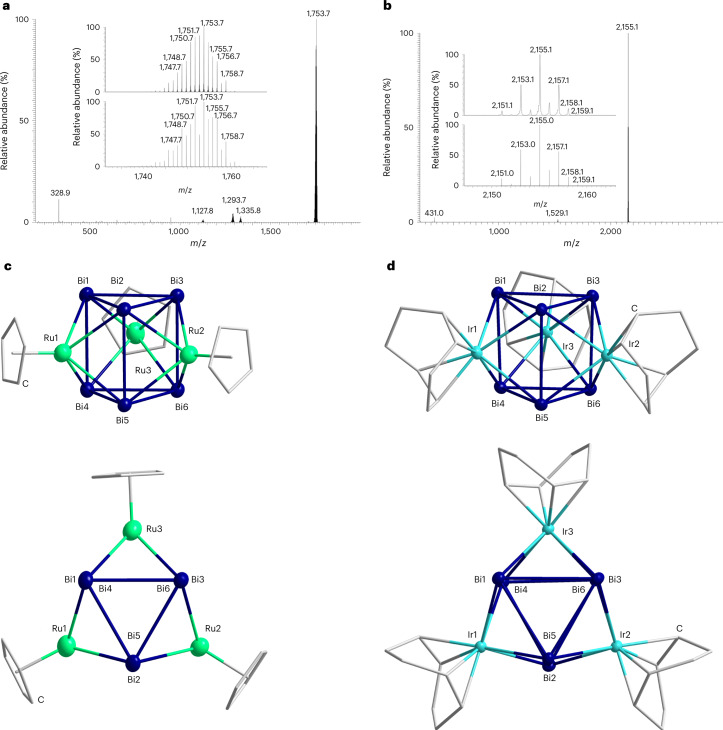
Mass spectra and molecular structures of the cluster anions 1^–^ and 2^–^. **a**, Electrospray-ionization mass spectrum of the anion **1**^**–**^ at *m*/*z* = 1,753.7 (high-resolution spectrum in the inset with measured (top) and simulated (bottom)) obtained in negative-ion mode. **b**, Negative-ion mode mass spectrum of the anion **2**^**–**^ at *m*/*z* = 2,155.1 (high-resolution spectrum in the inset with measured (top) and simulated (bottom)). **c**, Molecular structure of **1**^**–**^ in a side view (top) and a top view (bottom) of the prismatic substructure, shown for one of the two very similar individual cluster molecules in two different orientations, with displacement ellipsoids drawn at the 50% probability level and H atoms omitted for clarity. Ru, green; Bi, blue; C, grey. Ranges of selected distances, given for one of two very similar individual cluster anions in the crystal (in angstroms): Bi–Bi within triangle, d_A_, 3.0995(9)–3.1355(10); Bi–Bi between triangles, d_B_, 3.2755(10)–3.3295(10); Ru–Bi, 2.7620(18)–2.8121(16). **d**, Molecular structure of **2**^**–**^ in a side view (top) and a top view (bottom) of the prismatic substructure, shown in two different orientations, with displacement ellipsoids drawn at the 50% probability level and H atoms omitted for clarity. Ranges of selected distances, given for one of two very similar individual cluster anions in the crystal (in angstroms): Bi–Bi within triangles, d_A_, 3.0709(5)–3.2593(5); Bi–Bi between triangles, d_B_, 3.2522(4)–3.2742(4); Ir–Bi, 2.7612(5)–2.8539(5). More details regarding the X-ray structures are given in Supplementary Table 1 and Supplementary Figs. 1 and 2.

Here, we present the synthesis and crystal structures of [K(crypt-222)][{CpRu}_3_Bi_6_]·0.5tol ([K(crypt-222)]**1**·0.5tol) and [K(crypt-222)][{(cod)Ir}_3_Bi_6_]·tol ([K(crypt-222)]**2**·tol) (crypt-222, 4,7,13,16,21,24-hexaoxa-1,10-diazabicyclo[8.8.8]hexacosane; cod, 1,5-cyclooctadiene; tol, toluene; and Cp, cyclopentadienide), which feature a prismane-like {Bi_6_} unit. Additionally, we report in-depth quantum chemical studies on both the entire cluster molecules, as well as the hypothetically underlying ‘Bi_6_^*q*–^’ anion (*q* = 0–4). Our findings show that the structural properties are strictly correlated with the number of electrons on the {Bi_6_} unit (represented by the charge *q*), which in turn defines its aromatic properties. Clusters with up to *q* = 2 form regular prisms (such as that found in [{CpRu}_3_Bi_6_]^–^, referred to as **1**^**–**^; Fig. 1a,c), whereas a distortion of the unit (such as found in [{(cod)Ir}_3_Bi_6_]^–^, referred to as **2**^**–**^; Fig. 1b,d) occurs for *q* = 3 and 4. At the same time, ‘Bi_6_^2–^’ is a hypothetical aromatic molecule sustaining an exceptionally strong diatropic ring current of +31.1 nA T^–1^ with a non-localizable MO (compare with +11.8 nA T^–1^ for six *π*-electrons in benzene calculated with the same computational methods herein), while ‘Bi_6_^*q*–^’ clusters with charges of *q* ≠ 2 (or no charge at all) show decreased values of the ring current^10,37^.

**Fig. 2:**
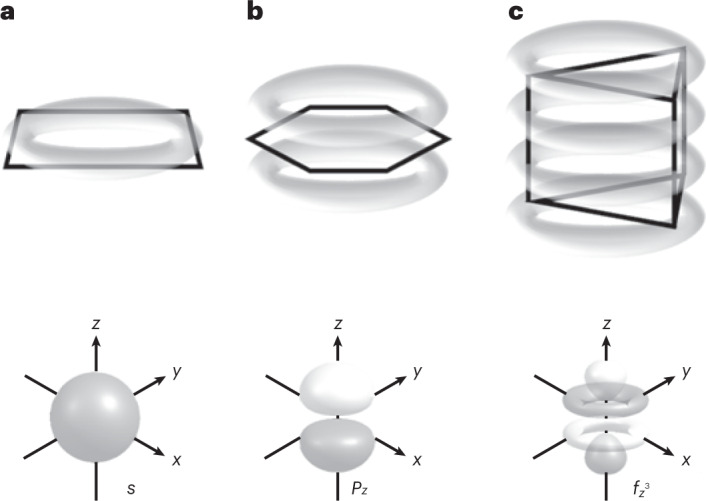
Schematic representation of σ-, π- and φ-type aromaticities by illustration of the shape of their maximum ring current densities. **a**, The σ-type aromaticity in a planar cycle, like a Cu_4_^2^^–^ dianion^57^, and the angular-dependent part of an *s*-type atomic orbital for comparison. **b**, The π-type aromaticity in a planar cycle like benzene and the angular-dependent part of a *p*_*z*_-type atomic orbital for comparison. **c**, The φ-type aromaticity in prismatic Bi_6_^2–^ presented in this work and the angular-dependent part of an ${{{{f}}}}_{{{{{z}}}}^3}$-type atomic orbital for comparison.

The behaviour of Bi_6_^2–^ is transferred to the heteroatomic cluster that is based on the respective, highly symmetric {Bi_6_} unit, with a strong diatropic ring current of +25.6 nA T^–1^ for [{CpRu}_3_Bi_6_]^–^. We note that these are very large values for the ring current, and that the symmetry of the respective MO is similar to that of the ${f}_{{z}^3}$ atomic orbital; therefore we propose the term ‘φ-aromaticity’. Figure 2 illustrates the typical shape of the maximum ring current densities for σ-type and π-type aromaticity, and also for the higher-dimensionality φ-type aromaticity.

## Results and discussion

### Synthesis and structure of [{LM}_3_Bi_6_]^–^ clusters

Typically, the synthesis of large polybismuth anions, with or without metal complexes bonded to the cluster frame, begins with ill-defined intermetallic solids, such as K_5_Bi_4_ and K_5_Ga_2_Bi_4_, among others. The previously reported procedure for the isolation of pure [K(crypt-222)]_2_Bi_2_ (**A**)^44^ was scaled up to 0.50 mmol to allow us to obtain suitable quantities of a defined molecular precursor, which we have successfully used for the formation of salts of the clusters [{CpRu}_3_Bi_6_]^–^ (**1**^**–**^) and [{(cod)Ir}_3_Bi_6_]^–^ (**2**^**–**^), as detailed in the following.

The reaction of **A** with one equivalent of [CpRu(MeCN)_3_][PF_6_] performed in en affords a brown solution immediately. Time-dependent electrospray-ionization mass spectrometry measurements of the reaction solution showed a single ion peak in the negative-ion detection mode, which belongs to [{CpRu}_3_Bi_6_]^−^ (Fig. 1a). Filtration, subsequent layering with toluene and *n*-hexane, and storage at 5 °C over several days afforded dark brown crystals suitable for single-crystal X-ray diffraction. The crystals served to identify the compound as [K(crypt-222)]**1**·0.5tol. The composition of the crystals was verified by micro-X-ray fluorescence and in solution via NMR spectroscopy, accordingly (Supplementary Table 2 and Supplementary Figs. 3, 6 and 7). In addition, substituting [CpRu(MeCN)_3_][PF_6_] with the [(cod)Ir]^+^ source [(cod)IrCl]_2_ in the reaction also yields a brown solution. Time-dependent electrospray-ionization mass spectrometry measurements of the in situ reaction solution (Supplementary Fig. 5) clearly show the presence of an anion related to the one in [K(crypt-222)]**1**·0.5tol with the composition [{(cod)Ir}_3_Bi_6_]^−^, along with another signal that has a mass-to-charge ratio *m*/*z* of 1,529.1 with appropriate fragmentation consistent with the loss of subsequent cod ligands as the sole species in the spectrum. The second species can be assigned to a molecule representative of [{(cod)Ir}_3_Bi_3_H]^−^. Following the same procedure as that described previously for [K(crypt-222)]**1**·0.5tol, a few milligrams of small, dark brown block crystals of [K(crypt-222)]**2**·tol was achievable over several days. The composition of the crystals was determined by single-crystal X-ray diffraction and further verified by micro-X-ray fluorescence measurements (Supplementary Table 2 and Supplementary Fig. 4). The second observed species in the electrospray-ionization mass spectrometry was not isolable from the reaction solution, nor does it appear in the mass spectrum of redissolved single crystals of [K(crypt-222)]**2**·tol (Fig. 1b), indicating that it is not a fragment of the latter. The ^1^H and ^13^C{^1^H} NMR spectra of [K(crypt-222)]**2**·tol in DMF-d_7_ show the typical resonances for crypt-222 and the cod ligands in an approximate ratio of 1:3, consistent with the structure obtained in the solid state (Supplementary Figs. 8 and 9). Larger quantities of a crystalline material of both products, [K(crypt-222)]**1**·0.5tol (35% yield) and [K(crypt-222)]**2**·tol (58% yield), were obtainable by evaporation of the reaction solution after filtration and washing the residue with acetonitrile.

The single-crystal X-ray structure analyses of [K(crypt-222)]**1**·0.5tol and [K(crypt-222)]**2**·tol show that both cluster anions adopt the same overall architecture as a nine-vertex closo-deltahedron. Figure 1c,d illustrates the molecular structure of the two monoanionic clusters. Two {Bi_3_} triangles come together to form a trigonal prism with each of the rectangular faces capped with the relevant transition metal fragment. Two different Bi–Bi bonds are present, with the Bi–Bi bonds inside the {Bi_3_} triangle of the prism (d_A_) on average slightly shorter in length than the Bi–Bi bond that connects the triangles together (d_B_). While both anions possess the same overall cluster structure, the {Bi_6_} unit differs notably between the two cluster anions, **1**^**–**^ and **2**^**–**^. Whereas in **1**^**–**^, each of the d_A_ bonds are very similar in length 3.0995(9)–3.1355(10) Å, 3.120(1) Å on average), the d_A_ bond length range in **2**^**–**^ is much larger (3.0709(5)–3.2593(5) Å, 3.146(1) Å on average) and displays the pattern of one short, one middling and one long bond relative to one another in each triangle. The average d_B_ bond length in **1**^**–**^ and **2**^**–**^ is 3.307(1) Å and 3.266(1) Å, respectively. Remarkably, the range of observed d_B_ bond lengths in **1**^**–**^ is less than 0.1 Å 3.2755(10)–3.3295(10) Å), in stark contrast to the structural properties of [{(CO)_3_Mo}_3_Bi_6_]^4−^ (ref. ^52^), the only other known structure containing a trigonal prismatic {Bi_6_} subunit, which displays a much more significant distortion of the prism (d_A_ of 3.0664(9)–3.199(1) Å, d_B_ of 3.1740(7)–3.5554(7) Å)^52^—which is crucial for the electronic structures and bonding properties, as discussed in more detail in the following.

Both of the anions **1**^**–**^ and **2**^**–**^ possess an overall −1 charge, the lowest observed for a binary cluster of bismuth and a transition metal, and generally rare owing to the crystallization with only one [K(crypt-222)]^+^ cation. The question that occurs at this point is which charge may be attributed to the {Bi_6_} prism. A simple assignment of oxidation states on the basis of first principles results in a Bi_6_^4−^ capped with three positively charged [CpRu(II)]^+^ and [(cod)Ir(I)]^+^ fragments in **1**^**–**^ and **2**^**–**^, respectively. The same assignment was given to [{(CO)_3_Mo}_3_Bi_6_]^4−^ (ref. ^52^). However, this simple assignment neglects a potential inclusion of the transition metal into the cluster bonding as a whole and the clearly observable structural effect on the {Bi_6_} prism. Detailed quantum chemical calculations documented in the following sections demonstrate the substantial role of the nature of the complex fragments used for the stabilization of the {Bi_6_}, and they serve to answer two further questions, namely, (1) how this is controlled and thus varies with the nature of the transition metal complex, and (2) how the electronic situation is affected by such changes.

### Quantum chemical study of Bi_6_^*q*–^ and [{LM}_3_Bi_6_]^−^

**Fig. 3:**
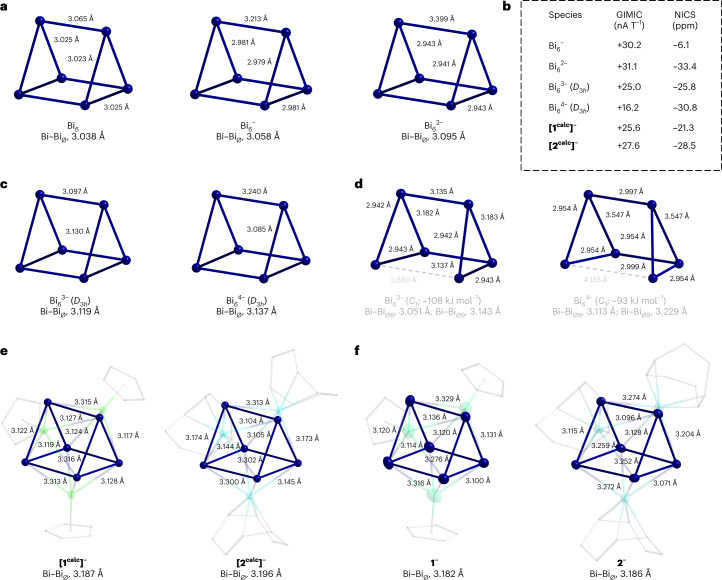
Comparison of structural and magnetic response data of calculated clusters Bi_6_^*q*–^ (*q* = 0–4) and the {Bi_6_} prisms in the calculated and the experimentally observed molecular structures of the anions 1^–^ and 2^–^. **a**, From left, the calculated prismatic structures of Bi_6_, Bi_6_^–^ and Bi_6_^2–^, indicating an overall increase of (mean) Bi–Bi distance with increasing negative charge. **b**, Summary of magnetic response data of calculated prismatic clusters Bi_6_^*q*–^ (*q* = 1–4) and the {Bi_6_} prisms present in the calculated molecular structures **[1**^**calc**^**]**^**–**^ and **[2**^**calc**^**]**^**–**^, given within a dashed box for clarity (the ghost atom for NICS is placed at the centre of mass of the cluster); positive values for GIMIC and negative values for NICS indicate aromatic properties, in all cases, yet with slightly differing net values. **c**, The more symmetric, but energetically less favourable, isomers of Bi_6_^3–^ (*D*_3*h*_ symmetry) and Bi_6_^4–^ (*D*_3*h*_ symmetry). **d**, The non-prismatic energetic minimum structures of Bi_6_^3–^ (favoured by 107.7 kJ mol^–1^) and Bi_6_^4–^ (favoured by 92.6 kJ mol^–1^). **e**, The {Bi_6_} prisms in the calculated structures **[1**^**calc**^**]**^**–**^ and **[2**^**calc**^**]**^**–**^. **f**, The {Bi_6_} prisms in the experimentally observed structures of the cluster anions **1**^**–**^ and **2**^**–**^. Atom colour code is Bi, blue; Ru, green; Ir, cyan; and C, grey (wires); H atoms omitted for clarity.

To get an idea of the charge and electronic situation of the {Bi_6_} units included in the clusters **1**^**–**^ and **2**^**–**^, we started out with structure optimizations of the hypothetical ‘naked’ prismatic Bi_6_^*q*–^ clusters with charges 0, –1, –2, –3, and –4. The resulting structures are shown in Fig. 3, in comparison with the respective structures of the experimentally observed prisms in **1**^**–**^ and **2**^**–**^, and their computed counterparts **[1**^**calc**^**]**^**–**^ and **[2**^**calc**^**]**^**–**^, along with the magnetic response data given as ring currents (current strengths calculated with GIMIC) and NICS values (Fig. 3b).

A comparison of the structural data allows two important conclusions. First, prisms with charges of 0, –1 or –2 are comparably regular (Fig. 3a), while more electrons lead to a notable distortion of the global minimum structures; hence, a free Bi_6_^4–^ (isoelectronic with known Te_6_^2+^)^53^ would never be prismatic. The interpretation of the {Bi_6_} units in the heterometallic clusters as a fourfold-charged prism would therefore require that the energy gained by coordination of the transition metal complex fragments overcompensates the energy required to stabilize this highly unfavourable architecture (Fig. 3c,d). Second, the problems of the (computationally enforced) *D*_3*h*_ structure of Bi_6_^4–^ are clearly visible in the development of the Bi–Bi distances: within the triangles (comprising the stronger bonds), the Bi–Bi distances decrease (that is, get stronger) from the uncharged prism (3.02 Å) through the monoanion (2.98 Å) to the dianion (2.94 Å), while they significantly increase upon addition of another two electrons in the Bi_6_^4–^ prism (3.09 Å). We therefore assume that these Bi–Bi distances within the experimentally observed structures (which are naturally longer on average owing to the coordination of the transition metal complexes; see the calculated analogues) are a sensitive indicator for the actual degree of charge overload on the prism. The distances between the triangles (comprising the weaker Bi–Bi interactions) initially increase by approximately 0.2 Å per charge (3.07 Å in the neutral prism, 3.21 Å in the monoanion, 3.40 Å in the dianions), but fall back to the value of the monoanion for the prismatic Bi_6_^4–^, while the triangles widen at the same time. Hence, the average Bi–Bi bond lengths develop from 3.04 Å in the hypothetical ‘elemental’ Bi_6_ to 3.14 Å in the tetraanion, which is therefore characterized by relatively weak Bi–Bi bonds throughout.

This trend can be rationalized by the Wiberg bond indices (WBIs)^54^. The ‘elemental’ Bi_6_ consists of single Bi–Bi bonds only (WBI of 0.99 for all Bi–Bi bonds). A notable multibond character within the Bi triangles is revealed for the dianion. We obtain a WBI of 1.14 for the Bi triangles and a WBI of 0.89 between the triangles—in agreement with the shorter bond lengths within the triangles and increased bond lengths between the two triangles. In the (unfavourable) prismatic isomers of Bi_6_^3–^ and Bi_6_^4–^, either the bonds within the triangles are substantially weakened or the Bi–Bi interaction between them is. Bi_6_^4–^ exhibits an average WBI of only 0.90 in this isomeric form.

**Fig. 4:**
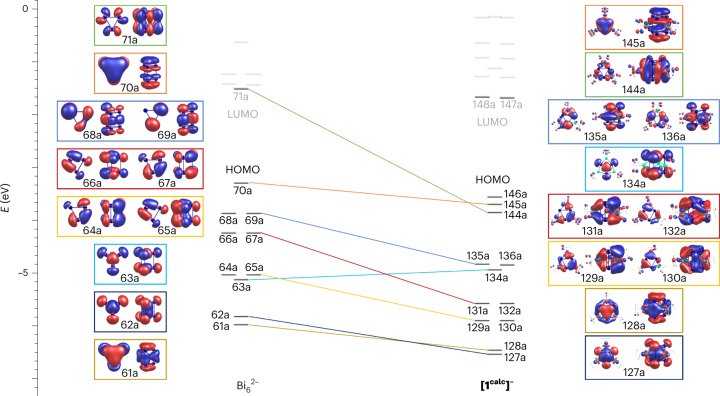
Frontier MO scheme of Bi_6_^2–^ (left) and of **[1**^**calc**^**]**^**–**^ (right). Selected MOs (with Bi-orbital contributions of 20% or higher) are plotted and connected, using a specific colour for each of the pairs, to illustrate the close relationship between the two clusters. All contours are drawn with an isovalue of ±0.027 a.u. Lines between the MO levels of Bi_6_^2–^ and **[1**^**calc**^**]**^**–**^, in colours consistent with those outlining the different MO representations, serve as a guide to the eye. LUMO, lowest unoccupied MO.

Regarding the heteroatomic clusters (Fig. 3e,f), the anion in compound **2**^–^ possesses an average Bi–Bi bond length of 3.146 Å in the triangular faces, while they are a bit shorter in anion **1**^**–**^ (3.120 Å on average), in agreement with the computed structures (3.187 Å on average for **[1**^**calc**^**]**^**–**^, 3.196 Å on average for **[2**^**calc**^**]**^**–**^). While this is not a big difference, it may be taken as evidence for a somewhat smaller charge overload in the {Bi_6_} unit of the Ru compound—consistent with the theoretical findings (more information in the following). A natural bond orbital analysis^55^ with the all-electron scalar-relativistic exact two-component (X2C) Hamiltonian confirms that the {Bi_6_} unit of the Ir-based cluster accumulates more electron density at the *p* orbitals than the Ru-based cluster (number of excess electrons is 0.65 for **[2**^**calc**^**]**^**–**^ versus 0.22 for **[1**^**calc**^**]**^**–**^). By comparison, the even more distorted {Bi_6_} core of [{(CO)_3_Mo}_3_Bi_6_]^4−^, reported in the literature^52^, correspondingly features a larger number of excess *p* electrons at 0.90, and hence accumulates more negative charge in line with the increased degree of distortion from (underlying) Bi_6_^2–^ to Bi_6_^4–^. Moreover, the Ru atoms possess a significantly larger negative charge than the Ir atoms, which we ascribe mainly to the stronger electron-withdrawing nature of the Cp ligands (at the Ru atoms) than that of the cod ligands (at the Ir atoms). The impact of spin–orbit coupling on the bonding situation is negligible, and the respective self-consistent two-component calculations confirmed the previous findings. Most notable, though, is the much more regular shape of the {Bi_6_} prism in **1**^**–**^, as compared to a substantially distorted polyhedron in **2**^**–**^, which is excellently reproduced by the density functional theory calculations, with a narrow range of Bi–Bi distances within the triangles in **[1**^**calc**^**]**^**–**^ (d_A_ of 3.117–3.128 Å; compare with the experimental d_A_ of 3.0995(9)–3.1355(10) Å) and a broader range of these distances in **[2**^**calc**^**]**^**–**^ (d_A_ of 3.104–3.174 Å; compare with the experimental d_A_ of 3.0709(5)–3.2593(5) Å). These findings are in full agreement with markedly different electronic structures. In Fig. 4, we compare the MO schemes of hypothetical Bi_6_^2–^ and **[1**^**calc**^**]**^**–**^, which underscores their close electronic and structural relationship. Lower MO energy levels in the case of the heterometallic cluster are due to the polarizing effects of the Lewis-acidic {RuCp} units attached to the {Bi_6_} moiety. Consequently, this effect is smaller for MOs in which the contribution of the {RuCp} units is comparably small (mainly 145a, 134a; also 127a (Fig. 4)). These MOs thus show the closest relationship with those of Bi_6_^2–^, and they include the highest occupied MO (HOMO) of the latter and the HOMO – 1 of **[1**^**calc**^**]**^**–**^, which is crucial for the observed aromatic behaviour of both (more information in the following). All of these findings suggest that the regular {Bi_6_} prism in the Ru-based cluster **1**^**–**^ can be interpreted as a twofold charged unit, whereas the substantially distorted {Bi_6_} units found in the Ir-based cluster **2**^**–**^ and the related [Bi_6_Mo_3_(CO)_9_]^4–^ cluster^52^ are closer to the description of a Bi_6_^4–^ core.

### Notable φ-aromaticity of [{CpRu}_3_Bi_6_]^–^ and model system Bi_6_^2–^

Both heterometallic clusters **1**^**–**^ and **2**^**–**^, as well as the related cluster [{(CO)_3_Mo}_3_Bi_6_]^4−^ (ref. ^52^), sustain a strong net diatropic ring current of more than +20 nA T^–1^, calculated with GIMIC (Fig. 3, Supplementary Tables 3–7 and Supplementary Section 6). Results with the NICS approach are in line with the direct computation of the ring current. We use the computationally optimized structure for **1**^**–**^ and **2**^**–**^, while we employ both the experimentally determined and computationally optimized structures for [{(CO)_3_Mo}_3_Bi_6_]^4−^, as the two methods lead to larger geometric deviations for this cluster without notably affecting the magnetic response properties.

**Fig. 5:**
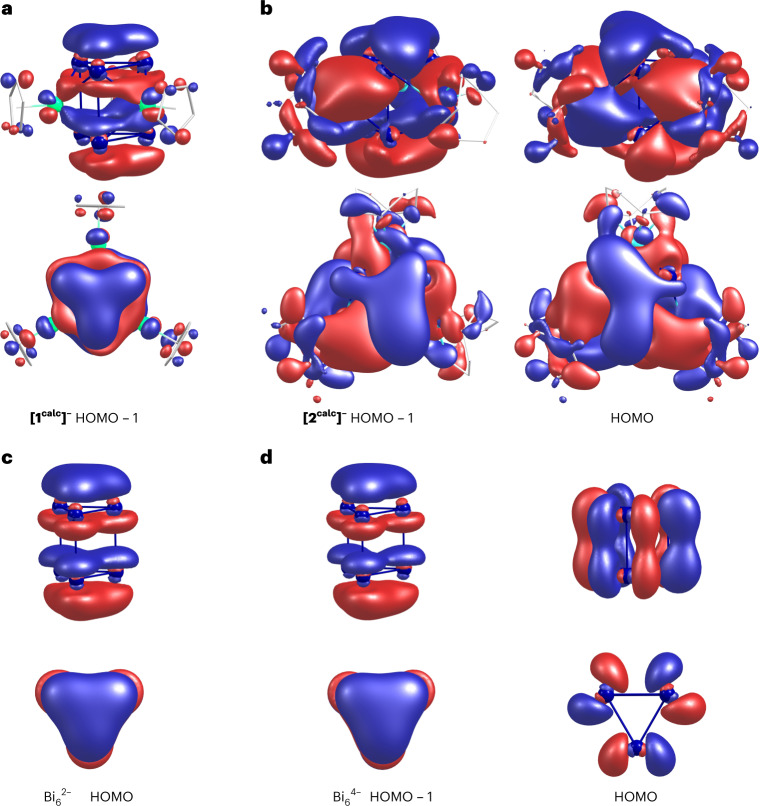
Delocalized (canonical) MOs of [1^calc^]^–^, [2^calc^]^–^ and prismatic Bi_6_^*q*–^ (*q* = 2, 4). **a**, HOMO – 1 of **[1**^**calc**^**]**^**–**^ possessing φ-symmetry. **b**, HOMO – 1 and HOMO of **[2**^**calc**^**]**^**–**^ with (highly distorted) φ-type symmetry. **c**, HOMO of Bi_6_^2–^ possessing φ-symmetry. **d**, HOMO – 1 possessing φ-symmetry and HOMO of prismatic Bi_6_^4–^. Note that the contours for **[1**^**calc**^**]**^**–**^ and Bi_6_^*q*–^ (*q* = 2, 4) were drawn with an isovalue of ±0.027 a.u., while the isovalue of **[2**^**calc**^**]**^**–**^ required a decrease to 0.015 a.u. in order to make the (rudimentary) φ-type MOs visible at all. Atom colour code is Bi, blue; Ru, green; Ir, turquoise; and C, grey (wires); H atoms omitted for clarity.

Of the three heterometallic clusters, **[1**^**calc**^**]**^**–**^ and [{(CO)_3_Mo}_3_Bi_6_]^4−^ clearly feature orbitals with φ-type symmetry, reminiscent of an $f_{z^3}$ atomic orbital. However, only in the case of **1**^**–**^ is the corresponding MO of a very regular shape (Fig. 5a and the following text), while for [{(CO)_3_Mo}_3_Bi_6_]^4−^, this MO exhibits substantial distortions (as shown in the literature^52^ and Supplementary Fig. 31). Upon very close inspection of the canonical orbitals of **[2**^**calc**^**]**^**–**^ (and by a substantial decrease of the contour threshold), a φ-type MO can also be identified for this species (Fig. 5b), but the deviation from a regular φ-symmetry is even more obvious here. Correspondingly, the current flows of all three heterometallic clusters show a notable degree of aromaticity according to the magnetic criterion, but the Ru-based cluster **[1**^**calc**^**]**^**–**^ is the only one in which this comes along with a regular φ-type MO and a highly symmetric architecture, thus also meeting the structural criterion.

To better understand and illustrate the nature of the aromaticity in the regular cluster anion **[1**^**calc**^**]**^**–**^, we also inspected the corresponding properties of the hypothetical model system Bi_6_^2–^. It also sustains a strong net diatropic ring current of about +30 nA T^–1^. This further establishes a close relationship of the model compound with the experimental cluster, which is also reflected in the relevant canonical MOs. Both the HOMO of Bi_6_^2–^ shown in Fig. 5c and the HOMO – 1 of **[1**^**calc**^**]**^**–**^ (Fig. 5b) consist of coexisting π-type contributions from atomic *p* orbitals in both triangles. Together, these yield one single, doubly occupied φ-type cluster orbital. This MO cannot be fully localized in these systems (Supplementary Fig. 13 for Bi_6_^2–^), and consequently, a remarkably strong diatropic ring current is sustained in the presence of an external magnetic field. This φ-type orbital covers the complete Bi_6_^2–^ prisms. Matters are qualitatively similar for Bi_6_^4–^, when restricted to *D*_3*h*_ symmetry (Fig. 5d), but the occupation of the HOMO (that is, the lowest unoccupied MO of Bi_6_^2–^) and the respective nodal structure cause a drop in ring currents (Fig. 3).

**Fig. 6:**
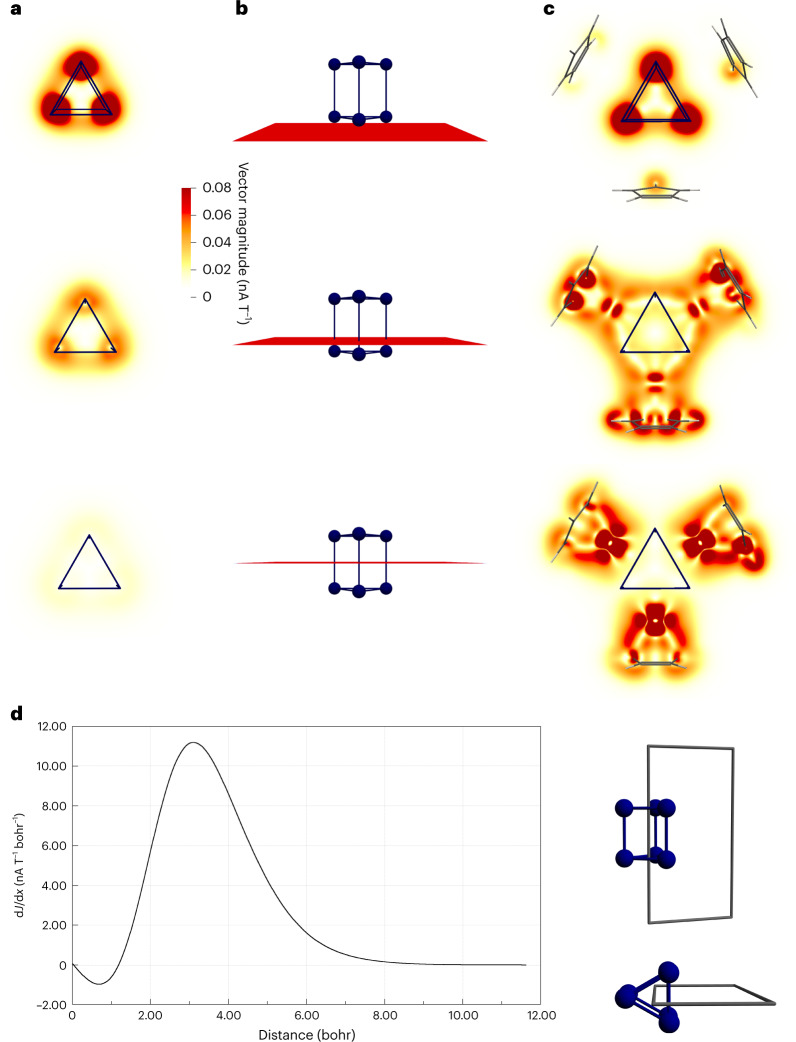
Illustration of the current density calculated for the model system Bi_6_^2–^ and for [1^calc^]^–^. **a**, Vector magnitude of magnetically induced current density, calculated for Bi_6_^2–^ in a plane 1 bohr below the lower {Bi_3_} unit (top), in a plane between the centre of mass of the {Bi_3_} unit and the global centre of mass (centre) and in a plane in the centre of the Bi_6_^2–^ prism (bottom). Note that the Bi atoms sustain a local ring current at the atom centre, which is evident by the red colour in the top and centre plots; this local ring current does not contribute to the net ring current flow of the cluster. The plot at the bottom indicates the presence of a ring current between the two {Bi_3_} triangles. **b**, Perpendicular view of the plots in **a** to illustrate their positions in the prism. **c**, Corresponding results calculated for **[1**^**calc**^**]**^**–**^. **d**, Current density profile of the ring current strength calculated for Bi_6_^2–^. The profile starts at the centre of the prism, and the Bi–Bi connection line is intersected at 1.65 bohr (inset at right). A positive sign indicates a diatropic contribution, whereas a negative sign indicates a paratropic contribution. d*J*/dx denotes the (numerical) derivative of the magnetically induced current density with respect to the distance x.

The nature of the φ-aromaticity was further corroborated and illustrated by the current profile and the current plot of the model system, which show that a small paratropic ring current is present inside the triangles, while a strong diatropic ring current is created on the outside (Fig. 6)—this ring current being a characteristic feature of aromatic compounds. Moreover, the plots of the magnetically induced current density illustrated in Fig. 6a,b clearly indicate a current flow through the complete Bi_6_^2–^ prism and not only in the two Bi triangles, in line with the non-localizable φ-type cluster orbital that exhibits a shape very similar to that of an atomic $f_{z^3}$ orbital. Matters are the same for **[1**^**calc**^**]**^**–**^ (Fig. 6c).

The spatial distribution of the current flow, that is, the occurrence of a substantial ring current above and below the triangles, is conceptually different from σ-aromaticity or *s*-type superatomic behaviour, which is predominantly observed for all-metal cages. It is similar to ‘classical’ π-aromaticity in that it lies above and below a planar ring; however, it is different as it occurs simultaneously in both of the triangular rings with the corresponding MO extending over all six atoms. According to the shape of the delocalized cluster orbitals both in the (hypothetical) homometallic cluster Bi_6_^2–^ and in its (experimentally confirmed) heteroatomic extension **1**^**–**^ (calculated as **[1**^**calc**^**]**^**–**^), we propose calling this type of aromaticity φ-aromatic.

According to the computational studies and the experimentally determined structures, the heteroatomic clusters **1**^**–**^ and **2**^**–**^, and the similar [{(CO)_3_Mo}_3_Bi_6_]^4−^, are clearly aromatic based on the magnetic criterion and sustain exceptionally strong diatropic ring currents according to the calculations. However, only the regular cluster **1**^**–**^ shows substantial signs of φ-aromaticity according to the symmetry criterion. Compared to the current density plot of Bi_6_^2–^, the clusters **1**^**–**^ and **2**^**–**^ show a reduced degree of σ-aromaticity. Particularly for **1**^**–**^, this can be rationalized by the nodal structure of the HOMO – 2 (MO 144a in Fig. 4). This MO corresponds to the lowest unoccupied MO of Bi_6_^2–^ (MO 71a in Fig. 4).

To compare this behaviour with that of iso(valence)electronic organic moieties, we computed the benzene isomer prismane (C_6_H_6_) and its hypothetical dianion (C_6_H_6_)^2–^, and analysed their respective electronic structures. In contrast to Bi_6_^2–^, the prismane dianion favours a triplet ground state by about 13 kJ mol^–1^. Prismane sustains a notable diatropic ring current of +19.2 nA T^–1^. This ring current is, however, completely localized in the two carbon triangles. The corresponding NICS values in these rings are –33.3 ppm, whereas the NICS values at the global centre of mass vanish. This ring current can be rationalized by the C–H and C–C bonds (Supplementary Table 8 and Supplementary Figs. 33 and 34), whose spatial counterparts at the carbon atoms lead to an accumulation of electron density in the carbon rings and above the carbon rings. Due to the spatial proximity of the carbon atoms, cyclic and delocalized orbital contributions are formed. Therefore, the ring current resembles the σ-type shape. A structurally related P-based compound, [{Cp*Fe}_3_(P_3_)_2_] (Cp*, C_5_Me_5_), showed a much more distinct separation of the two triangles, too^56^. This demonstrates clearly that heavy atoms are beneficial—arguably even required—for the phenomenon of φ-aromaticity.

## Conclusion

To summarize, we presented the synthesis of [K(crypt-222)]**1**·0.5tol and [K(crypt-222)]**2**·tol, which show excellent stability in solution and are synthetically reproducible in good yields. The heterometallic cluster anions in both compounds **1**^**–**^ and **2**^**–**^ are based on prismatic {Bi_6_} units, but these differ in structural details that cause different electronic structures. As rationalized by sophisticated quantum chemical investigations, including calculation of ring currents, we show that the Ru-based cluster **1**^**–**^, which is based on a very regular {Bi_6_} prism, displays a variant of aromatic behaviour that we introduce as φ-aromaticity. This involves two coplanar {Bi_3_} triangles within one non-localizable MO of $f_{z^3}$-like symmetry and a strong net diatropic ring current. Thorough inspection of the underlying Bi_6_^*q*–^ anions indicates that the Ru-based cluster behaves exactly like the species with two negative charges. The much more irregular Ir-based cluster **[2**^**calc**^**]**^**–**^, the calculated analogue of the experimental species **2**^**–**^, also shows a strong net diatropic ring current, but like the [{(CO)_3_Mo}_3_Bi_6_]^4−^ cluster reported recently^52^, displays weaker signs of φ-aromaticity owing to a substantially distorted core structure. A φ-type cluster orbital is also found for the distorted Bi_6_ prisms in these clusters, but this is by far less prominent than for the calculations on **1**^**–**^. The structural distortion observed in **2**^**–**^ is in accordance with the increased negative charge of the Bi_6_ core. We therefore conclude that the different metal complex fragments serve to stabilize different bonding types. This is in line with the nature of the two ligand types in terms of electron-donating or electron-withdrawing properties, as corroborated by natural bond orbital analyses.

The formation and isolation of compounds [K(crypt-222)]**1**·0.5tol and [K(crypt-222)]**2**·tol extends the concept of aromaticity upon the involvement of heavy metal atoms. We hope that the straightforward, convenient synthesis of these compounds will help efforts to gain greater understanding of the resulting properties and, in particular, the reactivity of metal aromatic species, as well as helping towards the design of other polycyclic metal clusters and towards further developing molecular bismuth chemistry.

## Methods

### General methods

All reactions were carried out under a dry, argon atmosphere using a standard Schlenk line or glove box techniques. Solvents were purified as appropriate: en (Aldrich, 99.8%) was refluxed over CaH_2_ for 24 hours, distilled and stored on 4 Å molecular sieves; DMF (Aldrich, 99.8%) and DMF-d_7_ (Sigma-Aldrich, 99.5%) were stored over 3 Å molecular sieves for five days, distilled under vacuum (~50 mbar) and stored on 4 Å molecular sieves; acetonitrile (VWR Chemicals, >99%) was stored over 3 Å molecular sieves for five days, distilled and stored on 3 Å molecular sieves; and *n*-hexane (Sigma-Aldrich, >95%) and toluene (Acros Organics) were refluxed over Na for 24 hours, distilled and stored on 4 Å molecular sieves. The crypt-222 (Kryptofix 222, Sigma-Aldrich) was dried under vacuum. The **A** was prepared by the published procedure on a larger scale (0.50 mmol)^44^. All other reagents were used as received: K (Acros Organics, 98%), Bi powder (ChemPur, 99.5%), [(cod)IrCl]_2_ (ChemPur) and [CpRu(MeCN)_3_][PF_6_] (Sigma-Aldrich). K_5_Bi_4_ was synthesized by combining K and Bi in stoichiometric amounts in a niobium ampoule. The ampoule was sealed by arc-welding, sealed in a quartz tube under vacuum, placed in an oven and kept at 700 °C for 7 days.

### Modified synthesis of A

K_5_Bi_4_ (0.515 g, 0.50 mmol) and crypt-222 (0.752 g, 2.00 mmol) were combined in a Schlenk tube and suspended in en (12.5 ml). The resultant dark green solution was stirred for 15 minutes, allowed to settle for 5 minutes and then filtered via a cannula fitted with a microfibre glass filter fixed with Teflon tape. The filtrate was first layered with toluene (37.5 ml) and then *n*-hexane (25 ml) and stored for 10 days in the dark. Large, dark red hexagonal crystals were filtered, washed with toluene (2 × 10 ml) and dried in vacuo affording **A** (0.743 g, 59.5%). Powder X-ray diffraction was used to determine the purity (Supplementary Fig. 35).

### Synthesis of [K(crypt-222)]1·0.5tol

The **A** (1.000 g, 0.80 mmol) and [CpRu(MeCN)_3_][PF_6_] (0.352 g, 0.80 mmol) were combined in a Schlenk tube and suspended in en (40 ml). The resultant brown mixture was stirred for 2 hours and filtered via a cannula fitted with a microfibre glass filter fixed with Teflon tape. The solvent was removed in vacuo and the residue was washed with acetonitrile (2 × 10 ml), affording [K(crypt-222)]**1**·0.5tol as a red-brown micro-crystalline powder (0.200 g, 35%). From the reaction stochiometry it is clear that (in addition to three equivalents of [PF_6_]^–^) another species has to balance the excess [K(crypt-222)]^+^ released upon reaction. No other products have been observed experimentally. Consequently, yields have been calculated with respect to the ratio written in the (non-stoichiometric) reaction scheme in equation (1), illustrating the formation of [K(crypt-222)]**1**·0.5tol:

1 $$\begin{array}{l}3\left[ {{{{\mathrm{K}}}}({{{{\mathrm{crypt}}}} \hbox{-} 222})} \right]_2{{{\mathrm{Bi}}}}_2 + 3\left[ {{{{\mathrm{CpRu}}}}\left( {{{{\mathrm{MeCN}}}}} \right)_3} \right]\left[ {{{{\mathrm{PF}}}}_6} \right] \\\to \left[ {{{{\mathrm{K}}}}\left( {{{{\mathrm{crypt}}}} \hbox{-} 222} \right)} \right]\left[ {\left\{ {{{{\mathrm{CpRu}}}}} \right\}_3{{{\mathrm{Bi}}}}_6} \right] + 5\left[ {{{{\mathrm{K}}}}\left( {{{{\mathrm{crypt}}}} \hbox{-} 222} \right)} \right]^ + + 3\left[ {{{{\mathrm{PF}}}}_6} \right]^- + 9\,{{{\mathrm{MeCN}}}}\end{array}$$

Crystals suitable for single-crystal X-ray crystallography were obtained on a smaller scale reaction (0.04 mmol) by layering after filtration of the reaction mixture with toluene (1 ml) and *n*-hexane (1 ml) and subsequent storage at 5 °C. The powder and crystals were confirmed to be the same by NMR spectroscopy. The ^1^H NMR (300.19 MHz, DMF-d_7_, 25 °C, Supplementary Fig. 6) is as follows: *δ* (in ppm) 5.21 (*s*, ^15^H, Cp), 3.63 (*s*, ^12^H, crypt-222), 3.59 (triplet, ^12^H, ^3^*J*_HH_ = 5 Hz, crypt-222) and 2.59 (triplet, ^12^H, ^3^*J*_HH_ = 5 Hz, crypt-222). The ^13^C{^1^H} NMR (75.49 MHz, DMF-d_7_, 25 °C, Supplementary Fig. 7) is as follows: *δ* (in ppm) 73.5 (Cp), 71.4 (crypt-222), 68.5 (crypt-222) and 54.9 (crypt-222).

### Synthesis of [K(crypt-222)]2·tol

The **A** (0.200 g, 0.16 mmol) and [Ir(cod)Cl]_2_ (0.054 g, 0.08 mmol) were combined in a Schlenk tube and suspended in en (8 ml). The resultant brown mixture was stirred for 2 hours and filtered via a cannula fitted with a microfibre glass filter fixed with Teflon tape. The solvent was removed in vacuo and the residue was washed with acetonitrile (2 × 10 ml), affording [K(crypt-222)]**2**·tol as a red-brown micro-crystalline powder (0.051 g, 58%). From the reaction stoichiometry it is clear that (in addition to three equivalents of Cl^–^) another species has to balance the excess [K(crypt-222)]^+^ released upon reaction. No other products have been observed experimentally. Consequently, yields have been calculated with respect to the ratio written in the (non-stoichiometric) reaction scheme in equation (2) illustrating the formation of [K(crypt-222)]**2**·tol:

2 $$\begin{array}{l}3\left[ {{{{\mathrm{K}}}}( {{{{\mathrm{crypt}}}} \hbox{-} 222} )} \right]_{{{\mathrm{2}}}}{{{\mathrm{Bi}}}}_2 + 3/2\left[ {{{{\mathrm{Ir}}}}\left( {{{{\mathrm{cod}}}}} \right){{{\mathrm{Cl}}}}} \right]_2 \\\to \left[ {{{{\mathrm{K}}}}( {{{{\mathrm{crypt}}}} \hbox{-} 222})} \right]\left[ {\left\{ {\left( {{{{\mathrm{cod}}}}} \right){{{\mathrm{Ir}}}}} \right\}_3{{{\mathrm{Bi}}}}_6} \right] + 5[{{{\mathrm{K}}}}({{{\mathrm{crypt}}}} \hbox{-} 222)]^ + + 3{{{\mathrm{Cl}}}}^-\end{array}$$

Crystals suitable for single-crystal X-ray crystallography were obtained on a smaller scale reaction (0.04 mmol) by layering after filtration of the reaction mixture with toluene (1 ml) and *n*-hexane (1 ml) and subsequent storage at 5 °C. The powder and crystals were confirmed to be the same by NMR spectroscopy. The ^1^H NMR (300.25 MHz, DMF-d_7_, 25 °C, Supplementary Fig. 8) is as follows: *δ* (in ppm) 4.44–4.43 (*m*, ^12^H, cod), 3.62 (*s*, ^12^H, crypt-222), 3.59 (triplet, ^3^*J*_HH_ = 5 Hz, ^12^H, crypt-222), 2.59 (triplet, ^3^*J*_HH_ = 5 Hz, ^12^H, crypt-222), 2.53–2.45 (*m*, ^12^H, cod) and 2.32–2.28 (*m*, ^12^H, cod). The ^13^C{^1^H} NMR (75.51 MHz, DMF-d_7_, 25 °C, Supplementary Fig. 9) is as follows: *δ* (in ppm) 71.4 (crypt-222), 68.6 (crypt-222), 54.9 (crypt-222), 53.2 (cod) and 36.7 (cod).

### Single-crystal X-ray diffraction data

The data for the X-ray structural analyses of compounds [K(crypt-222)]**1**·0.5tol and [K(crypt-222)]**2**·tol were collected at 100(2) K with Mo Kα radiation (wavelength, *λ* = 0.7107 Å) on an imaging plate detector system Stoe IPDS II using X-AREA v.1.88. The structures were solved by ShelXT15 (ref. ^58^). The refinement was done by full-matrix-least-squares methods against the square of the structure factors (*F*^2^) with the program ShelXL using Olex v.1.3.0 software^2^. The crystal data and experimental parameters of the structure determination are collected in Supplementary Table 1. Supplementary structural images are shown in Supplementary Figs. 1 and 2.

### Powder X-ray diffraction

The powder X-ray diffraction of **A** (Supplementary Fig. 35) was measured on a STOE StadiMP diffractometer system equipped with a Mythen 1 K silicon strip detector and a Cu Kα radiation source (*λ* = 1.54056 Å). An as-prepared sample of **A** was filled into a glass capillary (0.5 mm diameter), which was then sealed to be airtight with soft wax. The tube was then mounted onto the goniometer head using wax (horizontal set-up) and rotated throughout the measurements.

### Micro-X-ray fluorescence spectroscopy

All micro-X-ray fluorescence spectroscopy studies were performed with a Bruker M4 Tornado, equipped with an Rh-target X-ray tube and a silicon drift detector. The emitted fluorescence photons were detected with an acquisition time of 180 s. Quantification of the elements was achieved through deconvolution of the spectra. Results are shown in Supplementary Figs. 3 and 4. Note that for samples of Zintl cluster salts, the determination of the K content typically shows some deviations.

### Electrospray-ionization mass spectrometry

Electrospray-ionization mass spectra (Fig. 1a,b) were recorded with a Thermo Fischer Scientific Finnigan LTQ-FT spectrometer in the negative-ion mode. Single crystals of compounds [K(crypt-222)]**1**·0.5tol and [K(crypt-222)]**2**·tol were dissolved in freshly distilled DMF inside a glove box. In situ samples were taken from the reaction mixture and used directly (Supplementary Fig. 5). The solutions were ingested into the spectrometer with gastight 250 µl Hamilton syringes by syringe pump infusion. All capillaries within the system were washed with dry DMF or en before measurement.

### NMR spectroscopy

NMR measurements were recorded in J-Young NMR tubes on an AV III HD300 spectrometer using TopSpin3.2 in dried DMF-d_7_ (Supplementary Figs. 6–9**)**.

### Quantum chemical calculations

Calculations were performed with TURBOMOLE^59,60^, employing both small-core Dirac–Fock effective core potentials^61^ and the scalar-relativistic local exact two-component Hamiltonian^62–65^. For the effective core potential calculations, the dhf-TZVP basis set^66^ was used, whereas the x2c-TZVPall-s basis^65^ was employed in the exact two-component calculations^37,64^. The TPSS functional^67^ and the resolution of the identity approximation to the Coulomb integrals (RI-*J*) were used throughout^68^. Localized MOs were constructed with the Boys method^69^. Population analyses were performed according to Mulliken^70^ and the natural bond orbital method^55^. Orbital contributions were obtained with the Mulliken population analysis. The conductor-like screening model (COSMO) was applied with the default settings for all charged systems to simulate the counter ions^68,71^. Magnetically induced current densities were obtained with the GIMIC code^13,14^ using the perturbed density of TURBOMOLE^68^. More details are provided in Supplementary Section 6 (Supplementary Figs. 10–34 and Supplementary Tables 3–8). There, we also show the results with other density functional approximations including PBE0 (refs. ^72,73^), TPSSh (refs. ^67,74^) and ωB97X-D (ref. ^75^). For functionals depending on the kinetic energy density, we use both the generalization with the external magnetic field^76^ and with the paramagnetic current density^77,78^.

## Online content

Any methods, additional references, Nature Portfolio reporting summaries, source data, extended data, supplementary information, acknowledgements, peer review information; details of author contributions and competing interests; and statements of data and code availability are available at 10.1038/s41557-022-01099-5.

## Supplementary information

Supplementary Information Supplementary Figs. 1–35 and associated legends, Tables 1–8, Discussion on the formation of compounds [K(crypt-222)]**1**·0.5tol and [K(crypt-222)]**2**·tol, X-ray diffraction, micro-X-ray fluorescence spectroscopy, electrospray-ionization mass spectrometry, NMR spectrometry, quantum chemical investigations and powder X-ray diffractogram of the starting material **A**, and references.

Supplementary Data 1 CIF of compound 1 (CCDC 2157676).

Supplementary Data 2 CIF of compound 2 (CCDC 2157677).

Supplementary Data 3 Cartesian coordinates of all calculated structures (ASCII format).

Supplementary Data 4 Source data for the current height profiles displayed in Supplementary Figs. 19–24.

Supplementary Data 5 Source data for the current distance profiles displayed in Supplementary Figs. 23, 24 and 33.

## Data Availability

All data generated or analysed during this study are included in this published Article and its Supplementary Information files. The structures of compounds [K(crypt-222)]**1**·0.5tol and [K(crypt-222)]**2**·tol were determined by single-crystal X-ray diffraction. The crystallographic data for the two structures reported in this paper have been deposited with the Cambridge Crystallographic Data Centre (CCDC) as supplementary publication nos CCDC 2157676 ([K(crypt-222)]**1**·0.5tol) and CCDC 2157677 ([K(crypt-222)]**2**·tol). The Cartesian coordinates of all optimized structures and the respective self-consistent field (SCF) energies are summarized in the supplementary document ‘optimized-structures.txt’. The files comprise all necessary data for reproducing the values. All non-default parameters for the computational studies are given in the Supplementary Information together with the corresponding references of the methods used. For the default parameters of TURBOMOLE, such as the convergence criteria for structure optimizations, please see the manual at https://www.turbomole.org (retrieved 12 February 2022). Further details are provided in the Supplementary Information.

## Code availability

The TURBOMOLE quantum program suite is available from https://www.turbomole.org (retrieved 12 February 2022), and the GIMIC code (version 2.1.4 commit 3a5f0eb 2019) can be obtained from the GitHub repository at https://github.com/qmcurrents/gimic (retrieved 29 August 2020; open source; information in refs. ^13,14^). The GitHub repository also includes sample input for GIMIC.

As an additional note on the GIMIC code and its TURBOMOLE interface with Python version 2, the version of GIMIC used here uses Python version 3. When using Python version 2, the line ‘from__future__import print_function’ must be added to the header of the build version of turbo2gimic.py, which converts TURBOMOLE’s (un)perturbed density matrices, structure and basis set data to the GIMIC input. This allows Python versions 2.6 and 2.7 to interpret the Python version 3 syntax of the print function.
