# Supplementary material for: φ-Aromaticity in prismatic {Bi6}-based clusters
Source: Nat Chem. 2022 Dec 22;15(3):347–56. doi: 10.1038/s41557-022-01099-5 (PMC9986111; doi:10.1038/s41557-022-01099-5)
Supplement: Supplementary file 1 — Supplementary Figs. 1–35 and associated legends, Tables 1–8, Discussion on the formation of compounds [K(crypt-222)]1·0.5tol and [K(crypt-222)]2·tol, X-ray diffraction, micro-X-ray fluorescence spectroscopy, electrospray-ionization mass spectrometry, NMR spectrometry, quantum chemical investigations and powder X-ray diffractogram of the starting material A, and references. [file 41557_2022_1099_MOESM1_ESM.pdf]

nature chemistry

Article https://doi.org/10.1038/s41557-022-01099-5

φ-Aromaticity in prismatic $\{Bi_6\}$-based clusters

In the format provided by the authors and unedited

Supplementary Information

φ-Aromaticity in prismatic {Bi_6}-based clusters

Benjamin Peerless,[1,2] Andreas Schmidt,[1,2] Yannick J. Franzke,*[1] and Stefanie Dehnen*[1,2]

[1] Fachbereich Chemie, Philipps-Universität Marburg, Hans-Meerwein-Str. 4, 35032 Marburg, Germany.
[2] Current address: Karlsruhe Institute of Technology (KIT), Institute of Nanotechnology, Hermann-von-Helmholtz-Platz 1, 76344 Eggenstein-Leopoldshafen, Germany. Corresponding Authors E-Mail: stefanie.dehnen@kit.edu; yannick.franzke@chemie.uni-marburg.de.

Contents

1. Supplementary Discussion on the Formation of Compounds [K(crypt-222)]1·0.5tol and [K(crypt-222)]2·tol 3

2. Supplementary Information on X-Ray Diffraction 4

2.2. Supplementary Information on the Packing of the Molecules in the Unit Cells of [K(crypt-222)]1·0.5tol and [K(crypt-222)]2·tol 5

2.3. Responses to B-Level Alerts in CIFs 7

2.3.1. Responses to B-Level Alerts in the CIF of [K(crypt-222)]1·0.5tol 7

2.3.2. Responses to B-Level Alerts in the CIF of [K(crypt-222)]2·tol 7

3. Supplementary Information on Micro-X-Ray Fluorescence Spectroscopy (μ-XFS) 8

4. Supplementary Information on Electrospray Ionization (ESI) Mass Spectrometry 10

5. Supplementary Information on nuclear magnetic resonance (NMR) Spectrometry 11

5.1. ^{1}H-NMR and ^{13}C{^{1}H}-NMR Spectra of [K(crypt-222)][{CpRu}$_3$Bi$_6$]·0.5tol 11

5.2. ^{1}H-NMR and ^{13}C{^{1}H}-NMR Spectra of [K(crypt-222)][{(cod)Ir}$_3$Bi$_6$]·tol 13

6. Supplementary Details on Quantum Chemical Investigations 15

6.1. Computational Methods and Details 15

6.2. Canonical Frontier Molecular Orbitals 16

6.3. Localized Molecular Orbitals of Bi_6^{2-} 18

6.4. Current Strengths and NICS of Bi_6^{q-} Clusters and the Ru/Ir-Based Compounds 19

6.5. Current Profiles with Respect to the Height 25

6.6. Current Distance Profiles of Bi_6^{2-} and Bi_6^{4-} 28

6.7. Current Density Plot of Bi_6 29

6.8. Current Density Plot of Bi_6^{2-} 30

6.9. Current Density Plot of Bi_6^{4-} 31

6.10. Current Density Plot of 1^{-} 32

6.11. Current Density Plot of 2^{-} 33

6.12. Comparison to [{(CO)$_3$Mo}$_3$Bi$_6$]$^{4-}$ 34

6.13. Current Strengths of Prismane 37

7. Powder X-Ray Diffractogram of the Starting Material [K(crypt-222)]$_2$Bi$_2$ (A) 39

8. References for the Supplementary Information 40

1. Supplementary Discussion on the Formation of Compounds [K(crypt-222)]**1**·0.5tol and [K(crypt-222)]**2**·tol

The formation pathway of the anions **1**$^-$ and **2**$^-$ apparently follows a complicated redox cascade, as at some stage the Bi_2^{2-} has to undergo a number of oxidation processes to account for the surprisingly low charge of the overall clusters in compounds [K(crypt-222)]**1**·0.5tol and [K(crypt-222)]**2**·tol.

Regarding the prismatic architecture of the {Bi_6} in $[\{LM\}_3Bi_6]^-$ (LM = CpRu in **1** and (cod)Ir in **2**) with a formal charge of 4– in combination with three formally 1+ charged {LM} units, two pathways seem to be plausible starting out from Bi_2^{2-} based on observations made in this study and in previous ones:

(1) Formation of two 6π-aromatic species Bi_3^{3-} from three Bi_2^{2-} in the first step, potentially/in part with transition metal complex fragments attached, and a pairwise coplanar oxidative coupling.

(2) Formation of the known anion Bi_4^{2-} (under 2-e$^-$ oxidation) and linkage of the latter with the originally provided Bi_2^{2-} anion under assistance and attachment of transition metal complex fragments.

The formation of Bi_4^{2-} was reported in previous studies. The compound can be identified easily from the formation of a blueish-greenish color of the reaction solution shortly upon combination of the reactants. However, in the current case, such observations were not made, which seems to disfavor route (2).

Notably, a species $[\{(cod)Ir\}_3Bi_3H]^-$ is observed in the ESI-MS studies of the reaction solution yielding the cluster **1**$^-$. A plausible reaction scheme for the final cluster thus accords with pathway (1) from the experimental data. We assume that upon combination of a Bi_3^{3-} unit with three $[(cod)Ir]^+$ fragments, this species actually carries no charge in solution, but takes up one negative charge during transfer into the gas phase in the ESI-MS experiment. Although a corresponding experimental proof is missing in the case of cluster **2**$^-$ as no other products have been experimentally identifiable (in agreement with the differently Lewis-acidic influence of the transition metal fragments), this does not exclude a corresponding mechanism to occur.

We therefore suggest the formation of the cluster via triangular units, their intermediate stabilization (and charge reduction) by attachment of three transition metal complex fragments, and subsequent combination of this heterometallic, hexanuclear cluster with another Bi_3^{3-} moiety under 2-e$^-$ oxidation. An alternative scenario would include the preceding oxidation of Bi_3^{3-} to give Bi_3^- and a combination of the latter with the {Ir_3Bi_3}-based unit, yet this seems to be less likely, as Bi_3^- is an antiaromatic species and its formation is therefore less favorable.

The questions to be answered in future work addresses the details of the oxidation step. Based on preliminary findings, we suggest a concerted activity of the named intermediates with lower-charge polybismuthide-units that are further reduced to form higher-charge polybismuthide anions as by-products. Given that we can obtain compounds [K(crypt-222)]**1**·0.5tol and [K(crypt-222)]**2**·tol in appreciable quantities, we suggest that their formation represents the first steps towards further investigation of the chemistry and reactivity of Bi-rich compounds in general.

2. Supplementary Information on X-Ray Diffraction

All hydrogen atoms were kept riding on calculated positions with isotropic displacement parameters U = 1.2 U_{eq} (or 1.5 U_{eq} for methyl groups) of the bonding partners. Crystallographic data for the two structures reported in this paper have been deposited with the Cambridge Crystallographic Data Centre as supplementary publications nos. CCDC-2157676 ([K(crypt-222)]**1**·0.5tol) and CCDC-2157677 ([K(crypt-222)]**2**·tol). The crystal data and experimental parameters of the structure determinations are collected in **Supplementary Table S1**. Supplementary structural figures are provided in **Supplementary Figure 1** and **Supplementary Figure 2**.

2.1. Supplementary Information on the Single-Crystal X-Ray Diffraction Studies

Supplementary Table 1: Crystal data and details of the structure determinations of [K(crypt-222)]1·0.5tol and [K(crypt-222)]2·tol.

**Compound**	**[K(crypt-222)]1·0.5tol**	**[K(crypt-222)]2·tol**
empirical formula	$C_{153}H_{198}Bi_{24}K_4N_8O_{24}Ru_{12}$	$C_{98}H_{160}Bi_{12}Ir_6K_2N_4O_{12}$
nominal formula	$C_{153}H_{198}Bi_{24}K_4N_8O_{24}Ru_{12}$	$C_{98}H_{160}Bi_{12}Ir_6K_2N_4O_{12}$
formula weight [g mol^{-1}]	8856.05	5324.44
crystal colour, shape	brown, plate	brown, block
crystal size [mm^3]	0.05×0.19×0.20	0.03×0.05×0.11
crystal system	triclinic	triclinic
space group	$P\acute{1}$	$P\acute{1}$
a [Å]	12.8292(6)	13.8451(5)
b [Å]	14.6718(7)	14.0517(5)
c [Å]	29.3530(12)	30.9905(11)
α [°]	92.671(4)	86.264(3)
α [°]	96.597(3)	78.263(3)
γ [°]	112.370(3)	82.948(3)
V [Å^3]	5050.8(4)	5853.4(4)
Z / ρ_{calc} [g cm^{-3}]	1 / 2.912	2 / 3.021
μ [mm^{-1}]	($Mo_{K\alpha}$) 21.82	($Mo_{K\alpha}$) 24.86
absorption correction	numerical	numerical
Θ range [°]	2.8 – 53.6	2.7 – 53.6
total reflns	60708	74371
unique reflns / [R_{int}]	21412 / 0.0736	24754 / 0.0562
obs. Reflns [$I > 2\sigma(I)$]	21412	24754
Parameters	853	1209
wR_2 (all data) / R_1 [$I > 2\sigma(I)$]	0.1754 / 0.0628	0.0560 / 0.0296
GooF (all data)	0.97	0.91
max peak/hole [e Å^{-3}]	2.42 / -3.60	2.05 / −0.95
CCDC number	2157676	2157677

2.2. Supplementary Information on the Packing of the Molecules in the Unit Cells of [K(crypt-222)]1·0.5tol and [K(crypt-222)]2·tol

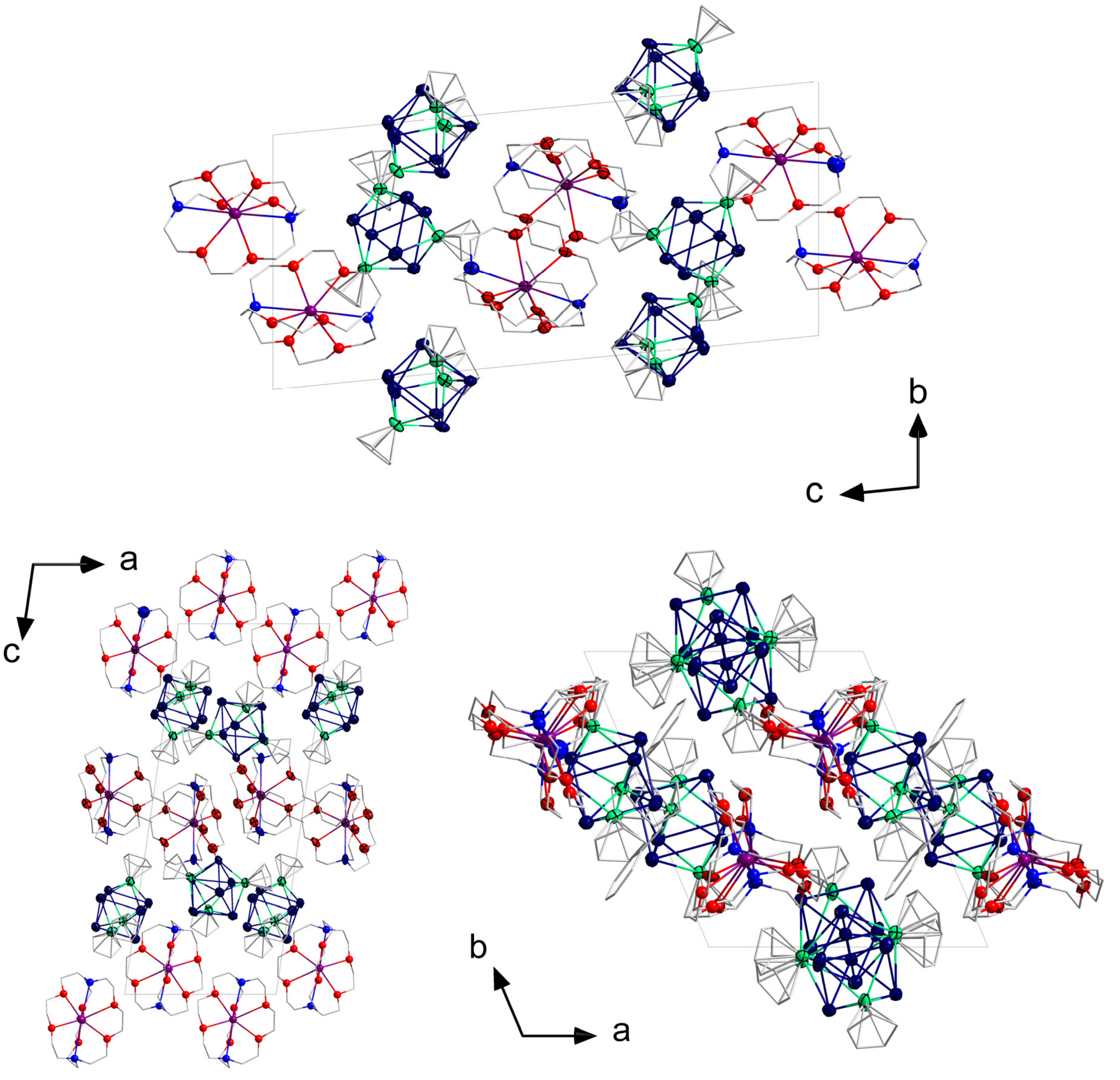

Supplementary Figure 1: Views of the unit cell of the crystal structure of [K(crypt-222)]1·0.5tol along crystallographic *a*, *b*, and *c* axes. Thermal displacement ellipsoids are presented with a 50% probability. Two $[K(crypt\text{-}222)]^+$ cations could not be refined using anisotropic displacement parameters and are therefore presented in ball-and-stick mode. Atom color code within cluster anions: Bi – blue, Ru – green. Atom color code within $[crypt\text{-}222]^+$ cations: K – purple, O – red, N – blue, C – grey (wires). H atoms are omitted.

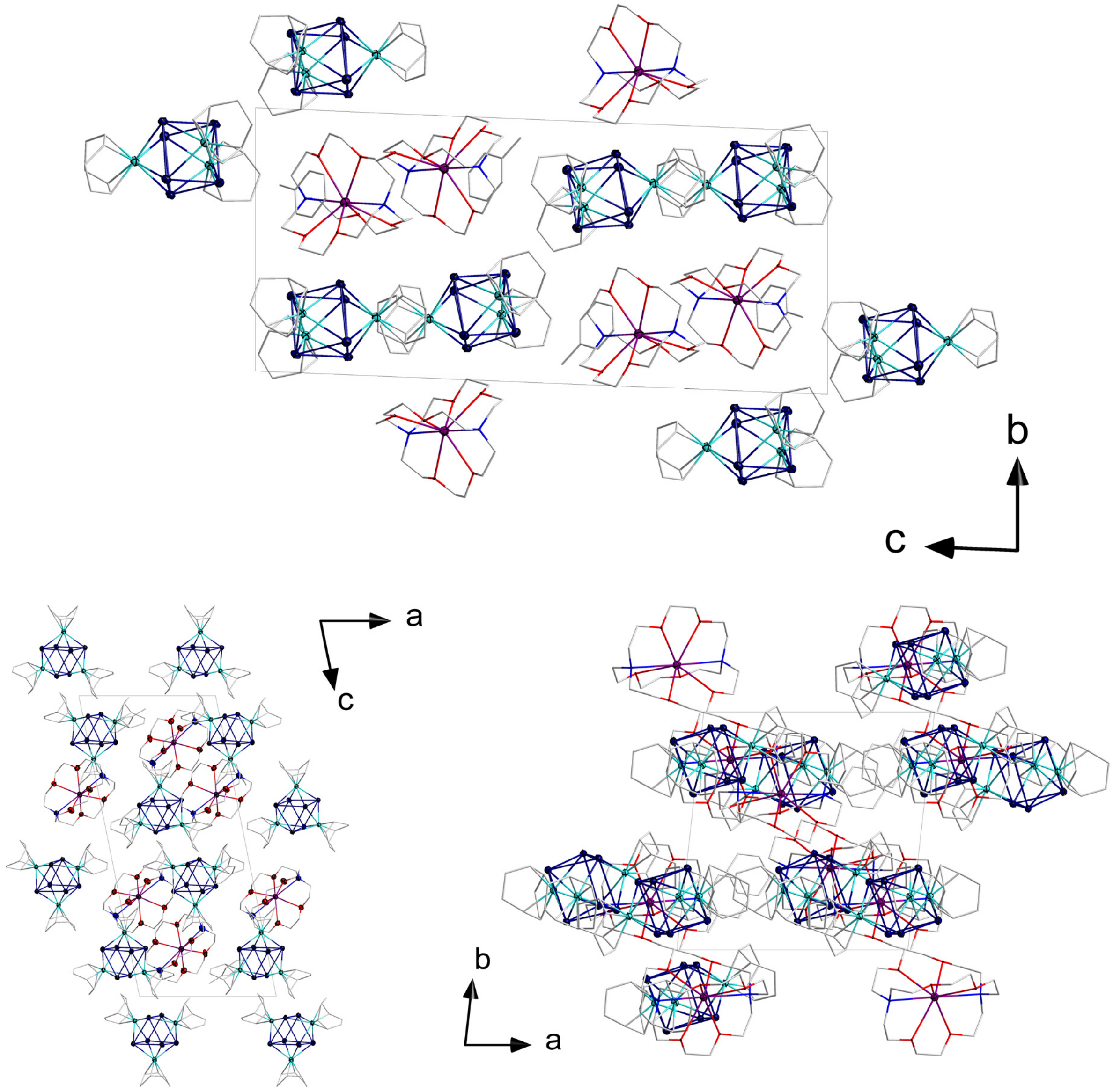

Supplementary Figure 2: Views of the unit cell of the crystal structure of [K(crypt-222)]2·tol along crystallographic *a*, *b*, and *c* axes. Thermal displacement ellipsoids are presented with a 50% probability. Atom color code within cluster anions: Bi – blue, Ru – green. Atom color code within $[crypt\text{-}222]^{+}$ cations: K – purple, O – red, N – blue, C – grey (wires). H atoms are omitted.

2.3. Responses to B-Level Alerts in CIFs

2.3.1. Responses to B-Level Alerts in the CIF of [K(crypt-222)]1·0.5tol

PROBLEM: High 'MainMol' Ueq as Compared to Neighbors of O11_1 Check
RESPONSE: This is due to the fact that one of the crypt-222 molecules has been fixed using the Fragment DB tool in Olex^{2}v1.3.0 software.

PROBLEM: Low 'MainMol' Ueq as Compared to Neighbors of K2 Check
RESPONSE: This is due to the fact that one of the crypt-222 molecules has been fixed using the Fragment DB tool in Olex^{2}v1.3.0 software.

PROBLEM: Low Bond Precision on C-C Bonds .... 0.03036 Ang.
RESPONSE: This is due to the fact that one of the crypt-222 molecules has been fixed using the Fragment DB tool in Olex^{2}v1.3.0 software.

PROBLEM: Short Intra H…H Contact H62A_1 ..H55A_1. 1.81 Ang. x,y,z = 1_555 Check
RESPONSE: This is due to the fact that one of the crypt-222 molecules has been fixed using the Fragment DB tool in Olex^{2}v1.3.0 software.

2.3.2. Responses to B-Level Alerts in the CIF of [K(crypt-222)]2·tol

PROBLEM: High 'MainMol' Ueq as Compared to Neighbors of C46 Check
RESPONSE: This is due to some disorder of the atoms in the respective crypt-222 molecule, for which a split model did not serve to produce better results.

3. Supplementary Information on Micro-X-Ray Fluorescence Spectroscopy (μ-XFS)

Results of the μ-XFS measurements are summarized in **Supplementary Table 2**, corresponding spectra are shown in **Supplementary Figure 3** and Supplementary Figure 4**.** The data of both compounds were collected on crystals from the same reaction the crystal structures were obtained from.

Several measurements produced a deviation of the K versus Bi amounts. This is frequently observed for air-sensitive compounds, and also affects the data obtained for the other elements. We assume that this is due to beginning corrosion on the crystal surface upon exposure during sample preparation.

The appearance of Rh in the measured spectrum is a consequence of the Rh X-ray source.

Supplementary Table 2: μ-XFS analysis of [K(crypt-222)]1·0.5tol (K, Ru, Bi) and [K(crypt-222)]2·tol (K, Ir, Bi).

Element	Element wt %	Weight % err. (1 sigma)	Atom %	Atom % calc.	Absolute deviation.
			([K(crypt-222)]**1**·0.5tol)		
K-K	3.83	0.01	14.85	10.00	+48.5%
Ru-L	19.83	0.01	29.76	30.00	-0.08%
Bi-L	76.34	0.01	55.39	60.00	-7.68%
Total	100.00		100.00	100.00	
			([K(crypt-222)]**2**·tol)		
K-K	2.34	0.01	11.07	10.00	+9.67%
Ir-L	31.62	0.01	30.45	30.00	+1,48%
Bi-L	66.04	0.01	58.49	60.00	-2.52%
Total	100.00		100.01	100.00	

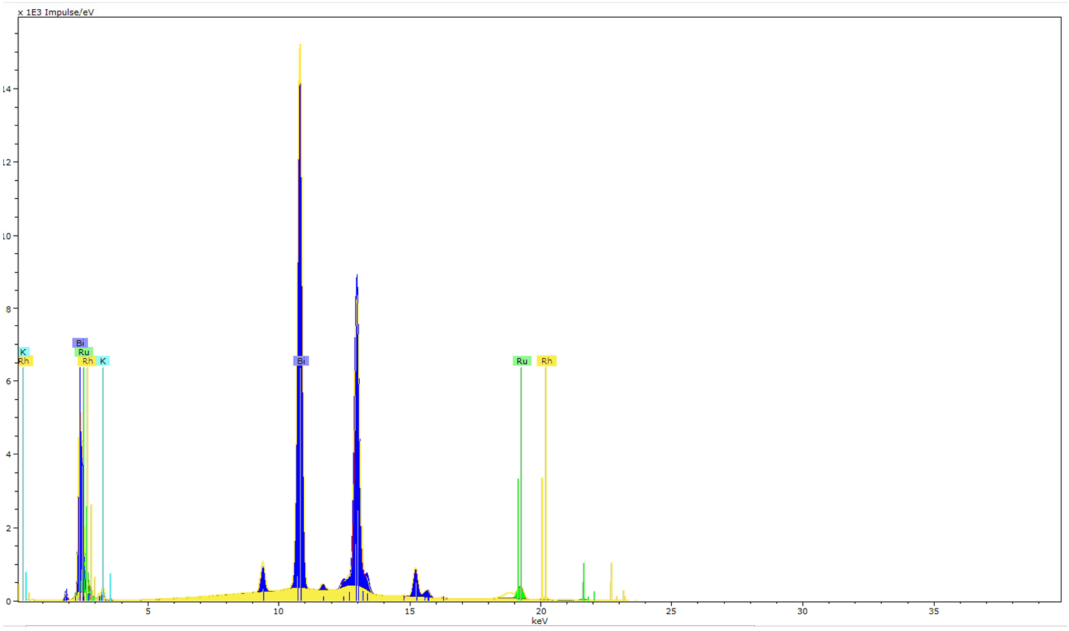

Supplementary Figure 3: ***µ*-XFS spectrum of [K(crypt-222)]1·0.5tol (line) with the results of the deconvolution algorithm (solid, colored).** Colors are used as follows: K (turquoise), Bi (blue), Ru (green). Note, that the intensity is displayed on a square root scale to allow for a better visibility of small features in the spectrum.

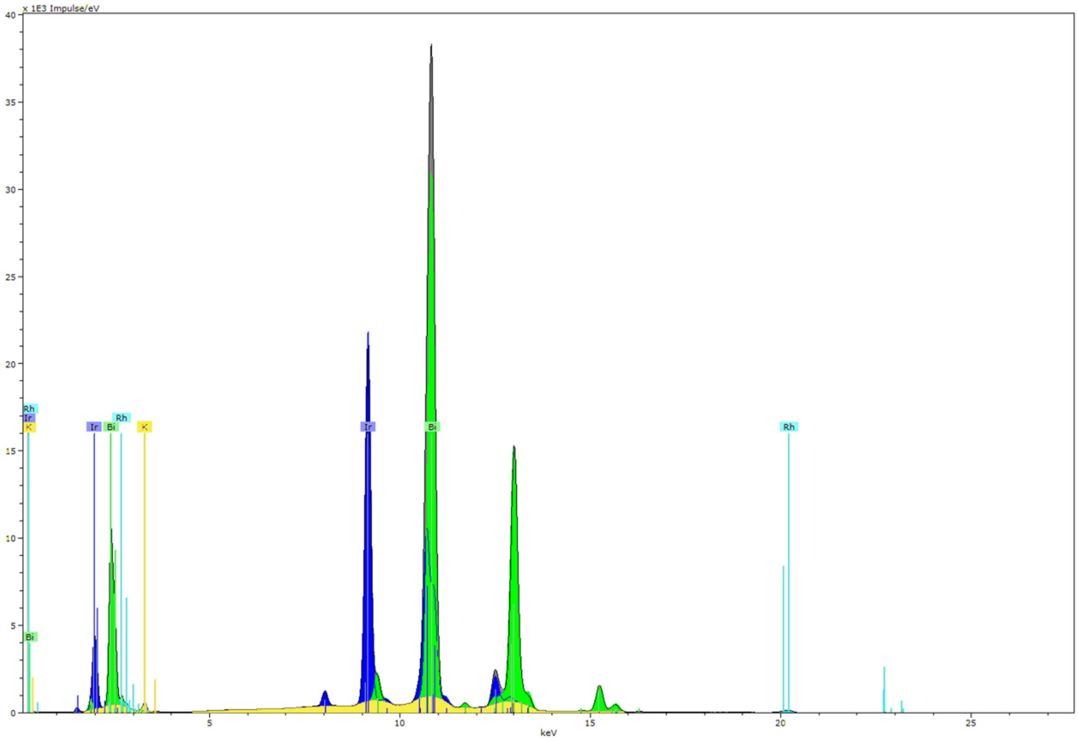

Supplementary Figure 4: *µ*-XFS spectrum of [K(crypt-222)]2·tol (line) with the results of the deconvolution algorithm (solid, colored). Colors are used as follows: K (orange), Bi (green), Ir (blue). Note, that the intensity is displayed on a square root scale to allow for a better visibility of small features in the spectrum.

4. Supplementary Information on Electrospray Ionization (ESI) Mass Spectrometry

Results of the *in-situ* ESI-MS measurement of a mixture of $[K(crypt\text{-}222)]_2Bi_2$ and $[(cod)IrCl]_2$ in ethane-1,2-diamine is shown in **Supplementary Figure 5**.

4.1. *In-situ* ESI mass spectrum of a mixture of $[K(crypt\text{-}222)]_2Bi_2$ and $[(cod)IrCl]_2$ in ethane-1,2-diamine

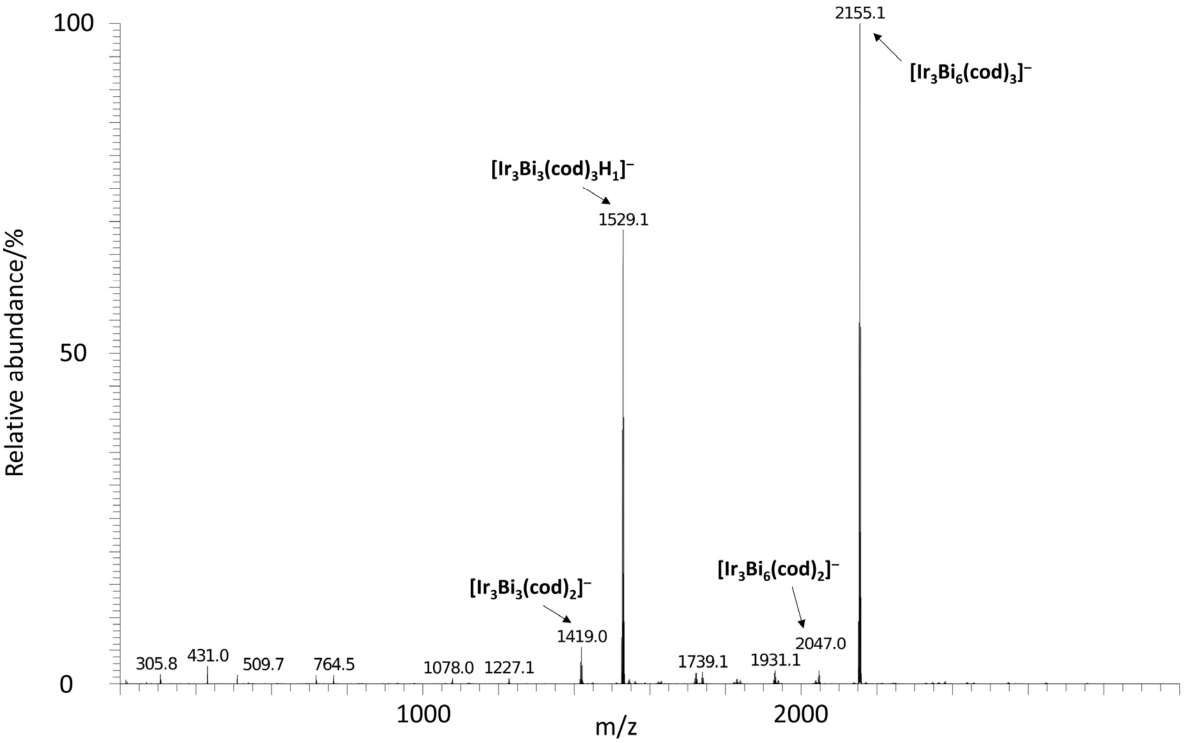

Supplementary Figure 5: High resolution *in-situ* ESI mass spectrum in negative ion mode of a reactive solution of $[K(crypt\text{-}222)]_2Bi_2$ and $[(cod)IrCl]_2$ in ethane-1,2-diamine. Overview spectrum between 200 and 3000 m/z with assigning of signals with a relative abundance higher than 5%. While the species at m/z = 1529.1 did not form an isolable product, single-crystals of [K(crypt-222)]**2**·tol were obtained from this solution that produce a pure mass spectrum of the peak at m/z = 2155.1 when re-dissolved in freshly distilled DMF (see main document).

5. Supplementary Information on nuclear magnetic resonance (NMR) Spectrometry

^{1}H-NMR and $^{13}C\{^{1}H\}$-NMR spectra recorded from solutions of single-crystals of compounds [K(crypt-222)]**1**·0.5tol and [K(crypt-222)]**2**·tol are provided in **Supplementary Figures 6 –9**.

5.1. ^{1}H-NMR and ^{13}C{^{1}H}-NMR Spectra of [K(crypt-222)][{CpRu}$_3$Bi$_6$]·0.5tol

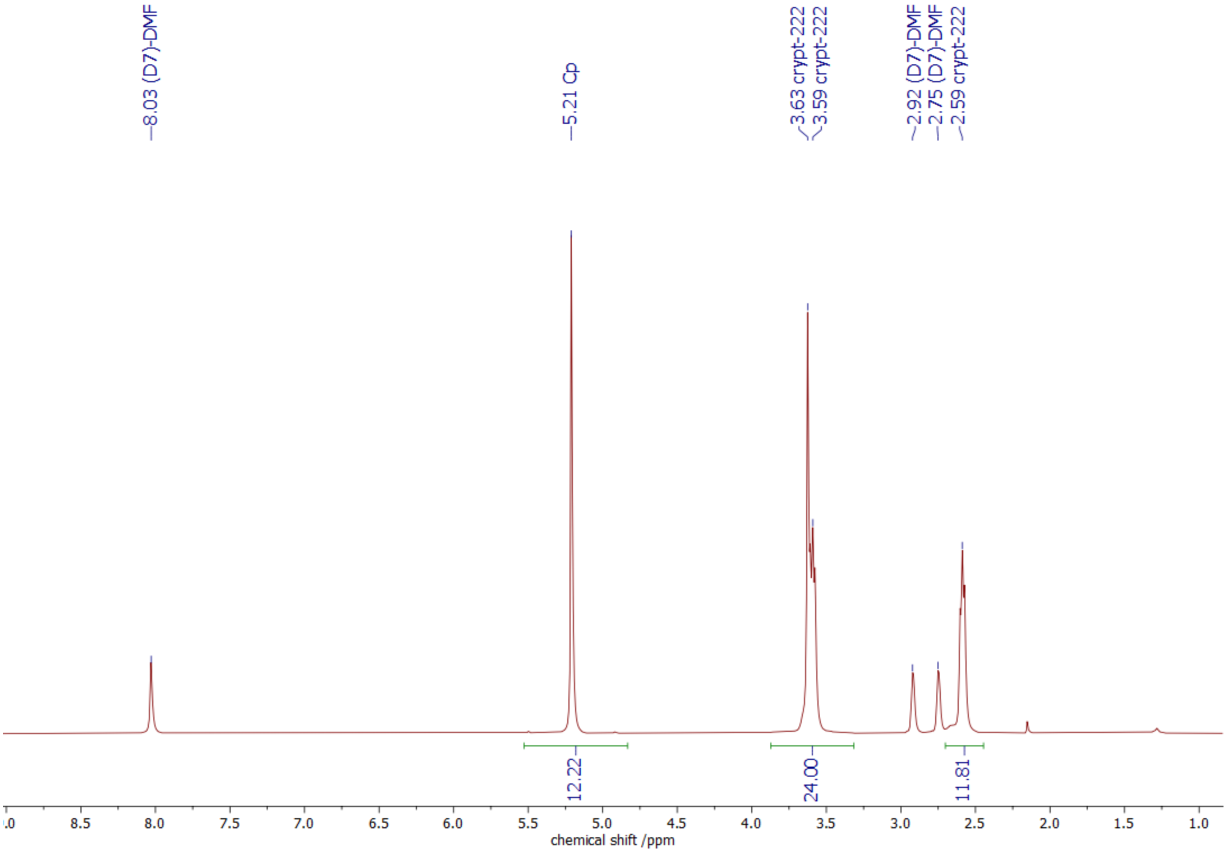

Supplementary Figure 6: ^{1}H-NMR spectrum of [K(crypt-222)]**1**·0.5tol in (D$_7$)-DMF.

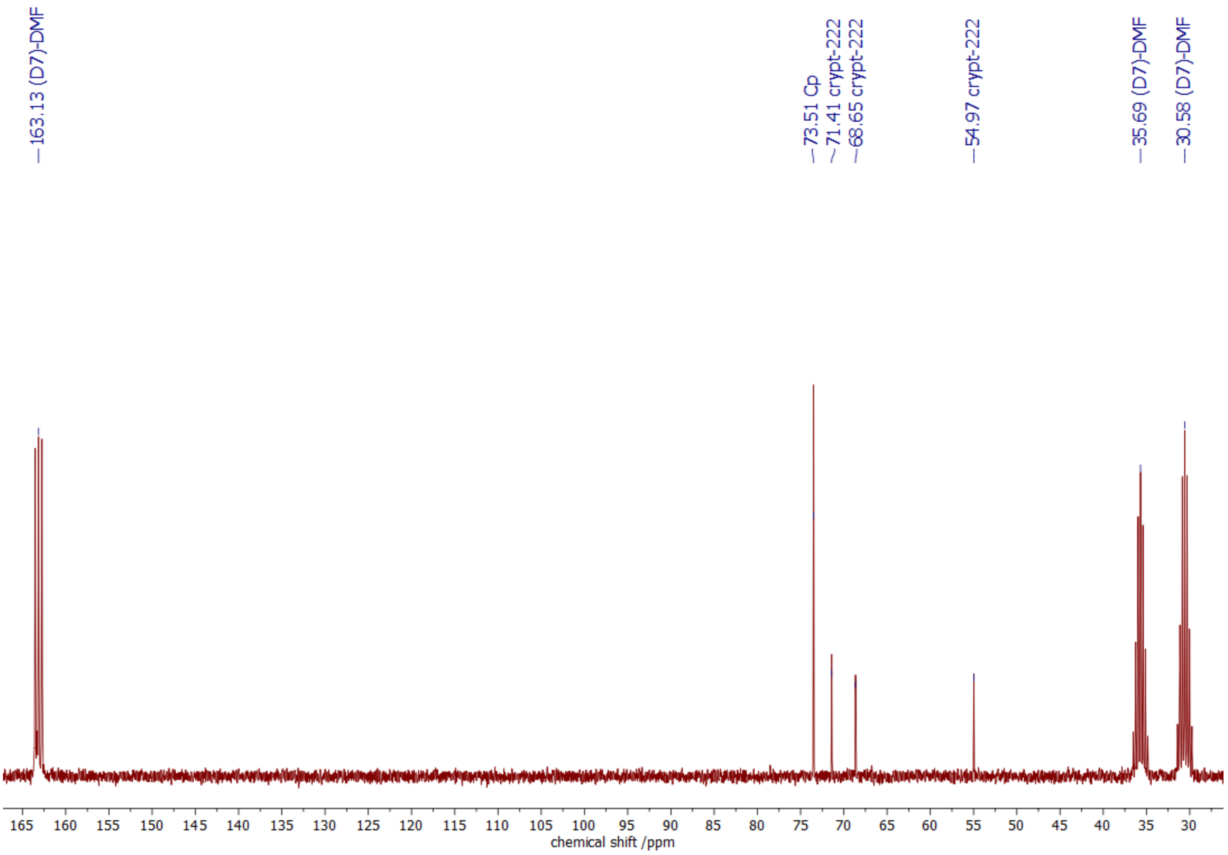

Supplementary Figure 7: $^{13}C\{^{1}H\}$-NMR spectrum of [K(crypt-222)]**1**·0.5tol in (D_7)-DMF.

5.2. ¹H-NMR and $^{13}C\{^1H\}$-NMR Spectra of [K(crypt-222)][{(cod)Ir}$_3$Bi$_6$]·tol

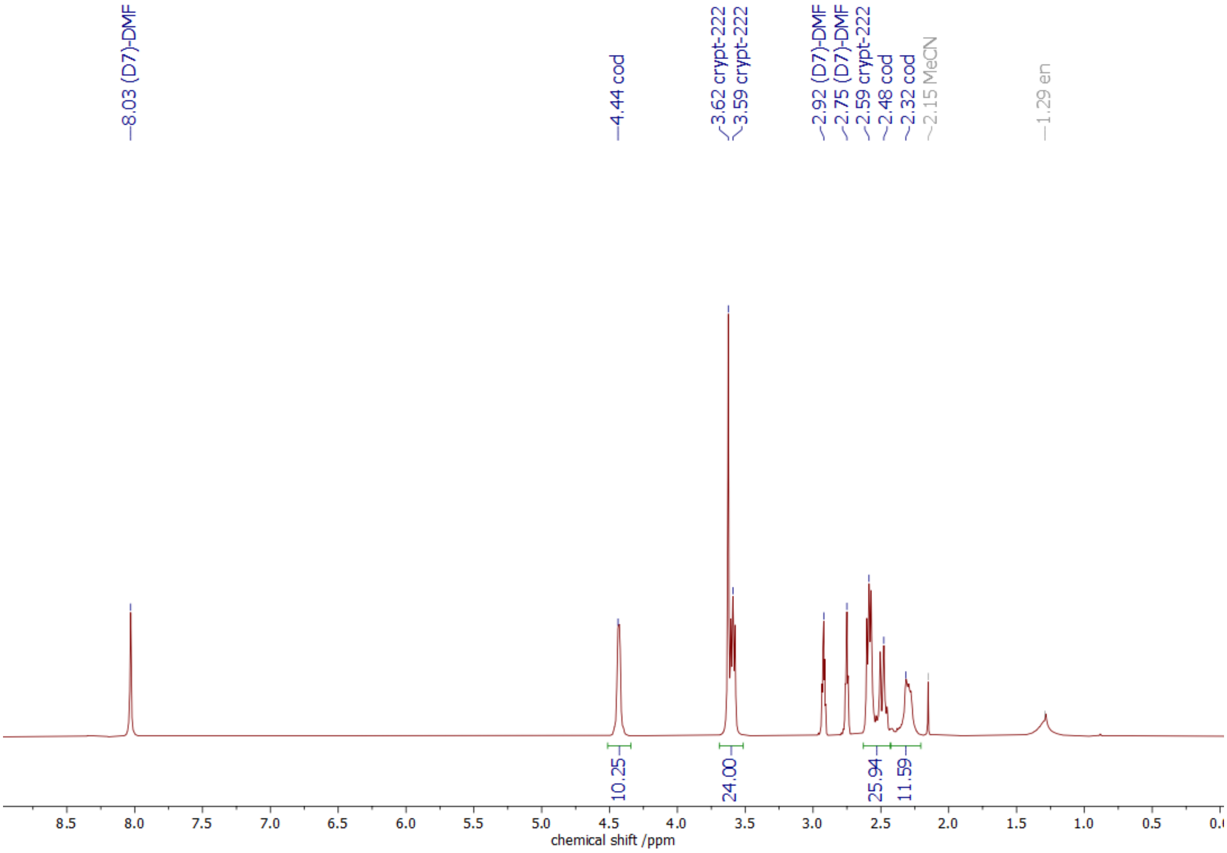

Supplementary Figure 8: ¹H-NMR spectrum of [K(crypt-222)]**2**·tol in (D$_7$)-DMF.

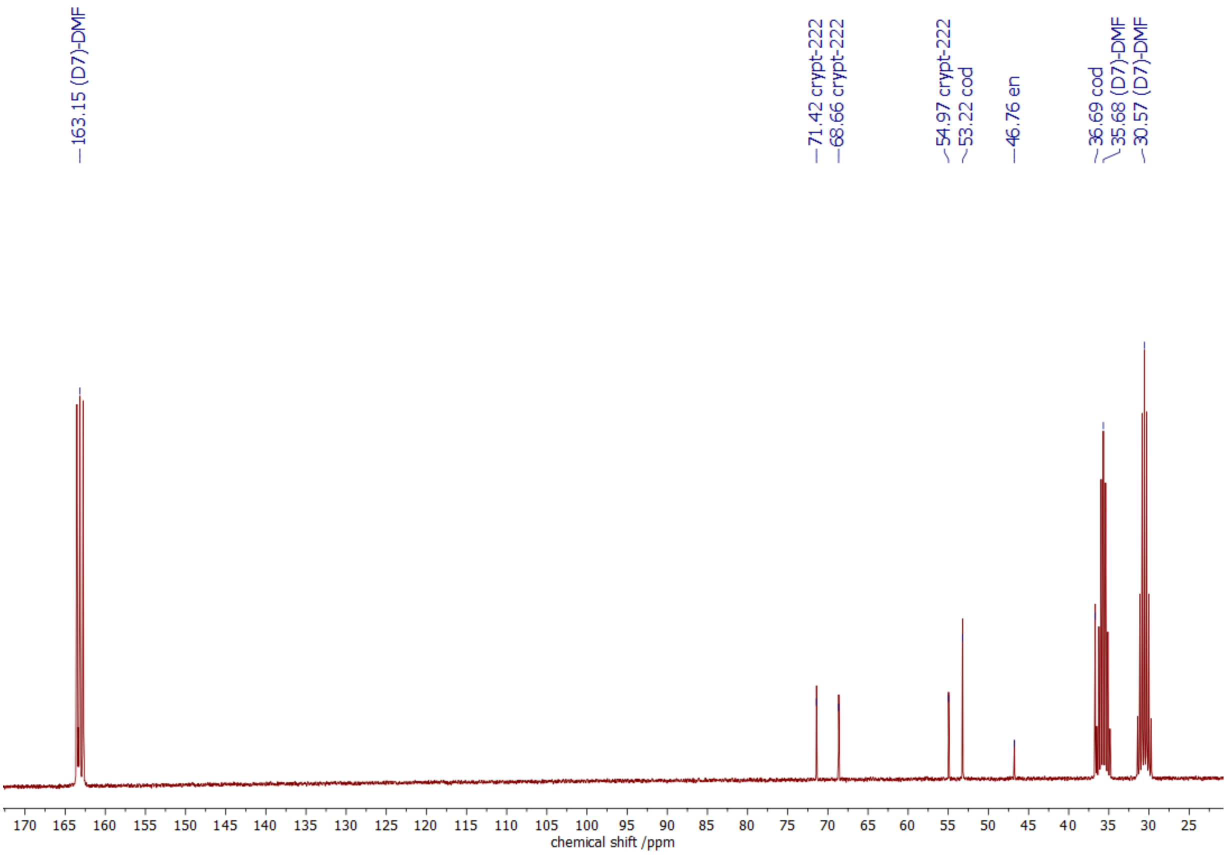

Supplementary Figure 9: $^{13}C\{^{1}H\}$-NMR spectrum of [K(crypt-222)]**2**·tol in (D_7)-DMF.

6. Supplementary Details on Quantum Chemical Investigations

6.1. Computational Methods and Details

Quantum chemical calculations were carried out with the TURBOMOLE program suite.[1-4] Structures were optimized with the TPSS density functional approximation[5] and the dhf-TZVP basis set[6] employing small-core Dirac–Fock effective core potentials (ECPs) for heavy elements.[7] In detail, an ECP-28 was applied for Ru, and an ECP-60 for Ir and Bi. The resolution of the identity approximation to the Coulomb integrals (RI-*J*) was employed with tailored auxiliary basis sets.[6] Fine grids were used for the numerical integration of the exchange-correlation terms (grid size 3a).[8-10] COSMO[11,12] was applied with the default parameters to compensate the negative charge and to model the counter ions. Thresholds of 10^{-8} E_h for the energy and 10^{-7} a.u. for the root mean square of the density matrix norm were chosen in the self-consistent field (SCF) procedure. For the structure optimizations, thresholds of 10^{-6} E_h for the energy and 10^{-3} E_h/bohr for the maximum gradient indicated convergence of the structure optimizations. The Cartesian coordinates of all optimized structures and the respective SCF energies are summarized in the supplementary document "optimized-structures.txt". The convergence of the ground-state configurations was validated with fractional occupation numbers and large damping factors of 5–8 a.u. during the SCF iterations. Orbitals were localized with the Boys method.[13] The chemical bonding was also studied with a self-consistent two-component formalism to treat spin–orbit coupling.[14] However, spin–orbit coupling is of minor importance for the bonding situation and does not lead to qualitative changes for the studied molecules.

For the NMR shielding calculations,[15,16] the kinetic-energy density was generalized with the vector potential of the magnetic field[15,17] (TPSS) or the paramagnetic current density (cTPSS).[18-23] NMR calculations were carried out with both ECPs and the all-electron scalar-relativistic exact two-component (X2C) Hamiltonian in its local approximation (DLU-X2C).[24-26] The x2c-TZVPall-s basis sets[10] were used for the latter. A threshold of 10^{-7} a.u. for the norm of the residuum was applied to indicate the convergence of the coupled-perturbed Kohn–Sham (CPKS) equations. Nucleus-independent chemical shifts[27] (NICS) were calculated at the center of mass of the complete molecule and the respective triangular units. The calculations are also corroborated with other density functional approximations, see Section 6.4.

Magnetically induced current densities and current strengths were obtained with the gauge-including magnetically induced current (GIMIC) method.[28,29] This method uses the SCF ground-state density matrix and the CPKS perturbed density matrix of an NMR shielding calculation. The grids for the magnetically induced current density used a spacing of [0.05, 0.05, 0.1] for the hypothetical {Bi6} prisms and [0.05, 0.05, 0.2] for the Ru-, Ir-, and Mo-based compounds. The magnetic field direction was chosen to be perpendicular to the molecular plane and the respective triangles. Current strengths were computed with an integration plane passing through a bond or an atom. Here, a spacing of [0.05, 0.005, 0.005] for the grid points ensured convergence. This was validated by a finer spacing of [0.01, 0.001, 0.001]. Note that we applied symmetry constraints (D_{3h}) for the current studies of the hypothetical Bi_6^{3-} and Bi_6^{4-} clusters.

6.2. Canonical Frontier Molecular Orbitals

The canonical frontier molecular orbitals (MOs) of $Bi_6^{2-}$and Bi_6^{4-} are shown in **Supplementary Figure 10**. The highest occupied molecular orbital (HOMO) of Bi_6^{2-} and the HOMO-1 of Bi_6^{4-} (D_{3h}) show a φ-type cluster orbital, which is similar to an atomic f_{z3} orbital.

According to **Supplementary Figure 11**, the HOMO-1 of **1**⁻ consists of the HOMO of Bi_6^{2-} and minor contributions from the {RuCp} fragments. The HOMO-2 contains contributions from the lowest unoccupied MO (LUMO) of Bi_6^{2-} and the HOMO of Bi_6^{4-}. The complete MO scheme is shown in the main text.

In contrast, the orbitals of **2**⁻ in **Supplementary Figure 12** differ substantially. The HOMO-26 and HOMO-10 consists of a δ-type cluster orbital at Bi_6, which is similar to an atomic d_{z2} orbital, and large contributions of the {(cod)Ir} fragment orbitals. The HOMO-5 and HOMO-6 are nearly degenerate orbitals and the contribution of the Bi_6 moiety is similar to the antibonding MOs of two ecliptic cyclopropenyl anions. The HOMO-6 and HOMO-5 of **2**⁻ again feature a considerable contribution of the {(cod)Ir} fragment. The HOMO-1 and HOMO-2 contain very distorted φ-type contributions of the Bi_6 core.

This shows that the electronic structure of **1**⁻ is characterized by very regular φ-type contributions of the Bi_6 group, whereas the respective contributions of **2**⁻ are highly distorted, in line with the molecular structure. Moreover, the Wiberg bond index[30] (WBI) of the Bi–Bi bonds in **2**⁻ is considerably smaller by about 7%.

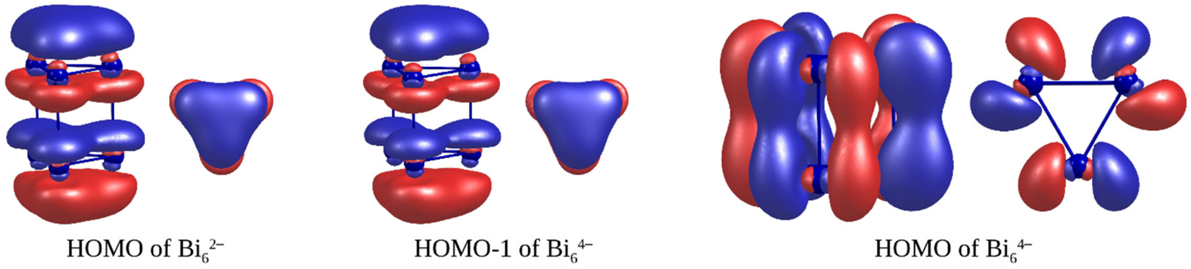

Supplementary Figure 10: Representative molecular orbitals of Bi_6^{2-} and Bi_6^{4-} with side- and top-view. An isovalue of 0.027 was used. Note that we applied the symmetry constraints of the D_{3h} point group for Bi_6^{4-}.

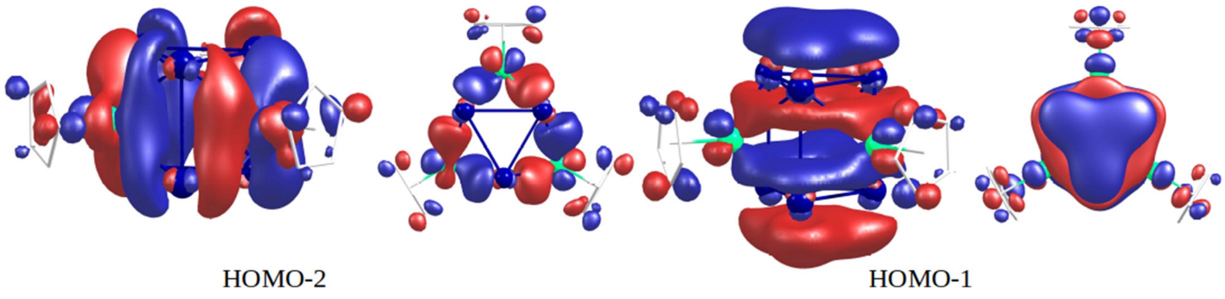

Supplementary Figure 11: Representative molecular orbitals of $[1^{calc}]^-$ with side- and top-view. An isovalue of 0.027 was used.

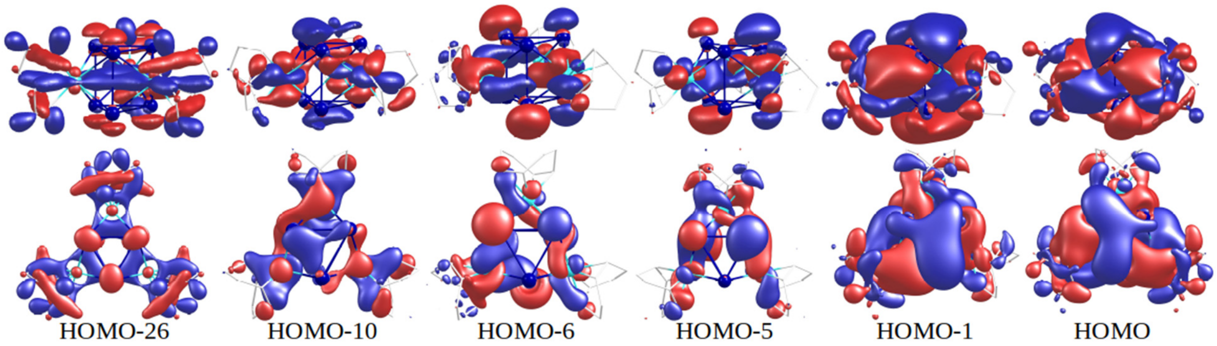

Supplementary Figure 12: Representative molecular orbitals of [2^{calc}]$^{-}$ with side- and top-view. An isovalue of 0.027 was used for HOMO-26, HOMO-10, HOMO-6, and HOMO-5. An isovalue of 0.015 was used for HOMO-1 and HOMO.

6.3. Localized Molecular Orbitals of Bi_6^{2-}

For a deeper understanding of the {Bi_6} cluster and the electronic structure of **1**⁻, we carried out an orbital localization procedure according to the Boys method[13] for the parent Bi_6^{2-} prism. The localized molecular orbitals (LMOs) are shown in **Supplementary Figure 13**. The shape of LMO 1 is similar to the canonical HOMO. According to the coefficients for the linear combinations, the HOMO also dominates this LMO with a contribution of 70%. The LMOs 2-4 describe the 2-center-2-electron (2c2e) Bi-Bi bonds between the triangles, whereas the LMOs 5-7 and 8-10 correspond to 2c2e bonds within the two triangles. Starting with LMO 10, the lone pairs at the Bi atoms are displayed. This shows that all canonical MOs except for the HOMO can be localized to describe 2c2e bonds or lone pairs.

Based on the LMOs, the bond lengths and Wiberg bond indices can be rationalized. LMO 1 strengthens the Bi-Bi bonds in the triangles and thus shortens the bond lengths. The nodal plane between the triangles weakens the bonds between the triangles. Therefore, the WBI in the triangles is increased compared to the neutral Bi_6 (1.14 for Bi_6^{2-} vs. 0.99 for Bi_6) and reveals a multi-bond character, while the WBI between the triangles is decreased (0.90 for Bi_6^{2-} vs. 0.99 for Bi_6).

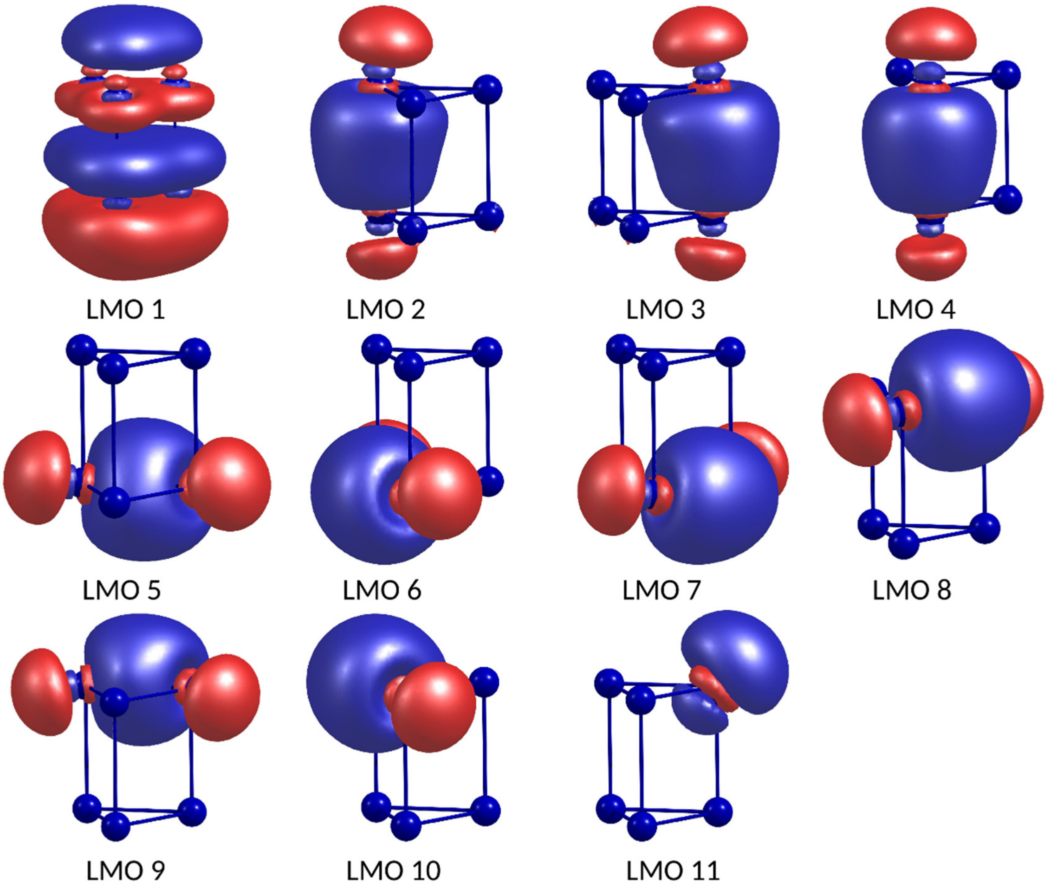

Supplementary Figure 13: Representative localized molecular orbitals of Bi_6^{2-}. The LMOs are drawn with an isovalue of 0.01 (LMO 1) or 0.027 (LMO 2 – LMO 11). The LMOs are sorted according to the energy expectation values.

6.4. Current Strengths and NICS of Bi_6^{q-} Clusters and the Ru/Ir-Based Compounds

The ring current strengths of the Bi_6^q prisms (q = 0 to 4–) and the Ru-based cluster **1**$^-$ as well as the Ir-based cluster **2**$^-$ are listed in **Supplementary Table 3** and **Supplementary Table 4** together with the respective NICS values. The integration planes are shown in **Supplementary Figures 14–18.** To compare with, we also added the π-aromatic benzene and the σ-aromatic Cu_4Li_2 with 6 delocalized electrons each as well as the parent all-metal aromatic Al_4^{2-} to these tables. The ring current was already studied with similar methods for some reference molecules, see, for instance, Ref. 31. However, we repeat the calculations with our computational settings for maximum consistency. Furthermore, we note in passing that the ring current of Cu_4Li_2 is caused by the energetically degenerate e_u MOs, while the NICS value is mainly caused by the accumulation of electron density inside the {Cu_4} ring due to the shape of the energetically lower a_{1g} orbital.[32] To study the impact of the Li^+ cation, we also considered simplified derivatives Cu_4^{2-} and $(Cu_4Li)^-$. These are also σ-aromatic based on the magnetic criterion.

For TPSS and cTPSSh, the Ru-based cluster **1**$^-$ sustains a strong net diatropic ring current, and the Ir-based cluster **2**$^-$ features a similar net diatropic ring current. The NICS values are in line with these ring currents. Including the core electrons with X2C tends to increase the ring currents. We also performed calculations with hybrid density functional approximations. Therefore, we show the results with the TPSSh[5,33] as well as Becke's half and half (BH&HLYP)[34–36] functionals in **Supplementary Table 5**. TPSSh uses 10% of exact exchange, whereas BH&HLYP uses 50% of exact exchange. Furthermore, the PBE[37] and PBE0[37.38] functionals as well as the range-separated functionals LC-ωPBE[39] and ωB97X-D[40] were employed with results shown in **Supplementray Table 6** and **Supplementary Table 7**, respectively. This does not substantially change the results for **1**$^-$ and **2**$^-$. The changes of the results between the different functionals can be attributed to the perturbed density contribution and the solution of the CPKS equations, as the unperturbed density contributions only show minor changes for the NICS values and the shielding constants of the Bi atoms itself. This is in line with no substantial changes in the shape of the orbitals. Moreover, increasing the grid size to 4a[8–10] showed that the ring currents are converged with respect to the number of grid points. To sum up, we computed a net diatropic ring current for both **1**$^-$ and **2**$^-$ with the GIMIC and NICS ansätze. Still, **2**$^-$ is not fully aromatic based on the structural criterion due to the considerably different bond lengths.

The current flow can be further analyzed with NICS centers at the capped {Bi_4} face and the {Ru_2Bi_2} face (TPSS/dhf-TZVP), this yields values of –23.3 ppm and –29.9 ppm for **1**$^-$. These are clearly smaller NICS values than that for the Bi_3 face with –44.8 ppm. The relative deviations towards the NICS value of the {Bi_3} face are 48% and 33%. Accordingly, a spherical ring current or σ-type contribution overlaps with the φ-contribution (see also the illustration of the magnetically induced current density in Sec. 6.10). For **2**$^-$, the respective mean NICS values at the capped {Bi_4} face and the {Ir_2Bi_2} face are –36.6 ppm and –33.6 ppm. These are much closer to the NICS value at the {Bi_3} triangle of –47.4 ppm. Here, the relative deviation amounts to 23% and 29%. As evident by the current density plot in Sec. 6.11, there is also a σ-type contribution and φ-type contribution observed for **2**$^-$.

The maximum diatropic current strength of the hypothetical Bi_6^q prisms is found for the dianion, while a small ring current is observed for the tetraanion (singlet configuration) with all density functionals approximations. So, the trend of the current strengths is the same for all functionals, as the anion and the trianion represent intermediate cases. Again, the different results of Bi_6^{4-} between the functionals are caused by the response of the electron density, while the unperturbed density contributions to the shielding constants and NICS are very similar. The tetraanion is also a critical case for NICS, as the shielding of the ghost atom is not sufficient to properly describe the trend of the respective ring current of Bi_6^{4-}. The ring current of the tetraanion is smaller than the ring current of the neutral Bi_6 and Bi_6^{-}, however, the NICS do not agree with this trend.

Notably, the neutral prism also sustains a ring current similar to the hypothetical neutral $ThBi_{12}$ cluster, which is σ-aromatic.[41] According to the NICS values calculated at the center of mass of the entire cluster, the ring current flow of the neutral Bi_6 prism differs from that of the dianion. A positive NICS value is found for the neutral Bi_6, whereas a negative NICS value is computed for the dianion. The core electrons and an explicit treatment of scalar relativistic effects with the DLU-X2C Hamiltonian tend to increase the absolute values of the ring currents.

Placing a ghost atom for NICS at the $\{Bi_4\}$ face for the Bi_6^q prisms leads to NICS values of +10.5 ppm (q = 0), –32.6 ppm (q = 2), and –28.0 ppm (q = 4) at the TPSS/dhf-TZVP level. This indicates a degree of spherical aromaticity for Bi_6^{2-} and Bi_6^{4-}. Therefore, both σ-type and φ-type contributions are present. The simultaneous occurrence of an σ-type and a higher-order ring current is a typical behavior of all-metal aromatic systems.[41-45]

Supplementary Table 3: Current strengths in nA/T and NICS values in ppm of various compounds with the TPSS functional. Ghost atoms for NICS are placed at the center of mass of the entire cluster and the Bi_3 face (parenthesis). DLU-X2C calculations could not be converged regarding the number of grid points for **1**⁻ and **2**⁻. The D_{3h}-optimized structures were used for Bi_6^{3-} and Bi_6^{4-}.The integration planes are shown in **Supplementary Figures 14–18.**

Compound	ECP/dhf-TZVP/TPSS		DLU-X2C/x2c-TZVPall-s/TPSS	
	Ring Current	NICS	Ring Current	NICS
Benzene	+11.8	–8.1	+11.8	–8.0
Cu_4Li_2	+18.2	–16.4	+18.5	–18.3
Cu_4^{2-}	+13.9	–22.2	+18.4	–21.9
Cu_4Li^{-}	+16.6	–19.7	+19.4	–21.1
Al_4^{2-}	+27.5	–32.3	+27.6	–32.2
**1**⁻	+25.6 (+12.8)	–21.3 (–44.8)	–	–22.2 (–45.6)
**2**⁻	+27.6 (+13.8)	–28.5 (–47.4)	–	–29.6 (–48.1)
Bi_6	+26.5 (+13.3)	+15.0 (–14.2)	+33.8 (+16.9)	+14.8 (–14.5)
Bi_6^{1-}	+30.2 (+15.1)	–6.1 (–32.4)	+37.2 (+18.6)	–6.2 (–32.7)
Bi_6^{2-}	+31.1 (+15.5)	–33.4 (–53.6)	+42.6 (+21.3)	–36.1 (–55.3)
Bi_6^{3-} (D_{3h})	+25.0 (+12.5)	–25.8 (–46.8)	+33.7 (+16.9)	–26.1 (–46.6)
Bi_6^{4-} (D_{3h})	+16.2 (+8.1)	–30.8 (–47.1)	+27.5 (+13.7)	–31.6 (–47.3)

Supplementary Table 4: Current strengths in nA/T and NICS values in ppm of various compounds with the cTPSS functional. See **Supplementary Table 3** for details.

Compound	ECP/dhf-TZVP /cTPSS		DLU-X2C/x2c-TZVPall-s/cTPSS	
	Ring Current	NICS	Ring Current	NICS
Benzene	+12.0	–8.4	+12.0	–8.4
Cu_4Li_2	+18.3	–16.6	+18.5	–18.5
Cu_4^{2-}	+14.0	–22.3	+18.5	–22.0
Cu_4Li^-	+16.8	–19.8	+19.5	–21.3
Al_4^{2-}	+27.5	–32.1	+27.6	–32.1
**1**$^-$	+25.8 (+12.9)	–21.8 (–45.3)	–	–22.7 (–46.0)
**2**$^-$	+27.6 (+13.9)	–28.9 (–47.7)	–	–30.0 (–48.4)
Bi_6	+26.8 (+13.4)	+15.1 (–14.3)	+33.9 (+17.0)	+14.9 (–14.6)
Bi_6^-	+28.4 (+14.2)	–2.6 (–30.1)	+38.6 (+19.3)	–5.4 (–32.0)
Bi_6^{2-}	+31.3 (+15.7)	–33.7 (–53.9)	+42.7 (+21.4)	–36.3 (–55.5)
Bi_6^{3-} (D_{3h})	+25.1 (+12.6)	–26.0 (–47.0)	+33.7 (+16.9)	–26.4 (–46.7)
Bi_6^{4-} (D_{3h})	+16.1 (+8.1)	–31.0 (–47.3)	+27.1 (+13.6)	–31.9 (–47.4)

Supplementary Table 5: Current strengths in nA/T and NICS values in ppm of various compounds with the TPSSh and BH&HLYP functionals (ECP/dhf-TZVP). See **Supplementary Table 3** for details.

Compound	TPSSh		BH&HLYP	
	Ring Current	NICS	Ring Current	NICS
Benzene	+11.9	–8.2	+12.4	–8.8
Cu_4Li_2	+18.9	–14.7	+20.5	–9.0
Cu_4^{2-}	+14.4	–20.4	+15.5	–15.2
Cu_4Li^-	+17.2	–17.7	+18.5	–12.0
Al_4^{2-}	+27.6	–33.2	+28.1	–38.4
**1**$^-$	+26.8 (+13.4)	–22.7 (–56.7)	+30.3 (+15.2)	–28.9 (–52.7)
**2**$^-$	+28.2 (+14.2)	–29.3 (–48.6)	+30.4 (+15.3)	–31.8 (–51.8)
Bi_6	+27.7 (+13.8)	+13.8 (–16.1)	+28.9 (+14.4)	+13.1 (–17.7)
Bi_6^{1-}	+31.2 (+15.6)	–5.2 (–32.4)	+32.3 (+16.1)	–7.3 (–34.6)
Bi_6^{2-}	+34.6 (+17.3)	–36.5 (–56.0)	+35.7 (+17.8)	–37.7 (–58.0)
Bi_6^{3-} (D_{3h})	+25.6 (+12.8)	–26.6 (–48.0)	+27.2 (+13.8)	– 28.0 (–50.7)
Bi_6^{4-} (D_{3h})	+16.7 (+8.4)	–31.8 (–48.3)	+18.1 (+9.1)	–33.7 (–51.5)

Supplementary Table 6: Current strengths in nA/T and NICS values in ppm of various compounds with the PBE and PBE0 functionals (ECP/dhf-TZVP). See **Supplementary Table 3** for details.

Compound	PBE		PBE0	
	Ring Current	NICS	Ring Current	NICS
Benzene	+11.8	–7.7	+12.2	–8.2
Cu_4Li_2	+18.1	–16.7	+19.7	–12.4
Cu_4^{2-}	+14.1	–22.0	+15.0	–18.0
Cu_4Li^-	+16.7	–19.5	+17.9	–15.1
Al_4^{2-}	+27.6	–31.8	+27.8	–34.5
**1**$^-$	+25.2 (12.6)	–21.3 (–44.2)	+28.1 (+14.1)	–24.9 (–44.1)
**2**$^-$	+27.0 (+13.6)	–28.3 (–46.8)	+29.1 (+14.7)	–30.3 (–49.9)
Bi_6	+27.3 (+13.7)	+14.8 (–14.6)	+28.3 (+14.1)	+13.5 (–16.8)
Bi_6^-	+30.8 (+15.4)	–4.8 (–31.1)	+31.8 (+15.9)	–6.8 (–33.7)
Bi_6^{2-}	+34.2 (+17.1)	–35.6 (–55.0)	+35.2 (+17.6)	–37.5 (–57.4)
Bi_6^{3-} (D_{3h})	+24.5 (+12.3)	–25.0 (–46.6)	+26.1 (+13.0)	–27.4 (–49.5)
Bi_6^{4-} (D_{3h})	+15.2 (+7.6)	–30.1 (–46.6)	+16.7 (+8.3)	–32.6 (–49.7)

Supplementary Table 7: Current strengths in nA/T and NICS values in ppm of various compounds with the LC-ωPBE and ωB97X-D functionals (ECP/dhf-TZVP). See **Supplementary Table 3** for details.

Compound	LC-ωPBE		ωB97X-D	
	Ring Current	NICS	Ring Current	NICS
Benzene	+12.3	–8.1	+12.2	–8.1
Cu_4Li_2	+20.0	–11.9	+20.1	–11.9
Cu_4^{2-}	+15.4	–17.2	+15.2	–17.0
Cu_4Li^-	+18.3	–14.5	+18.2	–14.4
Al_4^{2-}	+28.0	–43.6	+28.1	–39.4
**1**$^-$	+30.5 (+15.3)	–27.4 (–53.2)	+29.1 (+14.6)	–25.8 (–50.9)
**2**$^-$	+30.9 (+15.6)	–31.1 (–52.9)	+29.8 (+15.0)	–30.1 (–50.9)
Bi_6	+29.6 (+14.8)	+9.4 (–21.4)	+29.2 (+14.6)	+12.4 (–18.6)
Bi_6^{1-}	+33.2 (+16.6)	–11.6 (–38.8)	+32.8 (+16.4)	–6.7 (–34.7)
Bi_6^{2-}	+36.7 (+18.4)	–40.5 (–61.5)	+35.6 (+18.1)	–38.5 (–59.5)
Bi_6^{3-} (D_{3h})	+28.8 (+14.4)	–31.7 (–55.8)	+27.7 (+13.9)	–28.4 (–52.5)
Bi_6^{4-} (D_{3h})	+19.6 (+9.8)	–37.0 (–56.8)	+18.1 (+9.0)	–32.12 (–52.9)

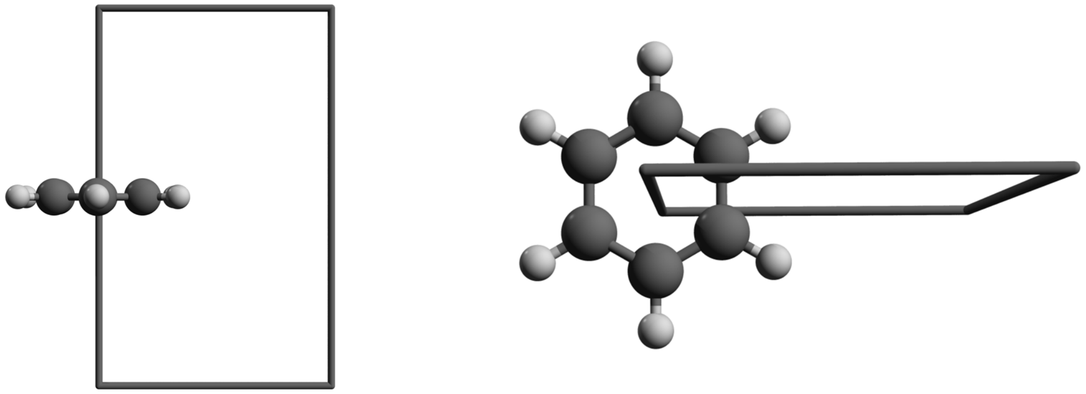

Supplementary Figure 14: Placement of the integration plane for the current strength calculations of benzene. Side-view and top-view are shown. The magnetic field is perpendicular to the molecular plane.

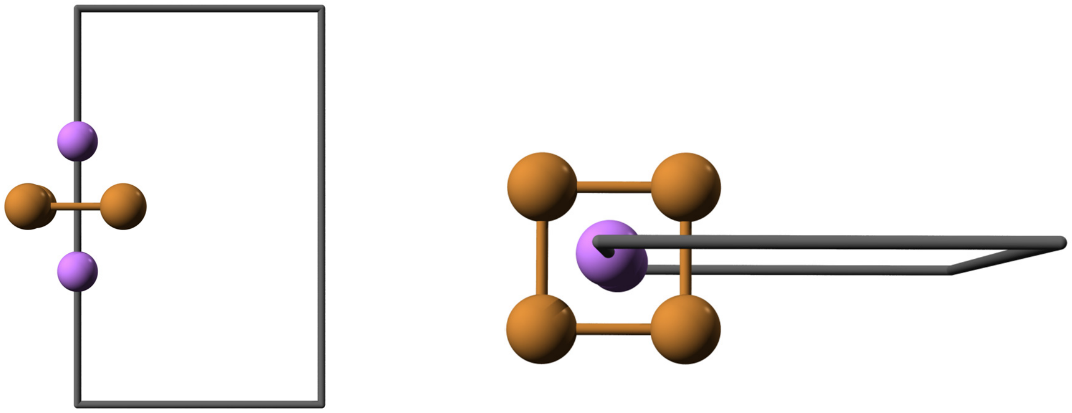

Supplementary Figure 15: Placement of the integration plane for the current strength calculations of Cu_4Li_2. Side-view and top-view are shown. The magnetic field is perpendicular to the molecular plane. The planes are placed in the same manner for Cu_4^{2-} and Cu_4Li^-.

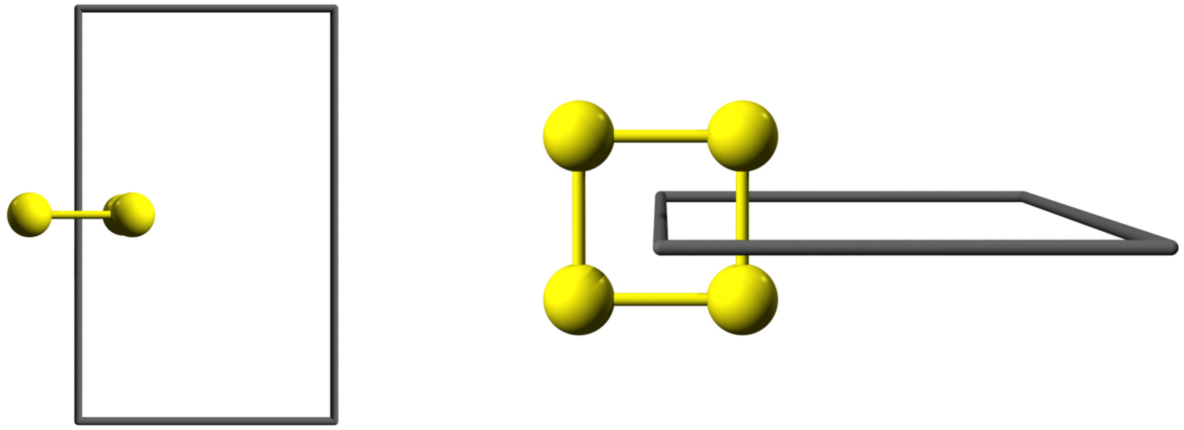

Supplementary Figure 16: Placement of the integration plane for the current strength calculations of Al_4^{2-}. Side-view and top-view are shown. The magnetic field is perpendicular to the molecular plane.

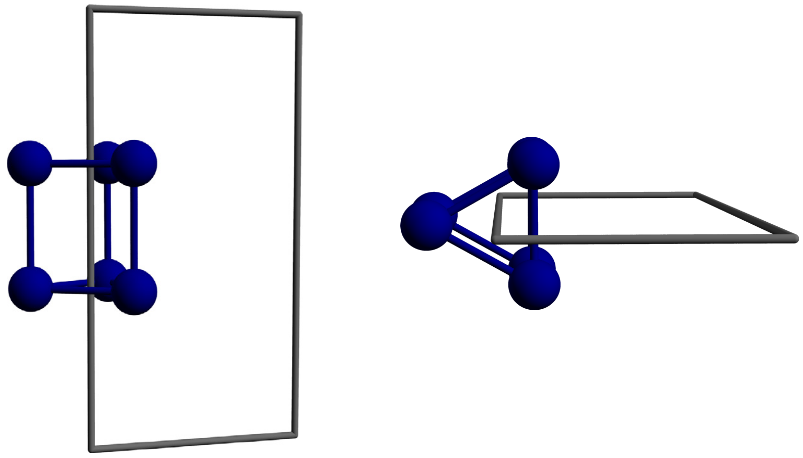

Supplementary Figure 17: Placement of the integration plane for the current strength calculations of the Bi_6 clusters. Side-view and top-view are shown. The magnetic field is perpendicular to the Bi_3 triangles. For the Ru-based and Ir-based clusters, the integration plane passes through two Bi atoms.

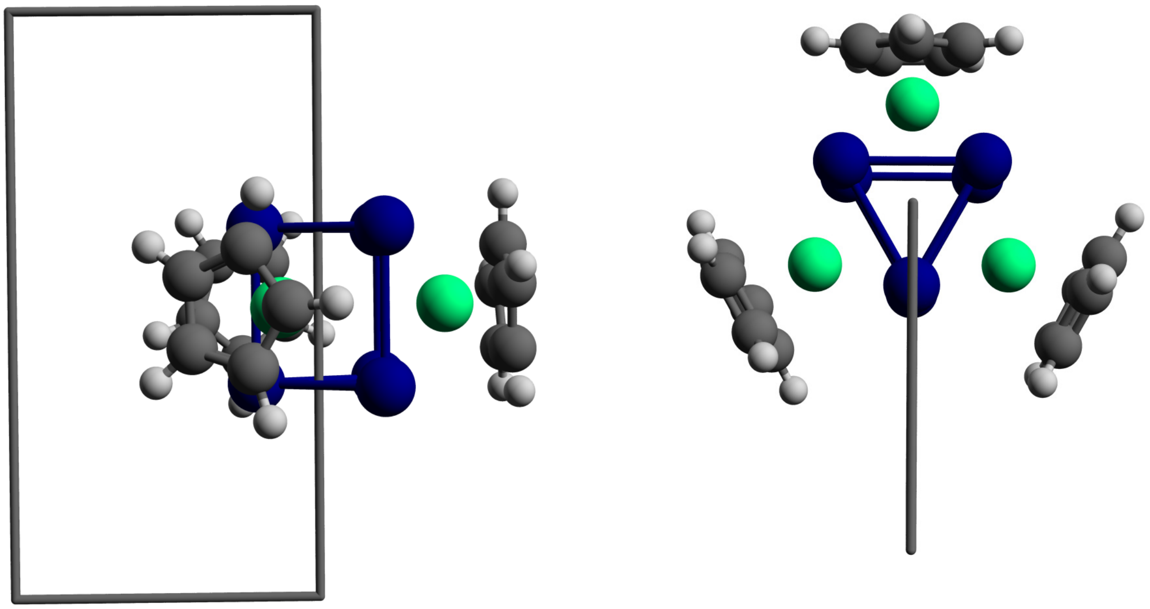

Supplementary Figure 18: Placement of the integration plane for the current strength calculations of the Ru-based cluster. Side-view and top-view are shown. The magnetic field is perpendicular to the $\{Bi_3\}$ triangles. The plane is placed similarly for the Ir-based cluster.

6.5. Current Profiles with Respect to the Height

The current profiles with respect to the height are shown in **Supplementary Figures 19–22** for benzene (**Supplementary Figure 19**), Cu_4Li_2 (**Supplementary Figure 20**), Al_4^{2-} (**Supplementary Figure 21**), and the neutral, prismatic Bi_6 as well as its dianion and tetraanion (all in **Supplementary Figure 22**). All current profiles start at the center of mass of the entire molecules. We partitioned the integration planes shown above into slices with a height 0.1 bohr and the full width.

The current profile of benzene clearly resembles the π-shape of the orbitals. The minimum of the current strength is in the molecular plane due to the nodal structure of the π-orbitals. In contrast, Cu_4Li_2 shows the maximum current strength in the molecular plane and a sharp peak caused by the contribution of the Li cation. The result herein is in line with high-level coupled-cluster studies.[31] Al_4^{2-} shows contributions of both σ- and π-type orbitals. The width of the peak at the center is notably increased due to the latter, while the maximum of the current strength is still in the molecular plane. This shows that the σ-contribution is larger than the π-contribution. According to previous ring current studies,[42-44] Al_4^{2-} is also both σ-aromatic and π-aromatic. The π-contribution amounts to 15–39%.[41-44] This synergy of σ-type and π-type contributions is a characteristic feature of many aromatic metal clusters.[45]

In **Supplementary Figure 22**, Bi_6 shows the maximum current strength in the Bi_3 plane. The curve corresponds to that of known σ-aromatic systems such as $\{Cu_4Li_2\}$.[32] The dianion features a wider curve but retains the overall structure of the current profile of Bi_6. The wider curve is in line with the shape of the HOMO. The current strength still features a minimum at the center of mass due to the nodal plane of this orbital. Therefore, Bi_6^{2-} is both σ-aromatic and φ-aromatic according to the magnetic criterion. The population of the φ-type cluster orbital in Bi_6^{2-} increases the ring current strength by about +5 nA/T. For the tetraanion in D_{3h} symmetry, a substantial decrease of the ring current inside the $\{Bi_3\}$ plane is observed in line with the population of the HOMO in **Supplementatry Figure 10**. The HOMO of Bi_6^{4-} mainly affects the ring current inside the Bi triangles according to the nodal structure.

Based on the plots of the magnetically induced current density below (cf. **Supplementary Figures 25-29**), the σ-contribution is weakened for the clusters **1**$^-$ and **2**$^-$. This can be rationalized by the population of the HOMO-2 of **1**$^-$ (see main text **Fig. 4**, MO 144a). The contribution of this MO at the Bi_6 core corresponds to the LUMO of Bi_6^{2-}.

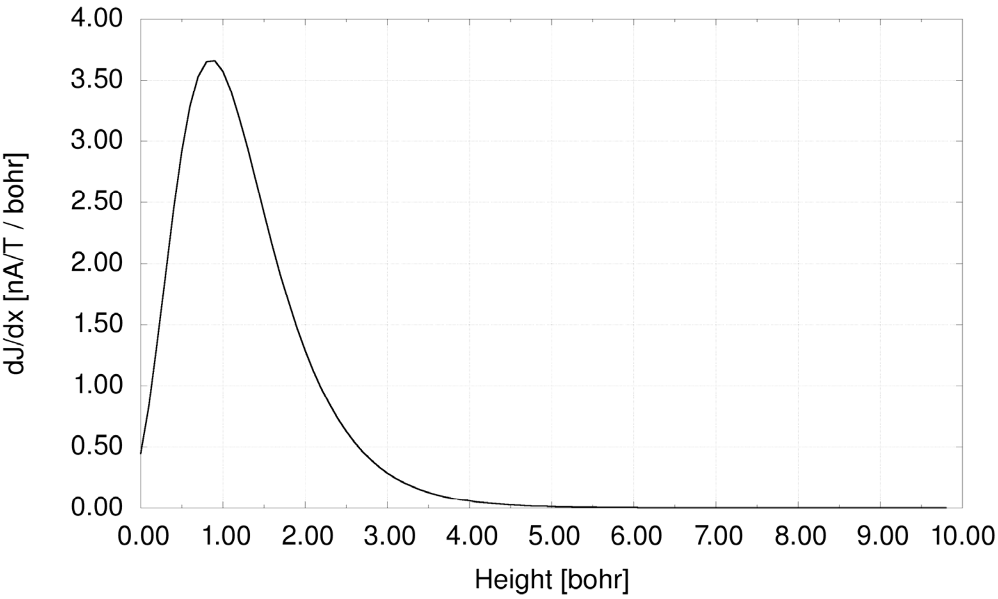

Supplementary Figure 19: Current density profile with respect to the height of the ring current strength calculated for benzene. The profiles start at the center of the mass. A positive sign indicates a diatropic contribution. According to the structure, benzene is π-aromatic.

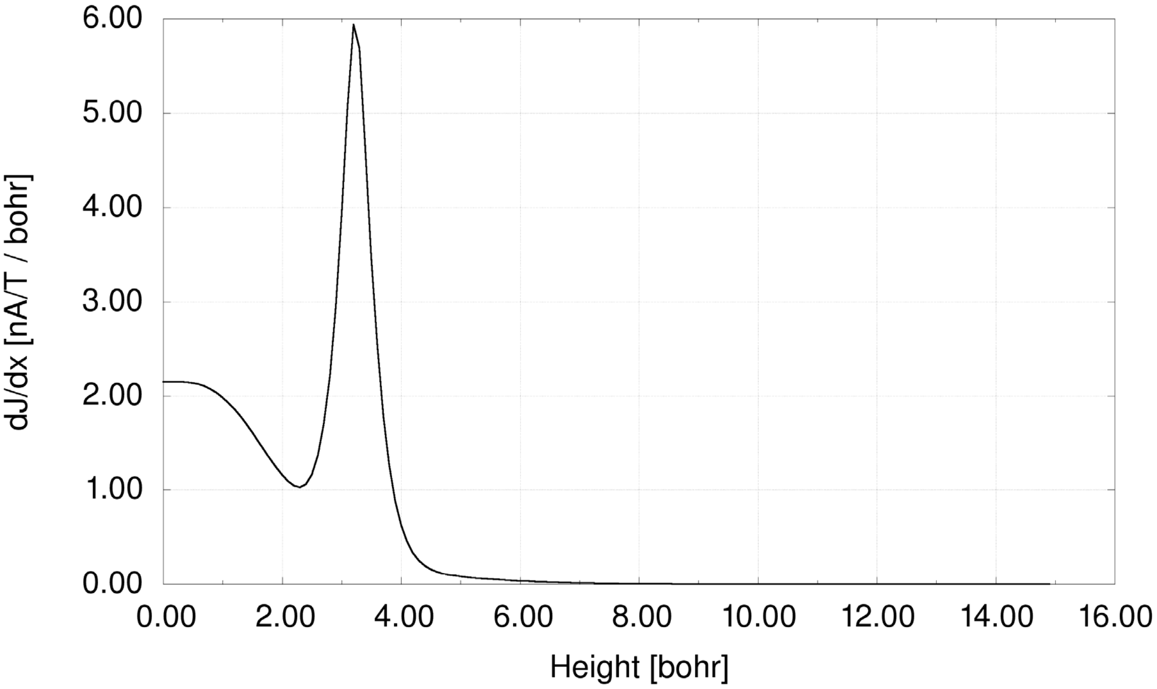

Supplementary Figure 20: Current density profile with respect to the height of the ring current strength calculated for Cu_4Li_2. The profiles start at the center of the mass. A positive sign indicates a diatropic contribution. The sharp peak between 3 and 4 bohr is due to the Li cation, those interaction with the {Cu_4} ring affects the total ring current. According to the width of the curve between 0 an 2 bohr, this compound is σ-aromatic.

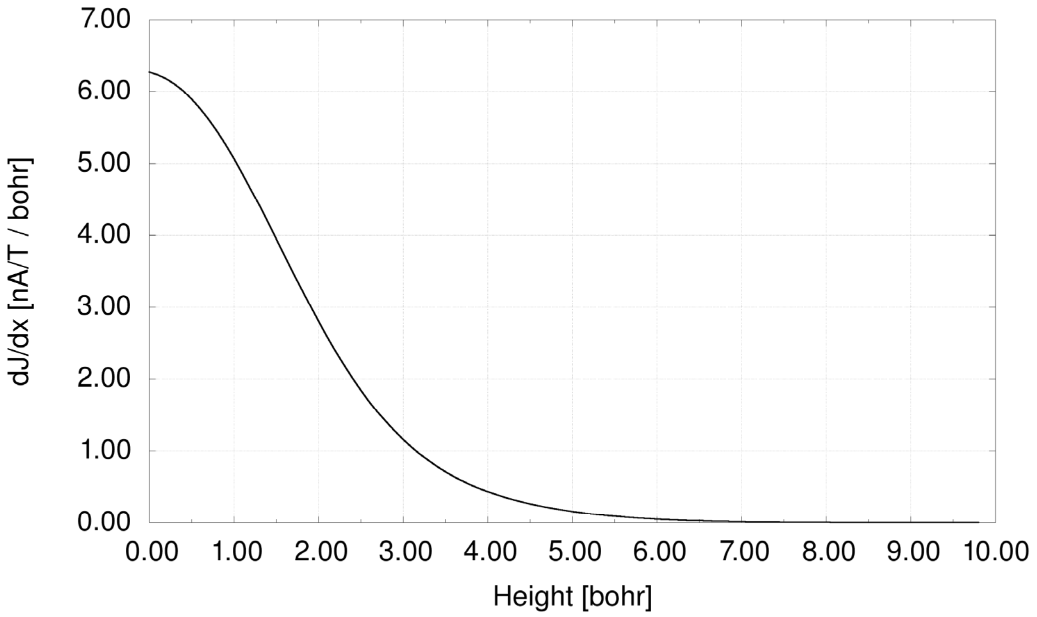

Supplementary Figure 21: Current density profile with respect to the height of the ring current strength calculated for Al_4^{2-}. The profiles start at the center of the mass. A positive sign indicates a diatropic contribution. This compound is both σ-aromatic and π-aromatic.

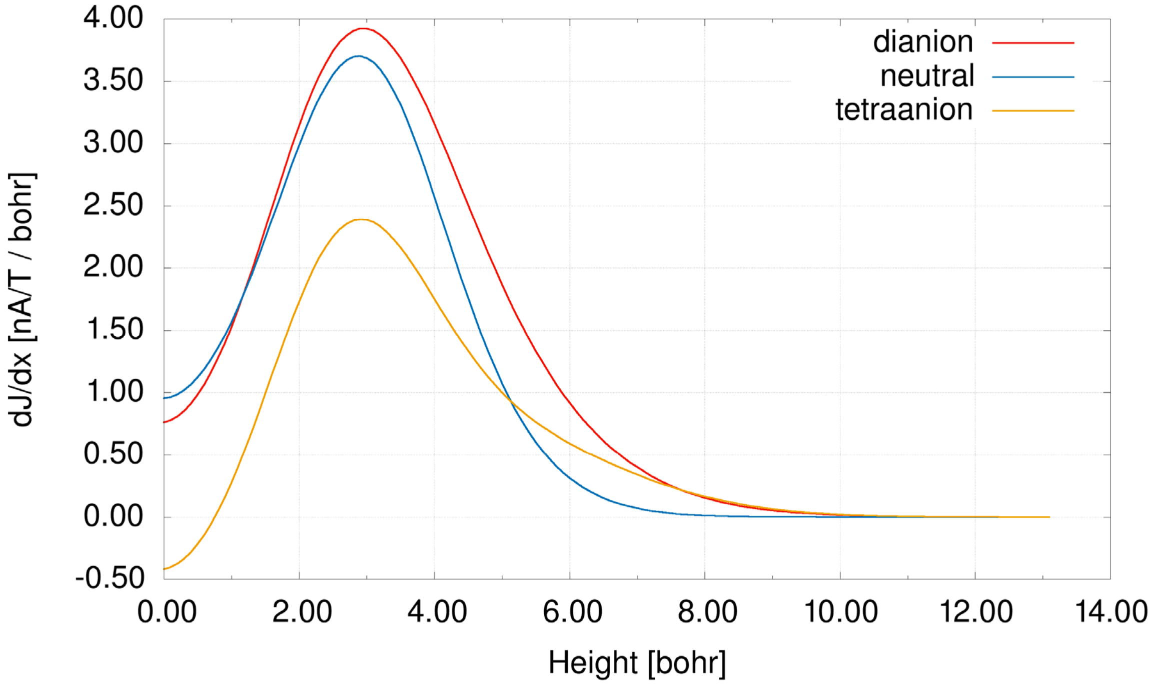

Supplementary Figure 22: Current density profile with respect to the height of the ring current strength calculated for Bi_6, Bi_6^{2-}, and Bi_6^{4-}. The profiles start at the center of the mass. Blue curve, Bi_6; this compound is only σ-aromatic. Red curve, Bi_6^{2-}; this compound is both σ-aromatic and φ-aromatic. Orange curve, Bi_6^{4-} in D_{3h} symmetry; this compound is also both σ-aromatic and φ-aromatic based on the magnetic criterion. For Bi_6, Bi_6^{2-}, and Bi_6^{4-}, the Bi–Bi connection line is intersected at about 2.9 bohr, 3.2 bohr, and 3.1 bohr. A positive sign indicates a diatropic contribution, a negative sign indicates a paratropic contribution.

6.6. Current Distance Profiles of Bi_6^{2-} and Bi_6^{4-}

The current profiles with respect to the distance and height of Bi_6^{2-} and Bi_6^{4-} (D_{3h}) calculated of with TPSS are shown in **Supplementary Figure 23** and **Supplementary Figure 24**, respectively. A strong diatropic ring current is found outside the Bi_6 prisms, whereas weak paratropic ring current is present inside the {Bi_6} prisms.

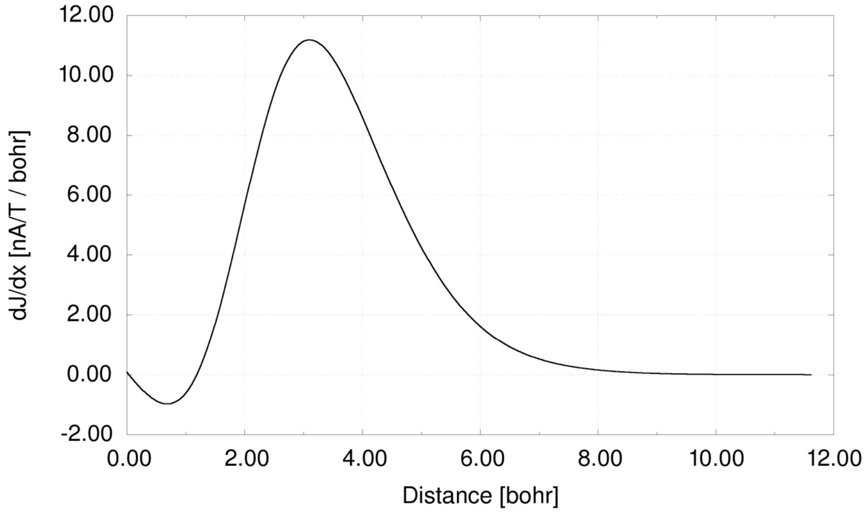

Supplementary Figure 23: Current density profile with respect to the distance of the ring current strength calculated with TPSS for Bi_6^{2-}. The profile starts at the center of the prism and the Bi–Bi connection line is intersected at 1.65 bohr. A positive sign indicates a diatropic contribution, a negative sign indicates a paratropic contribution.

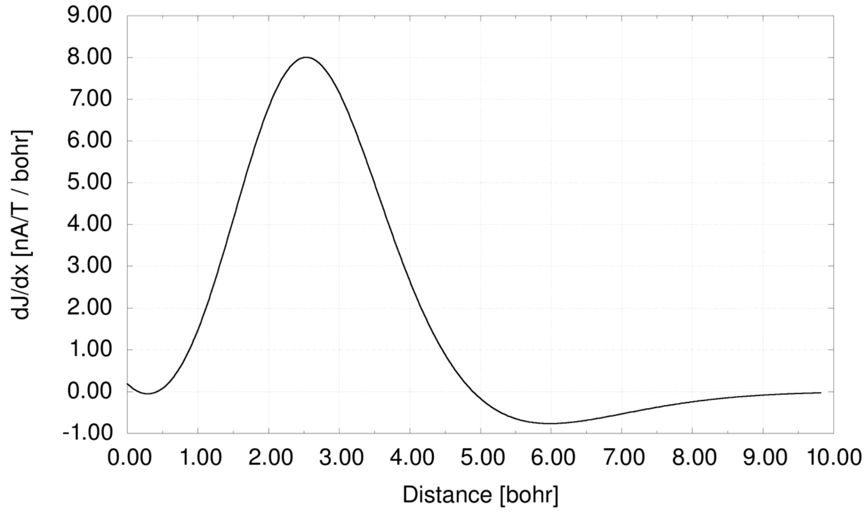

Supplementary Figure 24: Current density profile with respect to the distance of the ring current strength calculated with TPSS for Bi_6^{4-} (D_{3h}) . The profile starts at the center of the prism and the Bi–Bi connection line is intersected at 1.85 bohr. A positive sign indicates a diatropic contribution, a negative sign indicates a paratropic contribution.

6.7. Current Density Plot of Bi_6

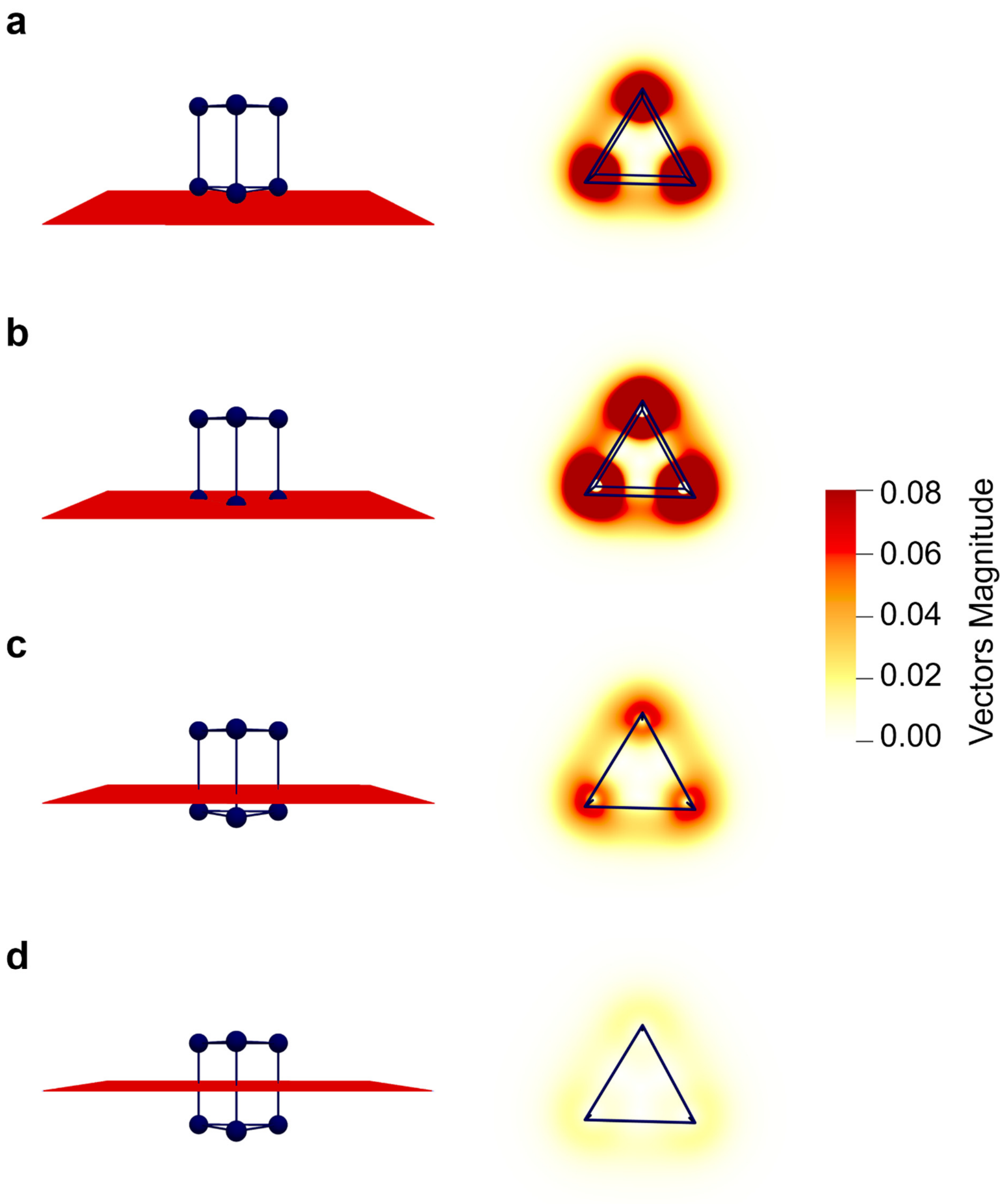

Supplementary Figure 25: Illustration of the current density calculated for the model system Bi_6. a, Vector magnitude of magnetically induced current density (in nA/T), calculated in a plane 1 bohr below the lower $\{Bi_3\}$ unit (right) and perpendicular view of the plot to illustrate its position in the prism (left). **b,** Respective results calculated in the Bi_3 plane. **c,** Respective results calculated in a plane between the center of mass of the $\{Bi_3\}$ unit and the global center of mass. **d,** Respective results calculated in a plane in the center of the $\{Bi_6\}$ prism.

6.8. Current Density Plot of Bi_6^{2-}

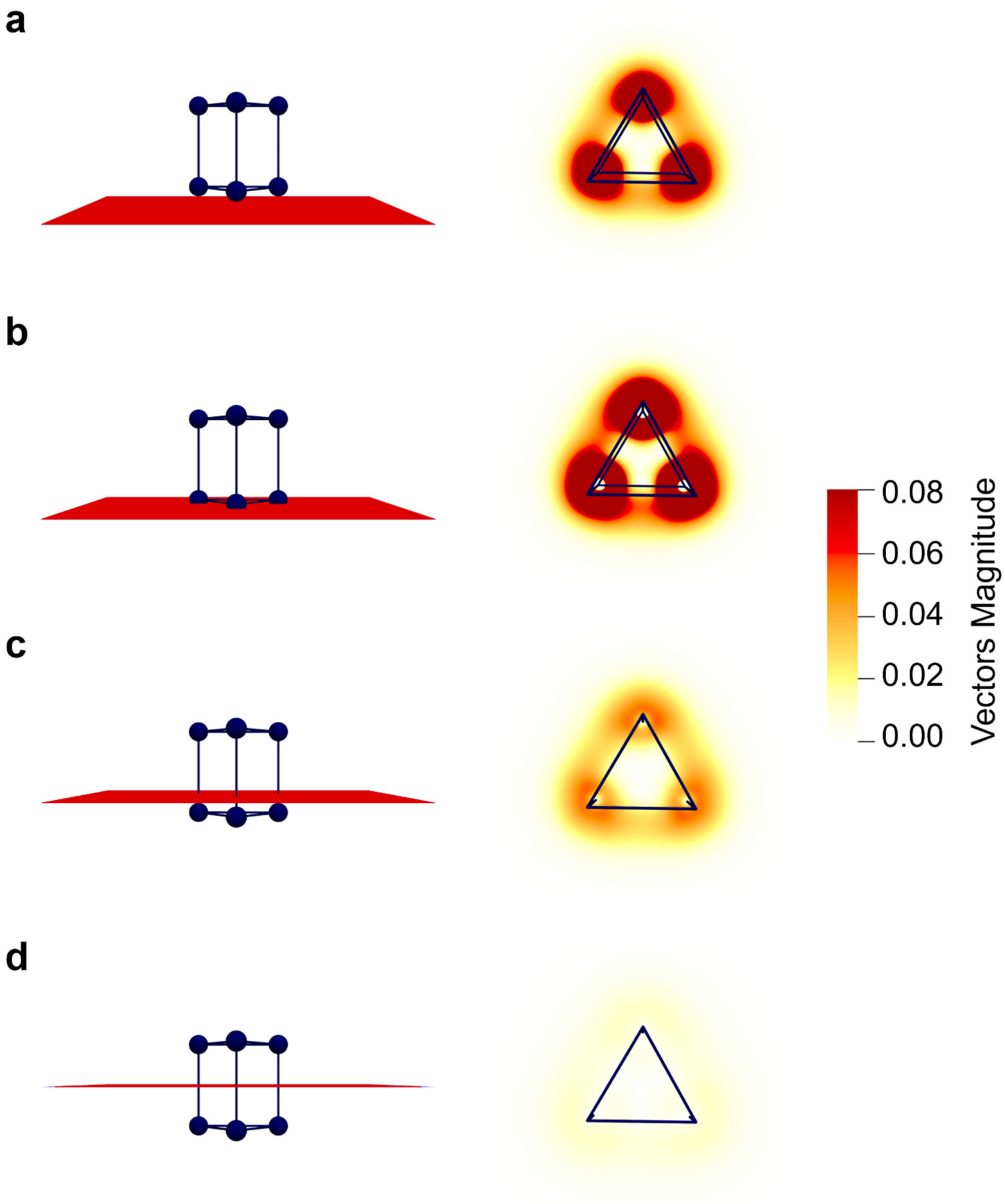

Supplementary Figure 26: Illustration of the current density calculated for the model system Bi_6^{2-}. a, Vector magnitude of magnetically induced current density (in nA/T), calculated in a plane 1 bohr below the lower {Bi_3} unit (right) and perpendicular view of the plot to illustrate its position in the prism (left). **b,** Respective results calculated in the Bi_3 plane. **c,** Respective results calculated in a plane between the center of mass of the {Bi_3} unit and the global center of mass. **d,** Respective results calculated in a plane in the center of the {Bi_6} prism.

6.9. Current Density Plot of Bi_6^{4-}

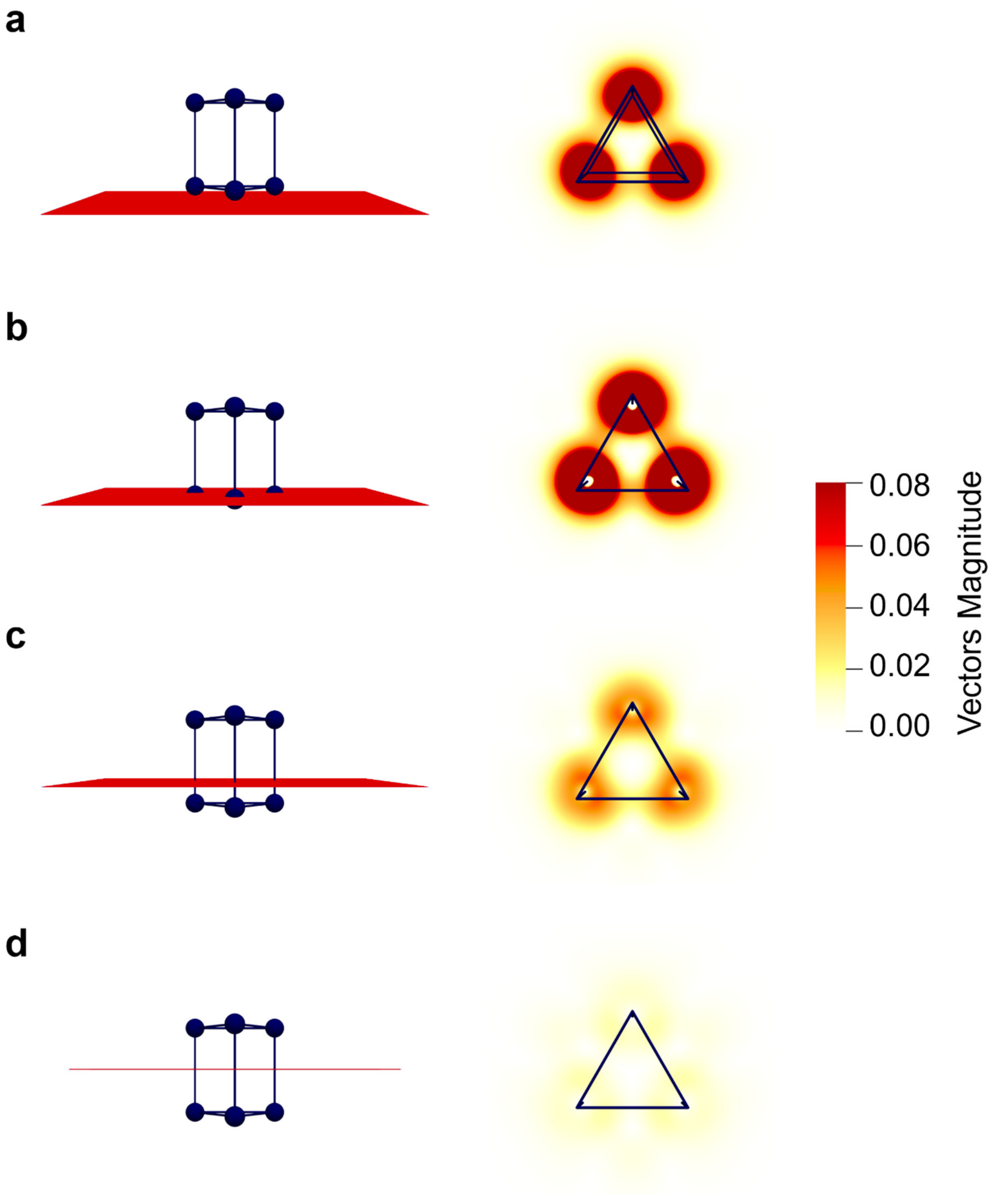

Supplementary Figure 27: Illustration of the current density calculated for the model system Bi_6^{4-}. a, Vector magnitude of magnetically induced current density (in nA/T), calculated in a plane 1 bohr below the lower {Bi_3} unit (right) and perpendicular view of the plot to illustrate its position in the prism (left). **b,** Respective results calculated in the Bi_3 plane. **c,** Respective results calculated in a plane between the center of mass of the {Bi_3} unit and the global center of mass. **d,** Respective results calculated in a plane in the center of the {Bi_6} prism.

6.10. Current Density Plot of 1^{-}

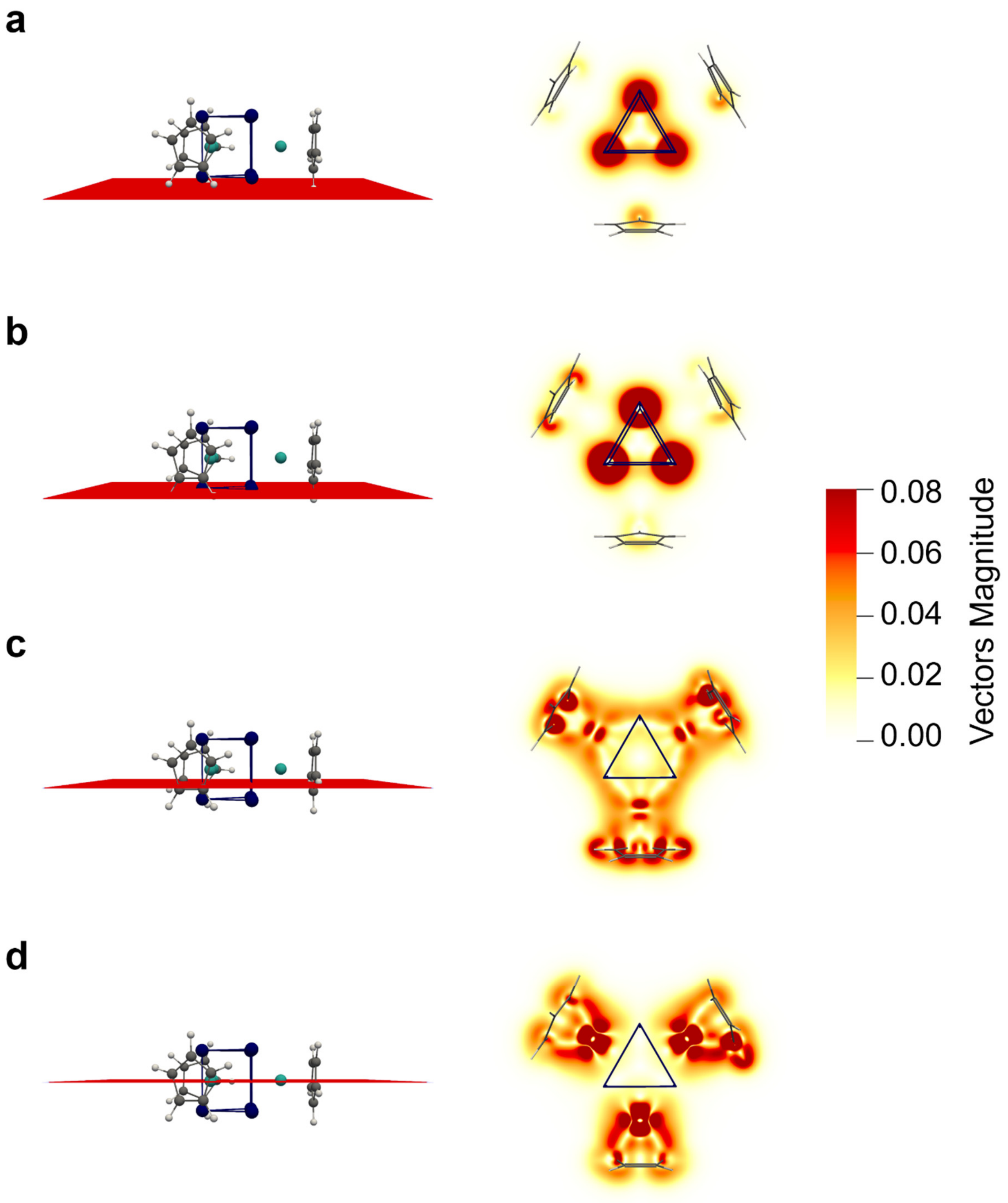

Supplementary Figure 28: Illustration of the current density calculated for 1^{-}. a, Vector magnitude of magnetically induced current density (in nA/T), calculated in a plane 1 bohr below the lower $\{Bi_3\}$ unit (right) and perpendicular view of the plot to illustrate its position in the prism (left). **b,** Respective results calculated in the Bi_3 plane. **c,** Respective results calculated in a plane between the center of mass of the $\{Bi_3\}$ unit and the global center of mass. **d,** Respective results calculated in a plane in the center of the $\{Bi_6\}$ prism.

6.11. Current Density Plot of 2^{-}

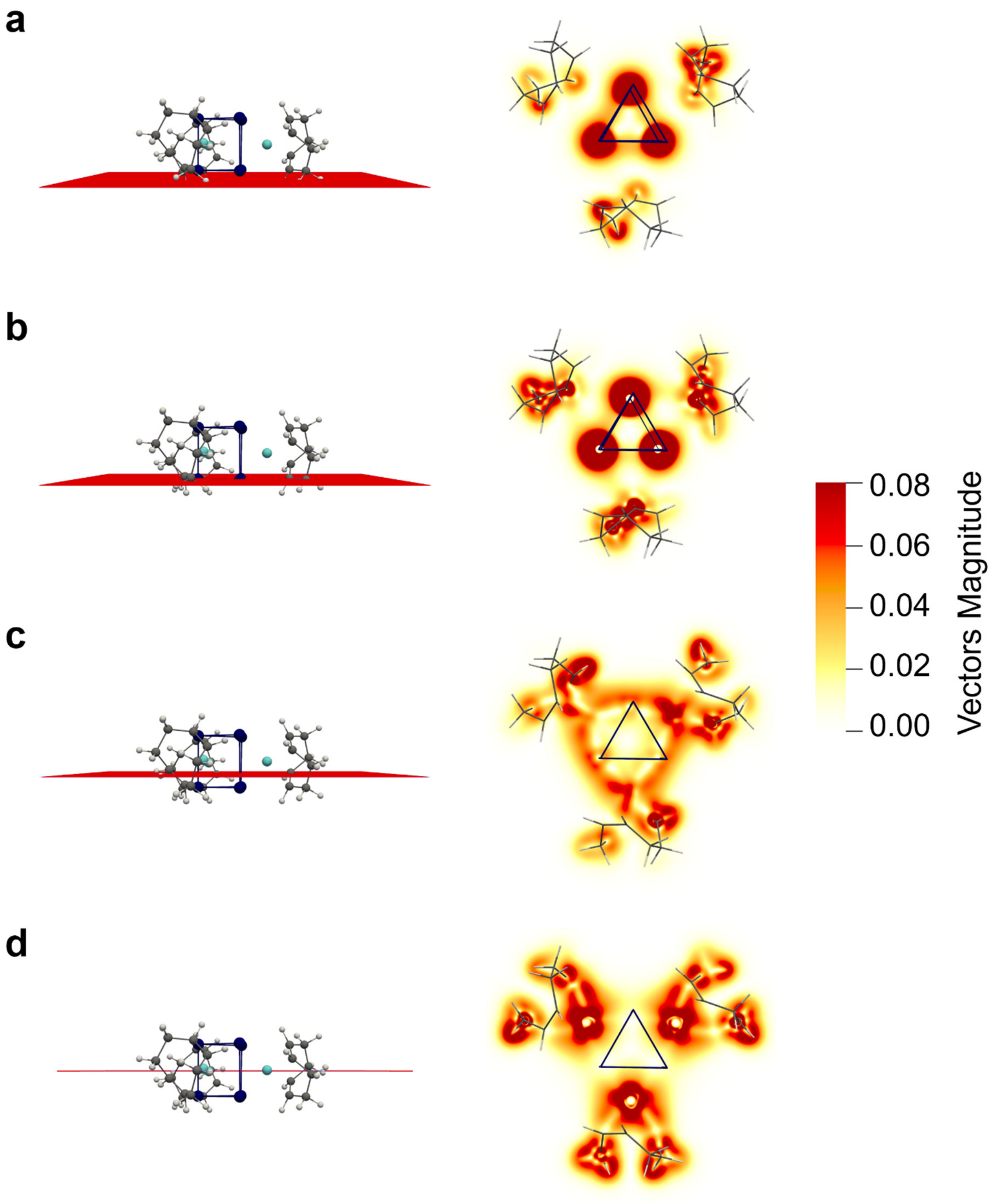

Supplementary Figure 29: Illustration of the current density calculated for 2^{-}. a, Vector magnitude of magnetically induced current density (in nA/T), calculated in a plane 1 bohr below the lower $\{Bi_3\}$ unit (right) and perpendicular view of the plot to illustrate its position in the prism (left). **b,** Respective results calculated in the Bi_3 plane. **c,** Respective results calculated in a plane between the center of mass of the $\{Bi_3\}$ unit and the global center of mass. **d,** Respective results calculated in a plane in the center of the $\{Bi_6\}$ prism.

6.12. Comparison to $[\{(CO)_3Mo\}_3Bi_6]^{4-}$

The cluster $[\{(CO)_3Mo\}_3Bi_6]^{4-}$ features a similar φ-type orbital,[46] depicted in **Supplementary Figure 30**. However, the $\{Bi_6\}$ core is strongly distorted. Still, we obtain a NICS value of –45.2 ppm at the center of mass with the TPSS/dhf-TZVP method and the experimentally determined structure. At the two $\{Bi_3\}$ triangles NICS values of –59.2 ppm and –61.8 ppm are found. The mean NICS values at the capped $\{Bi_4\}$ and the $\{Mo_2Bi_2\}$ faces are –48.3 ppm and –45.6 ppm. Therefore, the largest relative deviation of the NICS values of the quadrangles and the triangles is found for the Ru-based cluster.

The ring current of $[\{(CO)_3Mo\}_3Bi_6]^{4-}$ amounts to about +38.3 nA/T, with an integration plane as shown in **Supplementary Figure 31**. Due to the degree of structural distortion, the numerical integration is less accurate than for the other clusters. The magnetically induced current density is illustrated in **Supplementary Figure 32**. Here, a σ-contribution and a φ-contribution can be seen. As the structure is highly distorted, the ring current strength is not directly comparable to the Ru-based cluster and the regular $\{Bi_6\}$ prism. The ring current not only depends on the degree of electron delocalization and the number of delocalized electrons but also on the structure, the topology, and the chemical element.[45]

Considering the core electrons with the scalar-relativistic X2C Hamiltonian leads to NICS values of –45.8 ppm at the center of mass as well as –59.9 and –62.5 ppm for the $\{Bi_3\}$ triangles, respectively. Therefore, this cluster also meets the magnetic criterion of aromaticity and shows a (structurally distorted) φ-type orbital. The degree of distortion indicates that more negative charge is accumulated at the $\{Bi_6\}$ core than for the Ru-based cluster. A natural bond orbital (NBO) analysis[47] leads to 0.90 excess electrons in the p orbitals. Here, the Ru-based and Ir-based clusters feature 0.22 and 0.65 excess electrons, respectively. Thus, this result is in line with the geometric structure and the increasing degree of distortion of the $\{Bi_6\}$ prism. This also supports the findings of Ref. 46, where a Bi_6^{4-} core was discussed. As shown in **Supplementary Figure 10**, the φ-type orbital is also present for Bi_6^{4-}.

For completeness, we optimized the structure at the TPSS/dhf-TZVP level using COSMO with the default parameters. The structure is given in a separate file (optimized-structures.txt). Notably, this results in a more regular structure. However, the electronic structure is preserved, i.e., the NBO yields very similar results and also the φ-type orbital is still present. Furthermore, the NICS values are almost unchanged with –42.3 ppm at the center of mass and –58.1/–57.6 ppm for the triangles. Again, the very similar results are obtained with scalar-relativistic X2C. The cluster sustains a net diatropic ring current of about +37.0 nA/T.

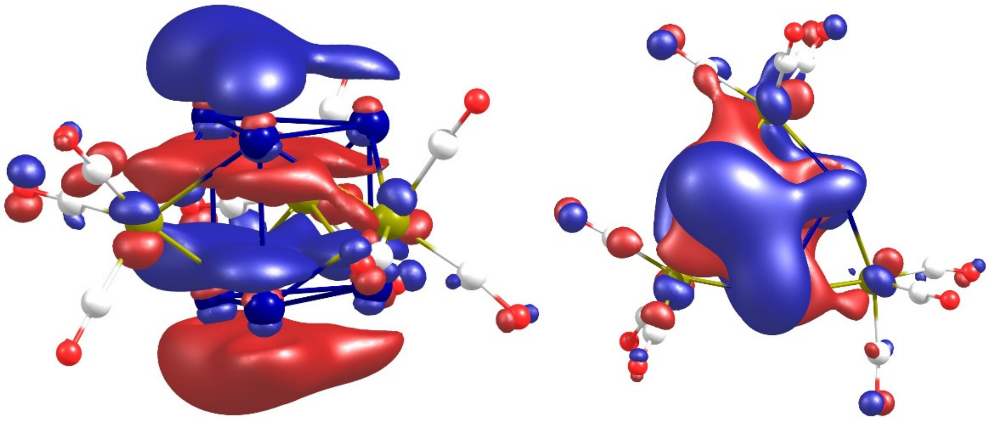

Supplementary Figure 30: HOMO of the Mo-based $[\{(CO)_3Mo\}_3Bi_6]^{4-}$ cluster. Drawn with an isovalue of 0.027.

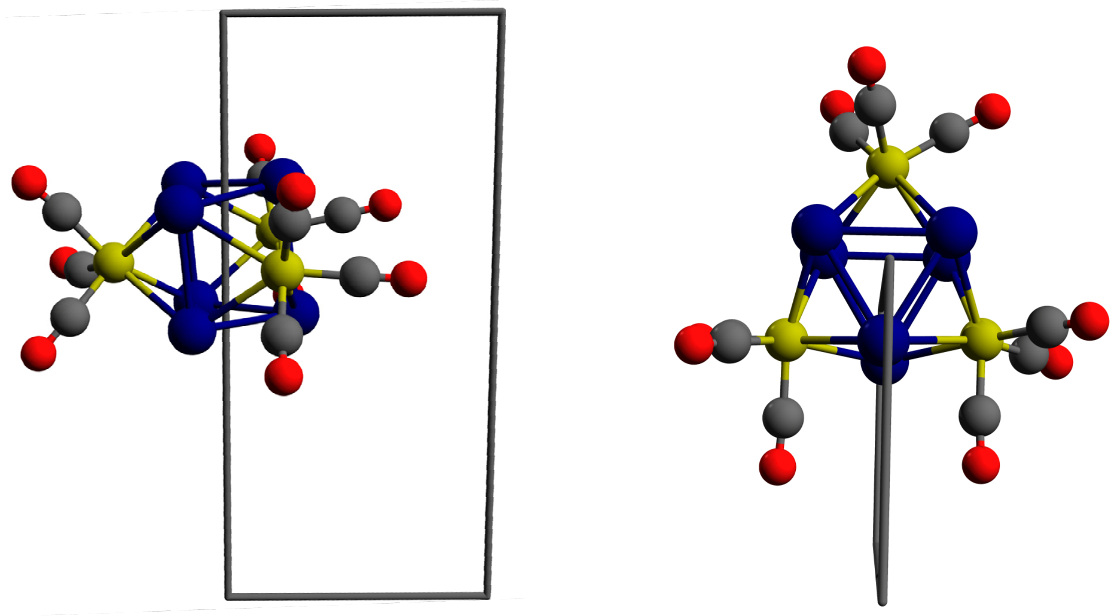

Supplementary Figure 31: Placement of the integration plane for the current strength calculations of the Mo-based cluster. Side-view and top-view are shown. The magnetic field is perpendicular to the Bi_3 triangles. Note that the structure of the Bi_6 core is highly distorted and this leads to a larger numerical integration error than for the Ru-based cluster.

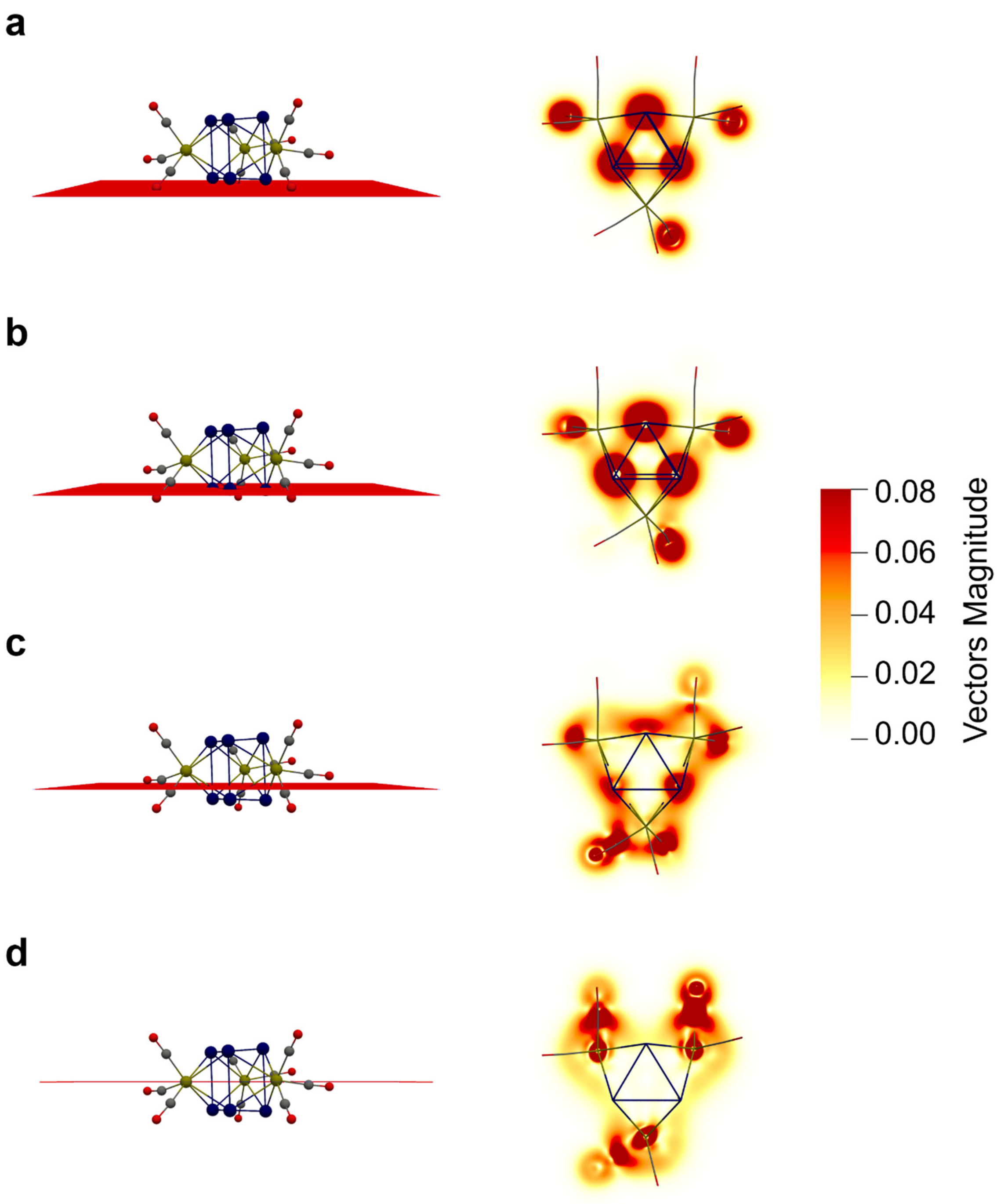

Supplementary Figure 32: Illustration of the current density calculated for $[\{(CO)_3Mo\}_3Bi_6]^{4-}$. a, Vector magnitude of magnetically induced current density (in nA/T), calculated in a plane approximately 1 bohr below the lower $\{Bi_3\}$ unit (right) and perpendicular view of the plot to illustrate its position in the prism (left). **b,** Respective results calculated in the approximate Bi_3 plane. **c,** Respective results calculated in a plane between the center of mass of the $\{Bi_3\}$ unit and the global center of mass. **d,** Respective results calculated in a plane in the center of the Bi_6 prism.

6.13. Current Strengths of Prismane

The current strengths and NICS values of prismane are listed in **Supplementary Table 8**. Here, the integration plane is placed as was done for the Bi_6 prisms, see **Supplementary Figure 17**. **Supplementary Figure 33** shows the current profile. For this current profile, the integration plane was partitioned into slices with a width of 0.02 bohr and the full height. See also Figures 8 and 9 of Ref. 45 for theoretical details on this type of current profiles.

The NICS(1), NICS(2), and NICS(–1) values with respect to the upper C_3 plane indicate that the current flows mainly above and below the complete molecule. The corresponding values are –23.4 ppm, –10.3 ppm, and –4.6 ppm. This is further confirmed by the ring current strength above the upper C_3 triangle, which amounts to +5.4 nA/T. Extending the plane up to 0.5 bohr below the upper C_3 plane yields a ring current of +7.1 nA/T. Therefore, the shape of the ring current flow is more similar to the neutral Bi_6 prism. Molecular orbitals with an accumulation of electron density above the carbon triangle are shown in **Supplementary Figure 34**. Here, the close proximity of the three carbon atoms leads to the respective shape of the orbitals above the triangle. The similarity with the neutral Bi_6 prism is further illustrated by the NICS values of +0.15 ppm at the C_4 faces. Thus, there is only a local diatropic ring current in the C_3 and Bi_3 faces.

Supplementary Table 8: Current strengths in nA/T and NICS values in ppm of prismane. Calculated for the complete molecule and the upper or lower half.

	TPSS		cTPSS	
	Ring Current	NICS	Ring Current	NICS
Complete	+18.4	+1.5	+18.8	+1.1
Upper	+9.2	–33.3	+9.4	–33.8
Lower	+9.2	–33.3	+9.4	–33.8

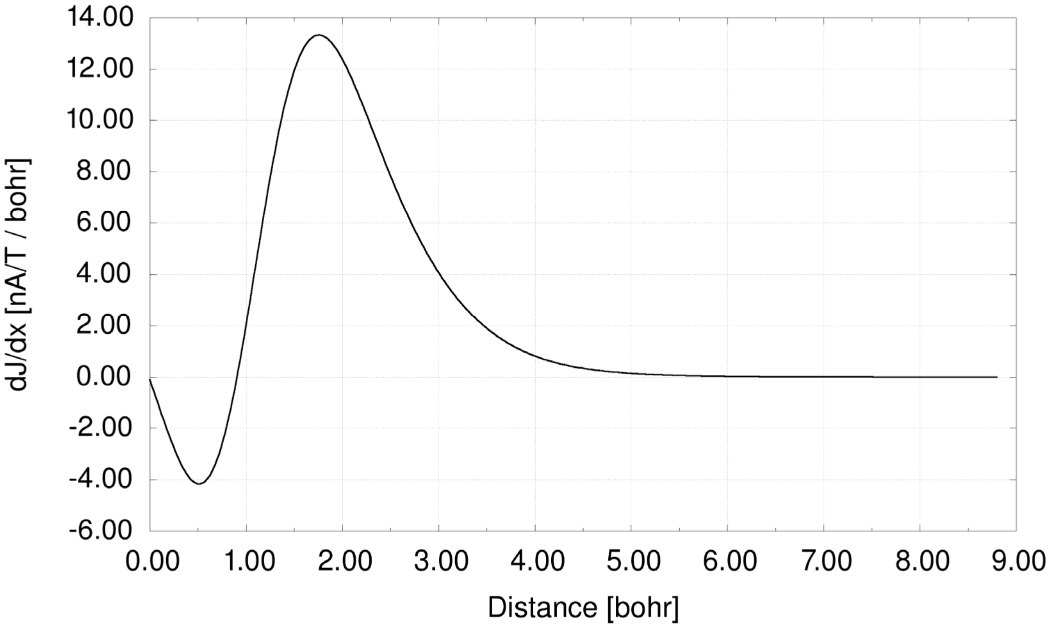

Supplementary Figure 33: Current density profile of the ring current strength calculated for prismane. The profile starts at the center of the prism and the C-C connection line is intersected at 0.8308 bohr. A negative sign indicates a paratropic contribution and a positive sign indicates a diatropic contribution.

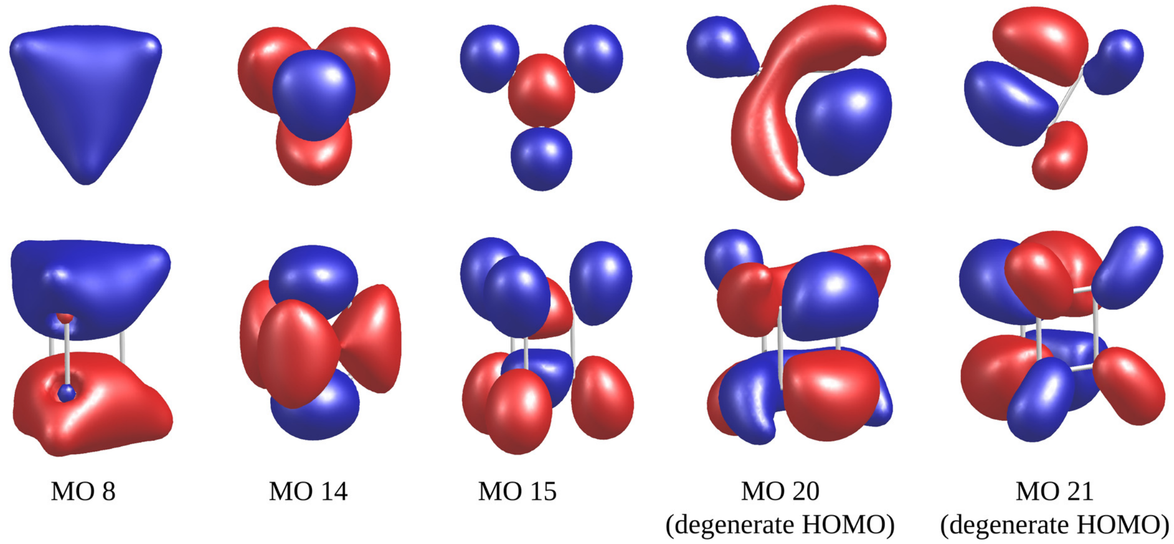

Supplementary Figure 34: Molecular orbitals of prismane with a substantial contribution above the carbon triangles. An isovalue of 0.04 was chosen for the visualization.

7. Powder X-Ray Diffractogram of the Starting Material $[K(crypt\text{-}222)]_2Bi_2$ (A)

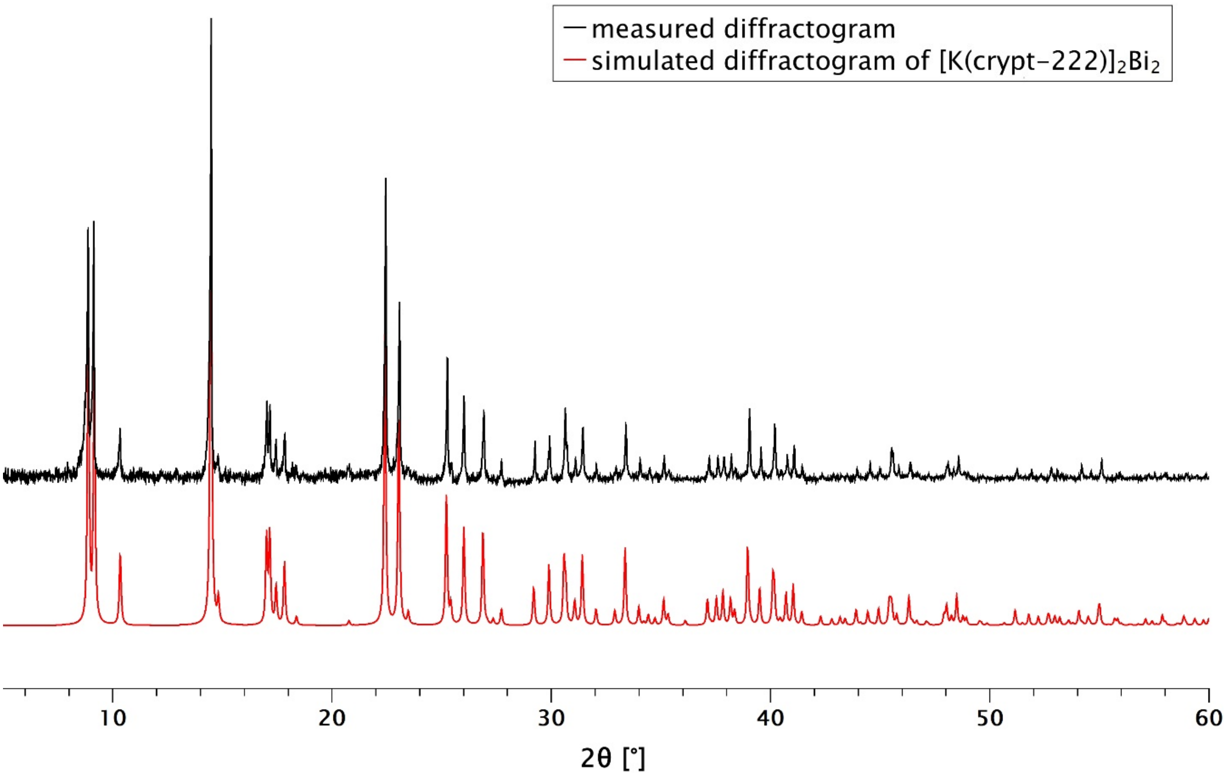

Supplementary Figure 35: Powder X-ray diffractogram of the starting material $[K(crypt\text{-}222)]_2Bi_2$ (A). The powder was investigated as used in the subsequent reactions. Methods are outlined in the main document.

8. References for the Supplementary Information

1. TURBOMOLE, Pre-Version 7.7 2022, a development of University of Karlsruhe and Forschungszentrum Karlsruhe GmbH 1989-2007, TURBOMOLE GmbH since 2007, available via https://www.turbomole.org (retrieved Februray 13, 2022).

2. Ahlrichs, R., Bär, M., Häser, M., Horn, H. & Kölmel, C. Electronic structure calculations on workstation computers: The program system turbomole. *Chem. Phys. Lett.* **162,** 165–169 (1989).

3. Furche, F., Ahlrichs, R., Hättig, C., Klopper, W., Sierka, M. & Weigend, F. Turbomole. *Wiley Interdiscip. Rev.: Comput. Mol. Sci.* **4,** 91–100 (2014).

4. Balasubramani, S. G. *et al.* TURBOMOLE: Modular program suite for *ab initio* quantum-chemical and condensed-matter simulations. *J. Chem. Phys.* **152,** 184107 (2020).

5. Tao, J., Perdew, J. P., Staroverov, V. N. & Scuseria, G. E. Climbing the Density Functional Ladder: Nonempirical Meta–Generalized Gradient Approximation Designed for Molecules and Solids. *Phys. Rev. Lett.* **93,** 146401 (2003).

6. Weigend, F. & Baldes, A. Segmented contracted basis sets for one- and two-component Dirac-Fock effective core potentials. *J. Chem. Phys.* **133,** 174102 (2010).

7. Metz, B., Stoll, H. & Dolg, M. Small-core multiconfiguration-Dirac-Hartree-Fock-adjusted pseudopotentials for post-d main group elements: Application to PbH and PbO. *J. Chem. Phys.* **113,** 2563–2569 (2000).

8. Treutler, O. & Ahlrichs, R. Efficient molecular numerical integration schemes. *J. Chem. Phys.* **102,** 346–354 (1995).

9. Treutler, O. Entwicklung und Anwendung von Dichtefunktionalmethoden. Ph.D. thesis (dissertation), Universität Karlsruhe (TH), Karlsruhe, Germany (1995).

10. Franzke, Y. J., Treß, R., Pazdera, T. M. & Weigend, F. Error-consistent segmented contracted all-electron relativistic basis sets of double- and triple-zeta quality for NMR shielding constants. *Phys. Chem. Chem. Phys.* **21,** 16658–16664 (2019).

11. Klamt, A. & Schüürmann, G. COSMO: a new approach to dielectric screening in solvents with explicit expressions for the screening energy and its gradient. *J. Chem. Soc., Perkin Trans.* **2,** 799–805 (1993).

12. Schäfer, A., Klamt, A., Sattel, D., Lohrenz, J. C. W. & Eckert, F. COSMO Implementation in TURBOMOLE: Extension of an efficient quantum chemical code towards liquid systems. *Phys. Chem. Chem. Phys*. **2,** 2187–2193 (2000).

13. Boys, S. F. Construction of some molecular orbitals to be approximately invariant for changes from one molecule to another. *Rev. Mod. Phys.* **32,** 296–299 (1960).

14. Armbruster, M. K., Weigend, F., van Wüllen, C. & Klopper, W. Self-consistent treatment of spin-orbit interactions with efficient Hartee-Fock and density functional methods. *Phys. Chem. Chem. Phys.* **10,** 1748–1756 (2008).

15. Reiter, K., Mack, F. & Weigend, F. Calculation of Magnetic Shielding Constants with meta- GGA Functionals Employing the Multipole-Accelerated Resolution of the Identity: Implementation and Assessment of Accuracy and Efficiency. *J. Chem. Theory Comput.* **14,** 191–197 (2018).

16. Gillhuber, S., Franzke, Y. J. & Weigend, F. Paramagnetic NMR Shielding Tensors and Ring Currents: Efficient Implementation and Application to Heavy Element Compounds. *J. Phys. Chem. A* **125,** 9707–9723 (2021).

17. Maximoff, S. N. & Scuseria, G. E. Nuclear magnetic resonance shielding tensors calculated with kinetic energy density-dependent exchange-correlation functionals. *Chem. Phys. Lett.* **390,** 408–412 (2004).

18. Dobson, J. F. Alternative expressions for the Fermi hole curvature. *J. Chem. Phys.* **98,** 8870–8872 (1993).

19. Bates, J. E. & Furche, F. Harnessing the meta-generalized gradient approximation for time-dependent density functional theory. *J. Chem. Phys.* **137,** 164105 (2012).

20. Furness, J. W. *et al.* Current Density Functional Theory Using Meta-Generalized Gradient Exchange-Correlation Functionals. *J. Chem. Theory Comput.* **11,** 4169–4181 (2015).

21. Schattenberg, C. J. & Kaupp, M. Effect of the Current-Dependence of Tau-Dependent Exchange-Correlation Functionals on Nuclear Shielding Calculations. *J. Chem. Theory Comput.* **17,** 1469–1479 (2021).

22. Holzer, C., Franzke, Y. J. & Kehry, M. Assessing the Accuracy of Local Hybrid Density Functional Approximations for Molecular Response Properties. *J. Chem. Theory Comput.* **17,** 2928–2947 (2021).

23. Franzke, Y. J. & Holzer, C. Impact of the current density on paramagnetic NMR properties. *J. Chem. Phys.* **157,** 031102 (2022).

24. Peng, D., Middendorf, N., Weigend, F. & Reiher, M. An efficient implementation of two-component relativistic exact-decoupling methods for large molecules. *J. Chem. Phys.* **138,** 184105 (2013).

25. Franzke, Y. J., Middendorf, N. & Weigend, F. Efficient implementation of one- and two-component analytical energy gradients in exact two-component theory. *J. Chem. Phys.* **148,** 104110 (2018).

26. Franzke, Y. J. & Weigend, F. NMR Shielding Tensors and Chemical Shifts in Scalar-Relativistic Local Exact Two-Component Theory. *J. Chem. Theory Comput.* **15,** 1028–1043 (2019).

27. Schleyer, P. v. R., Maerker, C., Dransfeld, A., Jiao, H. & Hommes, N. Nucleus-independent Chemical Shifts. *J. Am. Chem. Soc.* **118,** 6317–6318 (1996).

28. Jusélius, J., Sundholm, D. & Gauss, J. Calculation of current densities using gauge-including atomic orbitals. *J. Chem. Phys.* **121,** 3952–3963 (2004).

29. Taubert, S., Sundholm, D. & Jusélius, J. Calculation of spin-current densities using gauge-including atomic orbitals. *J. Chem. Phys.* **134,** 054123 (2011).

30. Wiberg, K. B. Application of the pople-santry-segal CNDO method to the cyclopropylcarbinyl and cyclobutyl cation and to bicyclobutane. *Tetrahedron* **24,** 1083–1096 (1968).

31. Jusélius, J., Straka, M. & Sundholm, D. Magnetic-Shielding Calculations on Al_4^{2-} and Analogues. A New Family of Aromatic Molecules? *J. Phys. Chem. A* **105,** 9939–9944 (2001).

32. Lin, Y.-C., Sundholm, D., Jusélius, J., Cui, L.-F., Li, Xi, Zhai, H.-J. & Wang, L.-S. Experimental and Computational Studies of Alkali-Metal Coingage-Metal Clusters. *J. Phys. Chem. A* **110,** 4244–4250 (2006).

33. Staroverov, V. N., Scuseria, G. E., Tao, J. & Perdew, J. P. Comparativ assessment of a new nonempirical density functional: Molecules and hydrogen-bond complexes. *J. Chem. Phys.* **119,** 12129–12137 (2003).

34. Becke, A. D. Density-functional exchange-energy approximation with correct asymptotic behavior. *Phys. Rev. A.* **38,** 3098–3100 (1988).

35. Lee, C., Yang, W. & Parr, R. G. Development of the Colle-Salvetti correlation-energy formula into a functional of the electron density. *Phys. Rev. B.* **37,** 785–789 (1988).

36. Becke, A. D. A new mixing of Hartee–Fock and local density-functional theories. *J. Chem. Phys.* **98,** 1372–1377.

37. Perdew, J. P., Burke, K. & Ernzerhof, M. Generalized Gradient Approximation Made Simple. *Phys. Rev. Lett.* **77,** 3865–3868 (1996).

38. Adamo, C. & Barone, V. Toward reliable density functional methods without adjustable parameters: The PBE0 model. *J. Chem. Phys.* **110,** 6158–6170 (1999).

39. Vydrov, O. A. & Scuseria, G. E. Assessment of a long range corrected hybrid functional. *J. Chem. Phys.* **125,** 234109 (2006).

40. Chai, J.-D. & Head-Gordon, M. Long-range corrected hybrid density functionals with damped atom-atom dispersion corrections. *Phys. Chem. Chem. Phys.* **10,** 6615–6620 (2008).

41. Eulenstein, A. R. *et al.* Substantial π-aromaticity in the anionic heavy-metal cluster $[Th@Bi_{12}]^{4-}$. *Nat. Chem.* **13,** 149–155 (2021).

42. Monaco, C., Zanasi, R., Pelloni, S. & Lazzaretti, P. Relative weight of sigma and pi ring current in a few simple monocycles. *J. Chem. Theory Comput.* **6,** 3343–3351 (2010).

43. Fowler, P., Havenith, R. W. A. & Steiner, W. Unconventional ring current in an 'all-metal aromatic', Al_4^{2-}. *Chem. Phys. Lett.* **342,** 85–90 (2001).

44. Lin, Y. C., Jusélius, J, Sundholm, D. & Gauss, J. Magnetically induced current densities in Al_4^{2-} and Al_4^{4-} species studied at the coupled-cluster level. *J. Chem. Phys.* **122,** 214308 (2005).

45. Sundholm, D., Fliegl, H. & Berger, R. J. Calculations of magnetically induced current densities: theory and applications. *Wiley Interdiscip. Rev.: Comput. Mol. Sci.* **6,** 639–678 (2016).

46. Qiao, L., Chen, D., Zhu, J., Muñoz-Castro & Sun, Z.-M. $[Bi_6Mo_3(CO)_9]^{4-}$: a multiple local σ-aromatic cluster containing a distorted Bi_6 triangular prism. *Chem. Commun.* **57,** 3656–3659 (2021).

47. Reed, A. E., Weinstock, R. B. & Weinhold, F. Natural population analysis. *J. Chem. Phys.* **83,** 735–746 (1985).
